# Effect of High-Dose Selenium on Postoperative Organ Dysfunction and Mortality in Cardiac Surgery Patients: The SUSTAIN CSX Randomized Clinical Trial

**DOI:** 10.1001/jamasurg.2022.6855

**Published:** 2023-01-11

**Authors:** Christian Stoppe, Bernard McDonald, Patrick Meybohm, Kenneth B. Christopher, Stephen Fremes, Richard Whitlock, Siamak Mohammadi, Dimitri Kalavrouziotis, Gunnar Elke, Rolf Rossaint, Philipp Helmer, Kai Zacharowski, Ulf Günther, Matteo Parotto, Bernd Niemann, Andreas Böning, C. David Mazer, Philip M. Jones, Marion Ferner, Yoan Lamarche, Francois Lamontagne, Oliver J. Liakopoulos, Matthew Cameron, Matthias Müller, Alexander Zarbock, Maria Wittmann, Andreas Goetzenich, Erich Kilger, Lutz Schomburg, Andrew G. Day, Daren K. Heyland

**Affiliations:** 1 University of Ottawa Heart Institute, Ottawa, Ontario, Canada; 2 Department of Anaesthesiology, Intensive Care, Emergency, and Pain Medicine, University Hospital Wuerzburg, Wuerzburg, Germany; 3 Division of Renal Medicine, Brigham and Women’s Hospital, Boston, Massachusetts; 4 Sunnybrook Research Institute, Toronto, Ontario, Canada; 5 Hamilton Health Sciences, Hamilton, Ontario, Canada; 6 Quebec Heart and Lung Institute, Laval University, Quebec City, Quebec, Canada; 7 University Hospital Schleswig-Holstein, Kiel, Germany; 8 University Hospital Aachen, Aachen, Germany; 9 University Hospital Frankfurt, Frankfurt, Germany; 10 Oldenburg Clinic, University of Oldenburg, Oldenburg, Germany; 11 Department of Anesthesiology and Pain Medicine, Toronto General Hospital, Toronto, Ontario, Canada; 12 Division of Critical Care Medicine, Department of Anesthesia and Interdepartmental University of Toronto, Toronto, Ontario, Canada; 13 University Hospital of Giessen, Giessen, Germany; 14 Li Ka Shing Knowledge Institute, St Michael’s Hospital, Toronto, Ontario, Canada; 15 Department of Anesthesiology and Pain Medicine, Department of Physiology, University of Toronto, Toronto, Ontario, Canada; 16 London Health Sciences Centre, London, Ontario, Canada; 17 University Medical Center of the Johannes Gutenberg-University Mainz, Mainz, Germany; 18 Hôpital du Sacré-Coeur de Montréal, Montreal, Quebec, Canada; 19 Montreal Heart Institute, Montreal, Quebec, Canada; 20 Hôpital Fleurimont (CHUS), Sherbrooke, Quebec, Canada; 21 Department of Cardiothoracic Surgery, Heart Center, University Hospital of Cologne, Cologne, Germany; 22 Jewish General Hospital, Montreal, Quebec, Canada; 23 University Heart Center Freiburg Bad Krozingen, Bad Krozingen, Germany; 24 University Hospital Münster, Münster, Germany; 25 University Hospital Bonn, Bonn, Germany; 26 now with Abiomed Europe GmbH, Aachen, Germany; 27 Ludwig Maximilian University of Munich, Munich, Germany; 28 Institute for Experimental Endocrinology, Charité-Universitätsmedizin Berlin, Berlin, Germany; 29 Clinical Evaluation Research Unit, Queen’s University, Kingston, Ontario, Canada; 30 Department of Critical Care Medicine, Queen’s University, Kingston, Ontario, Canada

## Abstract

**Importance:**

Selenium contributes to antioxidative, anti-inflammatory, and immunomodulatory pathways, which may improve outcomes in patients at high risk of organ dysfunctions after cardiac surgery.

**Objective:**

To assess the ability of high-dose intravenous sodium selenite treatment to reduce postoperative organ dysfunction and mortality in cardiac surgery patients.

**Design, Setting, and Participants:**

This multicenter, randomized, double-blind, placebo-controlled trial took place at 23 sites in Germany and Canada from January 2015 to January 2021. Adult cardiac surgery patients with a European System for Cardiac Operative Risk Evaluation II score–predicted mortality of 5% or more or planned combined surgical procedures were randomized.

**Interventions:**

Patients were randomly assigned (1:1) by a web-based system to receive either perioperative intravenous high-dose selenium supplementation of 2000 μg/L of sodium selenite prior to cardiopulmonary bypass, 2000 μg/L immediately postoperatively, and 1000 μg/L each day in intensive care for a maximum of 10 days or placebo.

**Main Outcomes and Measures:**

The primary end point was a composite of the numbers of days alive and free from organ dysfunction during the first 30 days following cardiac surgery.

**Results:**

A total of 1416 adult cardiac surgery patients were analyzed (mean [SD] age, 68.2 [10.4] years; 1043 [74.8%] male). The median (IQR) predicted 30-day mortality by European System for Cardiac Operative Risk Evaluation II score was 8.7% (5.6%-14.9%), and most patients had combined coronary revascularization and valvular procedures. Selenium did not increase the number of persistent organ dysfunction–free and alive days over the first 30 postoperative days (median [IQR], 29 [28-30] vs 29 [28-30]; *P* = .45). The 30-day mortality rates were 4.2% in the selenium and 5.0% in the placebo group (odds ratio, 0.82; 95% CI, 0.50-1.36; *P* = .44). Safety outcomes did not differ between the groups.

**Conclusions and Relevance:**

In high-risk cardiac surgery patients, perioperative administration of high-dose intravenous sodium selenite did not reduce morbidity or mortality. The present data do not support the routine perioperative use of selenium for patients undergoing cardiac surgery.

**Trial Registration:**

ClinicalTrials.gov Identifier: NCT02002247

## Key Points

**Question:**

Does high-dose selenium therapy impact outcomes in patients at high risk of organ dysfunction and death after cardiac surgery?

**Findings:**

In this randomized clinical trial of 1416 adult cardiac surgery patients, a high dose of sodium selenite compared with placebo did not result in a significant different morbidity or mortality.

**Meaning:**

High-dose sodium selenite was not effective in reducing the development of organ dysfunction and death in high-risk cardiac surgery patients.

## Teaser

This randomized clinical trial assesses the ability of high-dose intravenous sodium selenite treatment to reduce postoperative organ dysfunction and mortality in cardiac surgery patients.

## Introduction

Cardiac surgery is performed worldwide in an estimated 1 million patients per year.^1^ Death and morbidity requiring immediate postoperative life-supportive therapy currently occur in nearly 20% of cardiac surgery patients. Prolonged life-supportive therapies negatively impact longer-term survival and quality of life.^2,3^ Major morbidity in cardiac surgery occurs in the context of oxidative stress from ischemia-reperfusion injury following operative global ischemic cardioplegic arrest of the heart or embolic events.^4^ Such oxidative stress triggers an intense inflammatory response marked by endothelial dysfunction, microvascular thrombosis, and injury of all major organ systems resulting in prolonged intensive care unit (ICU) length of stay.^5^ Novel therapies to reduce the morbidity and mortality associated with high-risk cardiac surgery are needed.

A potential way to reduce organ dysfunction is supplementation with the essential trace element selenium, which contributes to anti-inflammatory and immunomodulatory pathways and is a constituent of the active site of multiple antioxidant enzymes.^6,7^ In cardiac surgery patients, low selenium levels are associated with postoperative multiorgan failure.^8^ Several smaller studies suggested significant clinical benefits associated with a selenium supplementation in cardiac surgery patients.^9,10,11,12,13,14^ Of these, some showed that perioperative supplementation of high-dose selenium prevents the dramatic intraoperative decrease of circulating selenium levels,^9,10^ which leads to less oxidative stress, reduced need of postoperative vasoactive support, less myocardial injury, fewer organ dysfunctions, and reduced hospital length of stay.^11^ These findings strengthen the hypothesis that this key nutrient can ameliorate oxidative stress and improve outcomes. Yet, a higher level of evidence was needed to inform clinical guidelines regarding the use of selenium in cardiac surgery patients. Accordingly, we conducted an international, prospective, double-blind, randomized, placebo-controlled trial to evaluate the efficacy of high-dose intravenous selenium in patients undergoing cardiac surgery with cardiopulmonary bypass (CPB) at increased risk for organ dysfunction or death. Based on the existing evidence, we hypothesized that, compared with placebo, intravenous selenium would lead to lower morbidity and mortality as well as reduced new postoperative persistent organ dysfunctions and/or death after cardiac surgery.

## Methods

### Trial Design and Participants

We conducted an international, double-blind, randomized placebo-controlled trial, the Sodium Selenite Administration in Cardiac Surgery Trial (SUSTAIN CSX) trial, at 23 sites in Canada and Germany.^15^ The trial protocol and statistical analysis plan are available in Supplement 1 and have been previously published.^15^ The protocol was approved by the ethics committees of Queens University, Canada, and RWTH Aachen University, Germany, the German Federal Institute for Drugs and Medical Devices, and by all participating centers. Consolidated Standards of Reporting Trials (CONSORT) reporting guideline was followed. Each patient gave written informed consent to participate in the study before surgery. All sites that participated in the data collection are listed in the eMethods in Supplement 2. Patients were screened from January 14, 2015, through January 11, 2021.

Patients 18 years or older were eligible if they were scheduled to undergo elective or urgent cardiac surgery with the use of CPB and cardioplegic arrest and were at higher risk of perioperative morbidity and mortality as defined by the European System for Cardiac Operative Risk Evaluation (EuroSCORE) II predicted operative mortality of 5% or more or if combined surgical procedures were planned.^16^ Patients with EuroSCORE II of 5% or more who undergo cardiac surgery are shown to have an excessive systemic inflammatory response, a pronounced decrease of selenium during surgery, and a prolonged ICU course.^8,9,17^ Data on race and ethnicity were collected by self-report.

Key exclusion criteria were hypersensitivity to sodium selenite or to any of the diluent vehicle, total bilirubin more than 2.0 mg/dL (to convert to micromoles per liter, multiply by 17.104), disabling neuropsychiatric disorders, pregnancy, lactation, current antioxidant use, cardiac transplant, planned ventricular assist device implantation, correction of complex congenital anomalies, or planned use of hypothermic cardiocirculatory arrest. The complete inclusion and exclusion criteria are noted in the trial protocol in Supplement 1 and in the eMethods in Supplement 2.

### Randomization and Masking

The participants were randomly assigned in a 1:1 ratio to the intervention and placebo groups, respectively. Randomization was performed through a central, password-protected, web-based system with an audit trail that has been used for several prior international studies. Randomization was stratified by site in permuted blocks of size 4. The randomization list was prepared by the trial statistician (A.G.D.) who did not disclose the block size until after study enrollment was complete. Access to unblinded data was limited to the independent data monitoring committee until the trial database was completed and locked. Outcomes were not examined by treatment assignment until after the statistical analysis plan was finalized and published. To blind patients, investigators, and health care practitioners, selenium and placebo were prepared and supplied by an industry partner (Biosyn) in a way to maintain blinding. Selenium and placebo were provided similar in appearance, consistency, volume, and smell; study researchers were blinded to the treatment assignment at the time of enrollment to exclude selection bias.

### Procedures

According to the group allocation, patients either received 2000 μg/L (to convert to micromoles per liter, multiply by 0.0127) of intravenous selenium (sodium selenite; Selenase) within 30 minutes after induction of anesthesia and prior to initiation of CPB, then 2000 μg/L of intravenous selenium immediately on admission to the postoperative ICU, then 1000 μg/L of intravenous selenium each successive morning while in ICU or placebo at the same time points for a maximum of 10 days (eFigure 1 in Supplement 2). This supplementation strategy was previously demonstrated to be safe and effective in these patients.^8,9^

In general, enrolled patients were followed up daily throughout the ICU stay. Baseline demographics (ie, age, sex, height, weight, diagnosis, EuroSCORE II, Sequential Organ Failure Assessment score, cardiovascular medication) and functional status (ie, Barthel Activity of Daily Living, Clinical Frailty Scale, Health Related Quality of Life with Short Form-36) were recorded before surgery. If possible, reevaluation of functional status was made 3 and 6 months after randomization. Moreover, data on surgical procedure and duration, perioperative hemodynamic profile (ie, heart rate, systemic and pulmonary blood pressure, central venous pressure, cardiac output), and laboratory measures (ie, complete blood cell count, international normalized ratio, blood lactate, creatinine) recorded postanesthetic induction, upon ICU admission, and at the first postoperative day were assessed. Additionally, the occurrence of surgical and cardiovascular complications (eg, myocardial infarction, arrhythmias, stroke/cerebral vascular accident, deep sternal wound infection) and hospital-acquired infections was monitored throughout the study period, while ICU and hospital length of stay were assessed at discharge.

### Outcome Measures

The primary outcome was the number of days alive and free of persistent organ dysfunction within the first 30 days after surgery.^18,19^ Persistent organ dysfunction was defined daily as the need for life-sustaining therapies that developed postoperatively at any time during the day. Life-sustaining therapies included mechanical ventilation, noninvasive ventilation, any vasopressor therapy, mechanical circulatory support, continuous kidney replacement therapy, or intermittent hemodialysis. Patients who died within 30 days after surgery were recorded as having 0 persistent organ dysfunction–free days. Secondary outcomes included 30-day mortality, hospital-acquired infections, cardiovascular complications, duration of mechanical ventilation, incidence of postoperative delirium (measured by the Confusion Assessment Method for the ICU), ICU length of stay, hospital readmission rates, hospital length of stay, 6-month survival, and quality of life. For full definitions and complete list of laboratory and outcome measures, see the eMethods in Supplement 2. After hospital discharge, follow-up was performed at 3 and 6 months after randomization. To assess the safety of the study medication, patients were monitored daily for the occurrence of unexpected serious adverse events until death or discharge.

### Statistical Analysis

We estimated that a total sample size of 1400 participants followed up for 30 days would provide 90% power at a 2-sided α = .05 to detect a difference in persistent organ dysfunction–free days following selenium supplementation if the intervention caused a 20% relative increase in the daily rate of liberation from life-sustaining therapy but no change in mortality compared with the control arm. This effect size corresponded to a mean increase of 1.5 persistent organ dysfunction–free days (from 23.2 to 24.7 days). These estimates were based on 10 000 simulations where the control arm followed the distribution of a sample of 170 prior patients who met the current inclusion criteria.

For the primary outcome, the van Elteren test stratified by site was used to calculate the *P* value for comparing persistent organ dysfunction plus death between groups. The within-site concordance index (C index) was also used to summarize the effect size. Quartiles of persistent organ dysfunction–free days were reported by arm. Prespecified subgroup analyses and further statistical analysis were performed as described in the eMethods in Supplement 2.

## Results

### Participants

**Figure 1.**
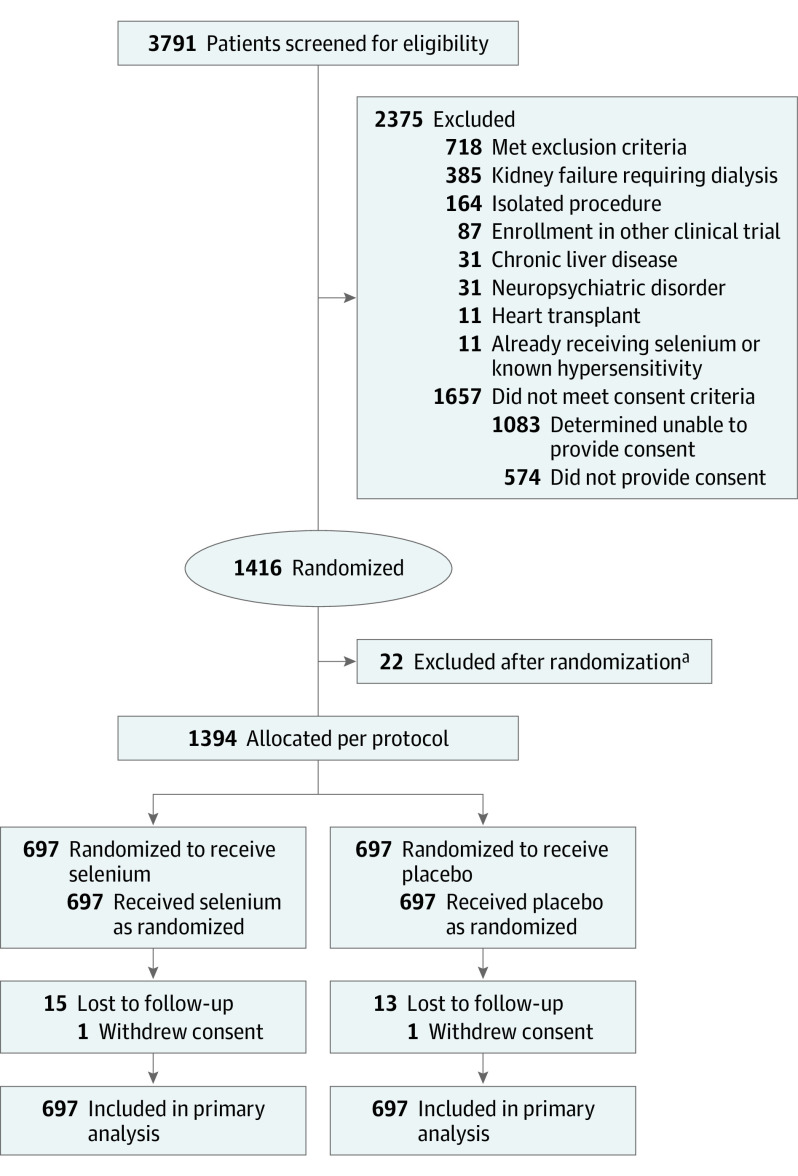
Flowchart of Patient Enrollment and Randomization Patients could be ineligible for more than 1 exclusion criterion. ^a^Excluded because their operation or eligibility changed; these patients did not receive selenium or placebo.

A total of 3791 patients were screened for eligibility; 1416 patients provided written consent and were randomized. Twenty-two randomized patients were excluded without ever receiving the allocated intervention; the majority of these were because the planned surgery was canceled or changed to a procedure not eligible per inclusion criteria. Thus, this primary modified intent-to-treat analysis includes 1394 patients with 697 in each treatment group (Figure 1). Over the first 30 days, there were no patients lost to follow-up and no missing outcomes of interest. At 6 months, 15 patients were lost to follow-up and 1 withdrew consent in the selenium group, while 13 patients were lost to follow-up and 2 withdrew consent in the placebo group (Figure 1).

### Baseline Characteristics

The characteristics of patients at baseline were similar in the selenium and placebo group (Table 1). Overall, 27 patients (1.9%) were Asian or Pacific Islander, 3 (0.2%) were Black/African/African American, 7 (0.5%) were Hispanic; 4 (0.3%) were Native, 1345 (96.5%) were White, and 8 (0.6%) specified as other. A total of 871 trial participants (62.5%) were from Canada and 1150 surgeries (82.5%) were scheduled as elective. The mean (SD) age of participants was 68.2 (10.4) years, the group comprised 1043 male individuals (74.8%), and had a median (IQR) 30-day predicted mortality based on EuroSCORE II of 8.7% (5.6%-14.9%). A total of 907 surgical procedures (65.1%) were performed included coronary artery bypass grafting, while 1063 (89.7%) received at least 1 valvular intervention (repair or replacement) and 21 patients (3%) received a surgical procedure at the isloated thoracic aorta. The mean (SD) duration of CPB was 143.6 (59.9) minutes and severity of illness on ICU admission as assessed by mean (SD) Sequential Organ Failure Assessment score was identical between the 2 groups at 9.8 (2.8).

**Table 1.** Baseline Demographics and Operative Characteristics^a^

Characteristic	No. (%)	
	Selenium group	Placebo group
No.	697	697
Age, mean, (SD), y	68.0 (10.0)	68.5 (10.8)
Male	513 (74)	530 (76)
Female	184 (26)	167 (24)
BMI, mean (SD) [range]	28.9 (5.3) [16.9-49.8]	28.2 (5.2) [15.8-56.0]
Race and ethnicity		
Asian or Pacific Islander	13 (2)	14 (2)
Black/African	1 (0)	2 (0)
Hispanic	5 (1)	2 (0)
Native	3 (0)	1 (0)
White	672 (96)	673 (97)
Other (specify)^b^	3 (0)	5 (1)
Site		
Canada	436 (63)	435 (62)
Germany	261 (37)	262 (38)
Surgery type		
Elective	576 (83)	574 (82)
Urgent	120 (17)	119 (17)
Emergent	1 (0)	4 (1)
EuroSCORE II, median (IQR)	8.8 (5.5-15.2)	8.5 (5.7-14.3)
Previous cardiac surgery	54 (7.7)	53 (7.6)
Previous myocardial infarction	124 (17.8)	108 (15.5)
LVEF, median (IQR)	55.0 (50.0-60.0)	55.0 (50.0-60.0)
%EF		
≤40	57 (8)	76 (11)
≤35	31 (4)	49 (7)
Moderately impaired kidney function	210 (30)	218 (31)
Severely impaired kidney function	38 (6)	49 (7)
Chronic kidney disease and undergoing dialysis	1 (0)	0 (0)
Diabetes	171 (25)	173 (25)
Atrial fibrillation	148 (21)	150 (22)
Charlson Comorbidity Index, mean (SD)	1.4 (1.4)	1.5 (1.4)
Clinical Frailty Score of ≥4	157 (23)	147 (21)
CBC and chemistry, mean (SD) [range]		
Hemoglobin (lowest), g/dL	12.8 (2.2) [6.1-18.9]	12.8 (2.1) [6.0-18.1]
Platelet count (lowest), ×10^3^/µL	150.3 (53.6) [39.0-410.0]	147.3 (55.3) [36.0-386.0]
WBC, /µL		
Highest	8200 (9000) [2000-218 000]	7800 (3400) [2400-44 900]
Lowest	7400 (9800) [1400-21 700]	7100 (2300) [2400-19 700]
Albumin (lowest), g/dL	3.8 (0.6) [1.1-6.5]	3.7 (0.6) [1.8-6.7]
Creatinine (highest), mg/dL	1.0 (0.4) [0.4-7.0]	1.0 (0.4) [0.5-4.0]
Preoperative medical therapy, mean (SD) [range]	128.3 (21.8) [61.0-188.0]	128.0 (21.4) [60.0-181.0]
ACE inhibitor	242 (34.7)	234 (33.6)
Angiotensin receptor blockers	143 (20.5)	138 (19.8)
Amiodarone	14 (2.0)	21 (3.0)
β-Blockers	390 (56.0)	372 (53.4)
Calcium channel blockers	190 (27.3)	164 (23.5)
Statins	406 (58.2)	425 (61.0)
Corticosteroids	15 (2.2)	21 (3.0)
Operative characteristics		
CABG surgery only	26 (4)	28 (4)
CABG + valve/multivalve	406 (58)	407 (58)
CABG + other nonvalve	21 (3)	19 (3)
Isolated valve	40 (6)	50 (7)
Multivalve	82 (12)	78 (11)
Isolated thoracic aorta	21 (3)	21 (3)
Other	5 (1)	3 (0)
Duration of cardiopulmonary bypass, mean (SD), min	141.8 (57.9)	145.7 (61.4)
Duration of aortic clamping, mean (SD) [range], y	107.6 (44.7) [10.0-394.0]	110.1 (47.0) [4.3-365.0]

Abbreviations: ACE, angiotensin-converting enzyme; BMI, body mass index (calculated as weight in kilograms divided by height in meters squared); CABG, coronary artery bypass grafting; CBC, complete blood cell count; EF, ejection fraction; EuroSCORE, European System for Cardiac Operative Risk Evaluation; LVEF, left ventricular ejection fraction; WBC, white blood cell count.

SI conversion factors: To convert albumin to grams per liter, multiply by 10; creatinine to micromoles per liter, multiply by 88.4; hemoglobin to grams per liter, multiply by 10; platelet count to ×10^9^ per liter, multiply by 1; WBC to ×10^9^ per liter, multiply by 0.001.

^a^ Moderate kidney disease is categorized as creatinine clearance of 0.6 to 1.0 mg/dL and severe kidney disease as creatinine clearance less than 0.6 mg/dL. There were no descriptively significant differences between the 2 groups.

^b^ The present ethnicity list is used historically. Specific races and ethnicites in the other group were not reported.

### Compliance With Study Protocol

Only 228 doses of investigational product were missed of 7173 doses indicated by protocol, giving an overall compliance rate of 96.8%. There was no meaningful difference in compliance with dosing as per protocol between the selenium and placebo groups (eTable 1 in Supplement 2). Seventeen patients received considerable doses of *N*-acetylcysteine while 8 received high-dose vitamin C supplementation; there were no differences in this adjuvant antioxidant medication use between selenium and placebo groups (eTable 2 in Supplement 2).

### Trial Outcomes

**Figure 2.**
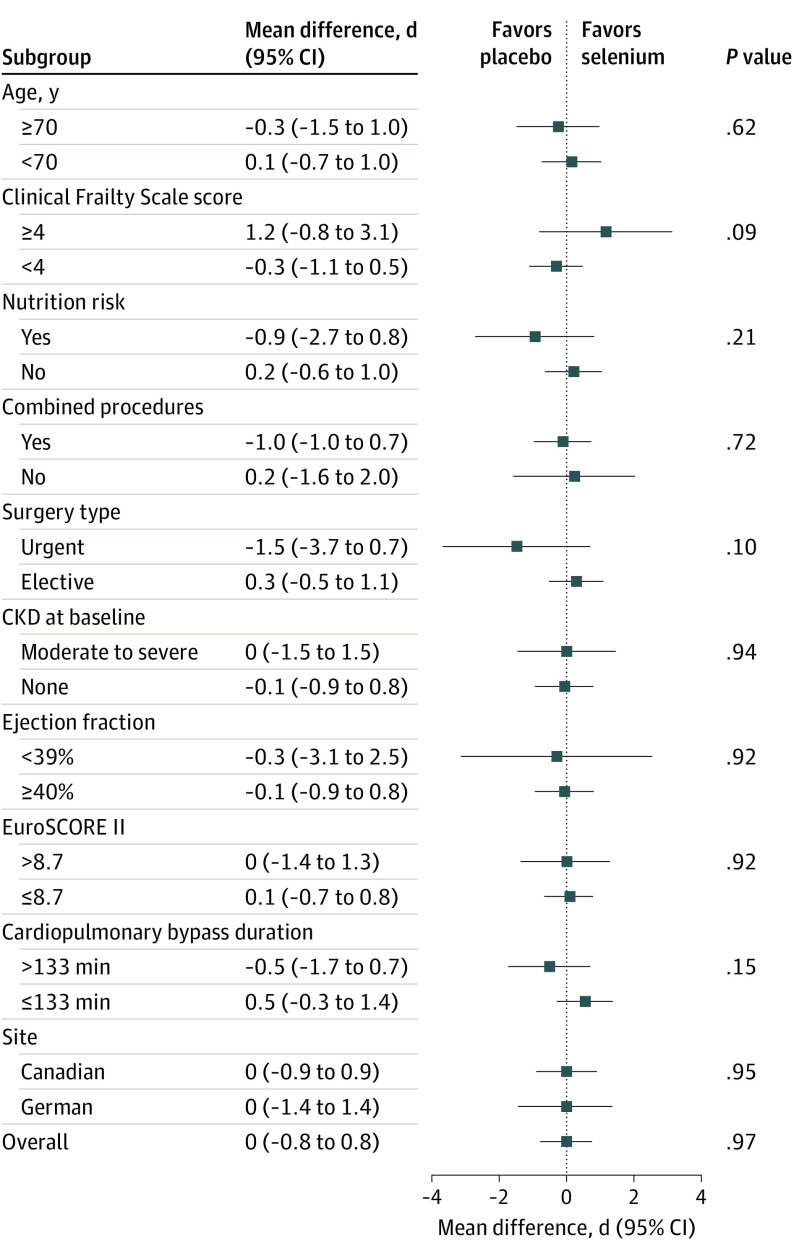
Primary Outcome (Persistent Organ Dysfunction–Free Days in 30 Days) by Subgroups CKD indicates chronic kidney disease; EuroSCORE, European System for Cardiac Operative Risk Evaluation.

The overall daily frequency of the components of persistent organ dysfunction and occurrence of death are shown in eTable 3 in Supplement 2. The overall rate of persistent organ dysfunction plus death was 31.1% (n = 434) at day 2, 6.7% (n = 94) at day 14 and 6.2% (n = 86) by day 30 (Table 2). The primary outcome of days alive and persistent organ dysfunction free in the first 30 days after surgery was virtually identical in both surgery groups with a median (IQR) of 29 (28-30) days in both groups, and a corresponding site-stratified concordance index of 0.51 (95% CI, 0.48-0.54; *P* = .45). A sensitivity analysis excluding patients with missed intravenous selenium doses did not change these findings. Each of the individual components of persistent organ dysfunction demonstrated an equal distribution and duration across both groups (eTable 4 in Supplement 2). There were no significant treatment by subgroup interactions or significant treatment effects within any of the prespecified subgroups (Figure 2). Patients with a Clinical Frailty Scale score of 4 or higher showed a trend favoring the selenium arm (odds ratio, 1.2; 95% CI, −0.8 to 3.1; *P* = .09), whereas patients with urgent procedures and prolonged CPB time tended to favor placebo.

**Table 2.** Primary and Secondary Outcomes^a^

Outcome	Mean (SD)		OR, HR, or mean differences (95% CI)	*P* value^b^
	Selenium group	Placebo group		
No.	697	697		
Primary outcome				
Persistent organ dysfunction–free days and alive at postoperative day 30, median (IQR)	29.0 (28.0 to 30.0)	29.0 (28.0 to 30.0)	0.0 (−0.7 to 0.7)^c^	.45
Secondary outcome				
Mortality, No. (%)				
30 d	29 (4.2)	35 (5.0)	0.82 (0.50 to 1.36)^d^	.44
6 mo	93 (90 to 94)	93 (90 to 94)	1.00 (0.68 to 1.47)^d^	.98
Days to discharge alive, median (IQR)				
From ICU	2.2 (1.1 to 5.2)	2.2 (1.1 to 5.0)	0.97 (0.86 to 1.08)^e^	.52
From hospital	9.0 (6.1 to 15.0)	8.6 (6.1 to 14.3)	0.96 (0.86 to 1.07)^e^	.43
Patients with readmission, No. (%)				
ICU	29 (4.2)	46 (6.6)	0.60 (0.37 to 0.97)^d^	.04
Hospital readmission	146 (20.9)	119 (17.1)	1.29 (0.97 to 1.71)^d^	.33
Other clinical outcomes associated with inflammatory state				
Platelet count (lowest), ×10^3^/µL	695 (139.3)	696 (135.3)	2.7 (−2.1 to 7.5)^c^	.07
WBC (highest), /µL	696 000 (11 700)	696 000 (11 600)	0.1 (−0.2 to 0.5)^c^	.56
Creatinine (highest), mg/dL	7.9 (1.0)	7.9 (1.0)	3.0 (−2.0 to 8.0)^c^	.26
Albumin (lowest), g/dL	39.7 (3.8)	38.4 (3.7)	0.1 (−0.5 to 0.7)^c^	.54
Total bilirubin, mg/dL	23.6 (0.8)	23.0 (0.7)	−0.7 (−3.9 to 2.4)^c^	.91
Creatine kinase–MB fraction, ng/mL	102 (22.7)	93 (24.3)	3.0 (−12.3 to 18.3)^c^	.72
Blood lactate (highest), mg/dL	5576.6 (19.8)	5558.6 (19.8)	0.0 (−0.2 to 0.3)^c^	.49
Delirium while in ICU, No. (%)	112 (16.1)	127 (18.2)	0.86 (0.65 to 1.14)^d^	.28
Clinically significant atrial fibrillation, No. (%)	113 (16.2)	84 (12.1)	1.59 (0.90 to 2.79)^d^	.11
Hospital-acquired infections, No. (%)	62 (8.9)	58 (8.3)	1.07 (0.73 to 1.56)^d^	.72

Abbreviations: HR, hazard ratio; ICU, intensive care unit; OR, odds ratio; WBC, white blood cell count.

SI conversion factors: To convert albumin to grams per liter, multiply by 10; blood lactate to millimoles per liter, multiply by 0.111; creatinine to micromoles per liter, multiply by 88.4; creatine kinase–MB fraction to micrograms per liter, multiply by 1; platelet count to ×10^9^ per liter, multiply by 1; total bilirubin to micromoles per liter, multiply by 17.104; WBC to ×10^9^ per liter, multiply by 0.001.

^a^ Maximum Confusion Assessment Method for the ICU score and mean laboratory values were calculated over the treatment period for each patent before applying statistical summaries.

^b^ Delirium tested by logistic generalized linear mixed-effects model with random site effect. Other outcomes tested by the van Elteren test stratified by site.

^c^ Mean differences are reported.

^d^ ORs are reported.

^e^ HRs are reported.

The observed 30-day mortality rate was 4.2% (n = 29) in the selenium and 5.0% (n = 35) in the placebo group (odds ratio, 0.82; 95% CI, 0.50-1.36; *P* = .44). eFigure 2 in Supplement 2 shows that the 6-month survival rate was nearly identical between treatment groups (hazard ratio, 1.00; 95% CI, 0.68-1.47; *P* = .98).

The overall median (IQR) time to discharge alive from the ICU and hospital was 2.2 (1.1-5.1) days and 8.9 (6.2-14.9) days, respectively, with no statistically significant difference between groups for either measure (Table 2). There were no significant differences between groups for secondary outcomes of ICU diagnoses thought to be related in part to inflammatory state and posited as potentially amenable to selenium administration such as delirium, atrial fibrillation, and hospital-acquired infections (Table 2; eTable 5 in Supplement 2).

In total, 57 patients (8.2%) and 60 patients (8.6%) in the selenium and placebo groups had at least 1 serious adverse event, respectively. The most common class of serious adverse events was cardiac disorders occurring in 29 patients (4.2%) and 30 patients (4.3%) in the selenium and placebo groups, respectively (eTable 6 in Supplement 2).

### Laboratory Subanalysis: Selenium, Selenoprotein, and Glutathione Peroxidase Levels

**Figure 3.**
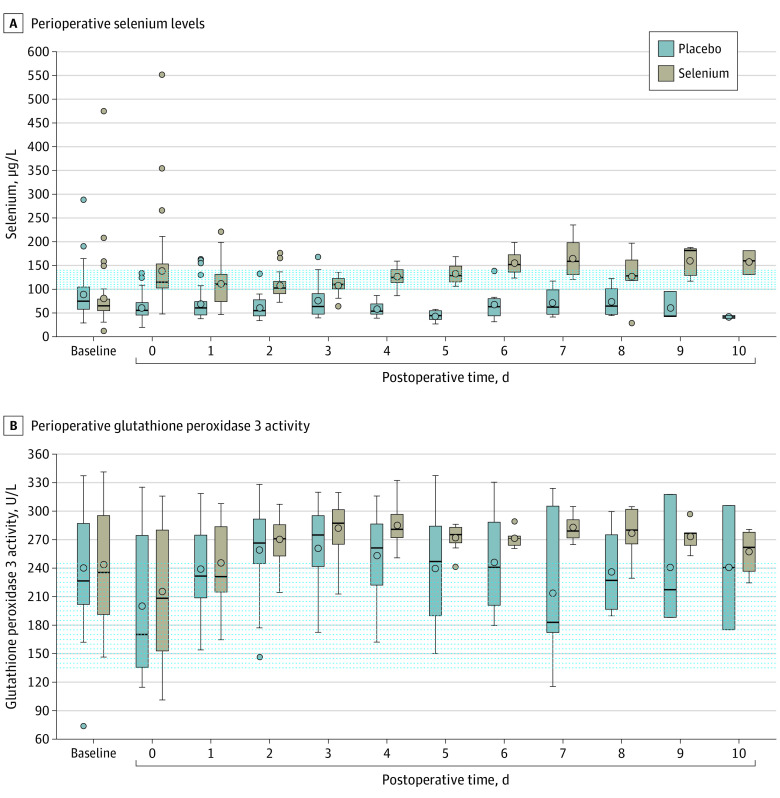
Perioperative Selenium Levels and Perioperative Activity of Glutathione Peroxidase 3 A, Perioperative selenium levels are compared by treatment and time points. The dotted lines indicate the reference range of selenium. Selenium concentrations below 70 μg/L indicate a selenium deficiency and selenium levels below 45.9 μg/L a severe selenium deficiency.^20^ B, Perioperative activity of glutathione peroxidase 3 is compared by treatment and time. Whiskers indicate upper and lower values, lines in boxes are median values, and circles are outliers. The dotted line indicates the reference levels of the glutathione peroxidase 3. Additional information is available in the eAppendix in Supplement 2.

The measurement of selenium blood levels in a subgroup of patients showed a clear separation of treatment groups and selenium levels were significantly higher in the treatment group over the observation period when compared with the placebo group (Figure 3A).^20^ Selenite supplementation successfully raised glutathione peroxidase 3 activity in the majority of the treatment group above the reference range of 250 U/L from postoperative day 2. However, glutathione peroxidase 3 activity did not show a significant difference between the treatment groups over time (Figure 3B).

## Discussion

In this rigorous, international trial of high-risk cardiac surgical patients undergoing complex procedures, high-dose selenium supplementation compared with placebo did not increase the number of days alive and without persistent organ dysfunction over the 30 days after cardiac surgery. No differences between groups were found for 30-day mortality, 6-month mortality, ICU length of stay, hospital readmission, daily rates of persistent organ dysfunction or frequencies of life-supportive therapies, or any other key secondary outcomes. Additional secondary outcomes demonstrated that fewer patients had a readmission to an ICU in the selenium group compared with the placebo group. Yet, these findings favoring high-dose selenium administration should be interpreted with caution and as hypothesis generating only as there was no adjustment made for multiple tests of significance and this finding could be a type I error.

Observational studies demonstrate that myocardial ischemia, reperfusion time, and duration of CPB are associated with a decrease of intraoperative selenium levels^8,9^ and that a prolonged CPB duration is a significant risk factor for postoperative complications.^17^

Preliminary evidence received from smaller studies^12,13,14^ provided mixed results on whether intravenous selenium supplementation was beneficial. Some studies demonstrating no effects, whereas others demonstrated significantly less vasoactive support, less myocardial injury, and significantly shortened hospital length of stay in patients receiving selenium. A potential explanation for these divergent findings on efficacy is that trials with nonsignificant effects used single-dose selenium at a lower concentration compared with trials that used a higher dose given multiple times. The potential discrepancies in results may be related to the selenium supplementation strategy. However, our results do not support the hypothesis that high-dose daily intravenous selenium improve major clinical outcomes in high-risk cardiac surgery patients.

There are several potential reasons for our neutral trial result, which differs from previous studies. Notably, our trial was the first and only multinational and multicenter study with an adequate sample size to determine if differences existed, to our knowledge. Studies with lower statistical power reporting significant treatment effects are less likely to be true positive and overestimate the size of the effect.^21^ Although we demonstrate an increase in selenium levels in the selenium group relative to the placebo group, normalization of circulating levels alone may not alter the cardiac surgery–related inflammatory response to oxidative stress. The activity of glutathione peroxidase, which is capable of neutralizing reactive oxygen and nitrogen species, did not differ between the trial groups indicating inadequate transcription of selenoproteins and thus a poor biological response to high-dose selenium. Patients may not have been deficient enough, as measured by their plasma selenium levels, as previous studies demonstrated best correlations between selenium and glutathione peroxidase at lower levels.^22^ Further, systemic inflammatory response-related cytokines are known to decrease expression of selenoproteins or secretion, which may limit the effect of high-dose selenium on urgent acutely diseased high-risk cardiac surgery cases.^23^ Therefore, the administration of selenium immediately before surgery may have been too late to provide intraoperative benefit in our trial as a significant increase in the antioxidant glutathione peroxidase can take 72 hours.^9,24^ The efficacy of a preoperative selenium supplementation strategy initiated 2 weeks prior to surgery showed clinical benefits and raises the hypothesis that the early introduction of selenium prior to cardiac surgery may be of benefit.^11^

Furthermore, recent innovations in surgical myocardial preservation techniques, such as combined antegrade and retrograde myocardial perfusion alone during bypass, are associated with smaller perioperative myocardial injury, which makes it challenging to demonstrate additional clinical benefits.^25^ Thus, speculatively, optimization of surgical techniques may have led to a progressively smaller periprocedural injury and postoperative complications in trial participants. Improvement in cardiac surgery management may diminish the potential benefits of any additional organ protective strategy.^12,13,14^ Our a priori subgroup analysis designed to identify these higher-risk subpopulations did not demonstrate any significant treatment benefit. While frail patients tended to favor selenium, patients with urgent procedures or patients with prolonged CPB time showed a trend favoring placebo, which is in contrast to our a priori hypothesis. There exist currently no surrogate markers to help identify patients who are sick but not too sick to ultimately benefit from the intervention and may provide explanations for the observed findings.^26^ More personalized approaches based on the patient’s illness severity and/or or inflammatory status seem to represent future goals for successful interventions. We further postulate that more strategic approaches for identification of patients at higher risk for complicated postoperative courses and/or in combination with earlier introduction of selenium may translate into improved clinical outcomes.

### Limitations and Strengths

One limitation of the trial is the use of the EuroSCORE II as part of the inclusion criteria to identify patients at increased risk for postoperative complications. The inaccuracy of the EuroSCORE II for cardiac surgery preoperative risk assessment is demonstrated in the discordance between the observed and predicted EuroSCORE II.^27^ Although the elevated EuroSCORE II closely correlated with the observed CPB and total operation time, indicating increased risk, this did not translate into clinically meaningful effects and the majority of enrolled patients had a rather short ICU length of stay. Future trials therefore need to consider how to identify and enroll patients at higher risk.

A second limitation is that our population is predominantly male. Biosynthesis of selenoenzymes and selenoproteins is sex specific in a dose-dependent manner.^28^ Further, our trial participants are predominately White, which may limit the generalizability of the findings to populations of other races and ethnicities. Selenium measurements differ according to ethnicity with Black individuals having the lowest selenium levels.^29,30^ In addition, the low enrollment of female individuals, which is consistent with other cardiovascular trials, makes it difficult to understand and address the implications of potential sex-specific responses.^31^

The strengths of this study include its robust scientific methods and high-fidelity implementation, the randomized and blinded design, rigorous determination of selenium laboratory analyses, and intent-to-treat analysis, all of which augment the internal validity of the trial. The high rate of adherence to trial interventions, large number of patients, and enrollment in ICUs in Canada and Germany underline the high degree of precision and external validity.

## Conclusions

In conclusion, the results of our trial in high-risk cardiac surgery patients show that perioperative high-dose selenium administration is safe but did not impact postoperative organ dysfunctions or mortality following surgery (eFigure 3 in Supplement 2).

## Notes

Supplement 1. Trial protocol and statistical analysis plan.

Supplement 2. eMethods. eFigure 1. Intervention Scheme eFigure 2. Six-month Kaplan-Meier Survival Curve eFigure 3. Product-Limit Survival Estimates eAppendix. Supplemental Data to Figure 3A and 3B eTable 1. Compliance with Study Investigational Product eTable 2. Protocol Violations and Concomitant Administration of Antioxidants or Corticosteroids eTable 3. Components of POD by Day eTable 4. Duration of PODs Components eTable 5. Hospital Acquired Infections eTable 6. Serious Adverse Events eReferences

Supplement 3. Nonauthor Collaborators. The SUSTAIN CSX Study Collaborators

Supplement 4. Data Sharing Statement
