# Supplementary material for: Effect of High-Dose Selenium on Postoperative Organ Dysfunction and Mortality in Cardiac Surgery Patients: The SUSTAIN CSX Randomized Clinical Trial
Source: JAMA Surg. 2023 Jan 11;158(3):235–44. doi: 10.1001/jamasurg.2022.6855 (PMC9857635; doi:10.1001/jamasurg.2022.6855)
Supplement: Supplement 1. — Trial protocol and statistical analysis plan. [file jamasurg-e226855-s001.pdf]

SodiUm SeleniTe Adminstration IN Cardiac Surgery (SUSTAIN CSX®-trial). A multicenter, randomized controlled trial of high dose sodium-selenite administration in high risk cardiac surgical patients

Clinicaltrials.gov #NCT02002247

Key words describing this protocol: Intensive care unit, critically ill, cardiac surgical procedures, organ dysfunctions, selenium, parenteral nutrition, knowledge creation, randomized trial.

Correspondence to:
Dr. Daren Heyland
Angada4
Kingston General Hospital
Kingston, ON K7L 2V7
Tel: 613-549-6666 x6241
Fax: 613-548-2428
Email: dkh2@queensu.ca

THE NEED FOR A TRIAL

1.1 What is the problem to be addressed?

A significant number of patients require cardiac surgery for the management of their heart disease. In addition to death, major morbidity from organ failure remains all too frequent following open heart surgery, particularly in patients at high-risk for complications and poor clinical outcomes. Such patients require life-sustaining therapies while their organs recover. Especially in older patients, the serious life-threatening complications may negate any benefit from correction of cardiac disease. These complications are in part due to inflammation and oxidative stress caused by the surgery itself.

Selenium is a trace element that is important for many of the body's regulatory and metabolic functions, especially during times of stress. Members of our study team have noted a perioperative depression of circulating selenium levels with cardiac surgery potentially leading to an insufficient capacity to deal with the stress of surgery. In a non-randomized interventional trial, our colleagues have shown that high-dose selenium supplementation was effective in preventing this decrease in intraoperative circulating selenium levels and that clinical outcomes were superior in this supplemented group compared to a historical control group. This led us to develop a protocol to test the effectiveness of high-dose selenium supplementation in a randomized trial in patients undergoing high-risk cardiac surgery. We recently successfully completed a multi-center, binational pilot RCT. The purpose of this protocol is to outline the rationale and methodology of a large-scale trial. We propose to conduct a randomized, placebo-controlled, double-blind, multicenter study of 1400 patients at 20 or more cardiac surgical centres. Patients will be randomized to receive either a daily perioperative high-dose sodium-selenite administration or placebo until the 10th postoperative day (maximum), or death or discharge from ICU. The primary outcome for this trial will be number of days alive and free of life-sustaining therapy within the first 30 days after surgery. If our hypothesis is proven true, and this simple, inexpensive nutrient reduces complications and improves recovery of patients undergoing cardiac surgery, we have the potential to dramatically change clinical practice and improve health outcomes for these heart patients.

1.2 What are the principal research questions to be addressed?

Primary Research Question:

What is the effect of high-dose selenium supplementation on number of days alive and free of life-sustaining therapy within the first 30 after surgery in high-risk cardiac surgery patients?

Secondary Research Questions:

What is the effect of high-dose selenium supplementation on the occurrence of infectious complications, major postoperative organ dysfunctions, length of stay parameters, mortality and 6 month health related-quality of life (HRQoL).

1.3. Why is the trial needed now?

1.3.1 Background Rationale:

Inflammation and cardiac surgery

Cardiac surgery is performed annually in approximately one million patients worldwide. By 2025, the demand for cardiac surgery is expected to increase on the basis of population growth and aging, if current healthcare use and service delivery patterns continue.[1] Despite substantial procedural advances, open heart surgery remains associated with disconcerting complication rates (15-20%) and mortality rates (3-4%).[2],[3] These rates may further increase as cardiac surgery is increasingly being performed on a patient population that is older and

presenting with an increasing number of comorbid conditions and complex coronary lesions.2 Three principal pathophysiological mechanisms have been identified that account for the majority of these systemic complications: ischaemia, reperfusion injury and inflammation.[4] Patients undergoing cardiac surgery with cardiopulmonary bypass (CPB) are exposed to various ischaemic stimuli, resulting from global ischaemic cardioplegic arrest of the heart and/or from embolic events. Reperfusion of the myocardium by surgical revascularisation and termination of cardioplegic arrest evokes oxidative stress[5] and triggers an intense inflammatory response associated with endothelial dysfunction, microvascular thrombosis, immune dysfunction, and eventually the potential for injury of virtually all vital organs, including heart, lungs, brain, kidneys and intestines (See Figure 1 in Appendix 1).[6,7]

Thus understanding the underlying mechanisms of harm from CPB results in strategies that potentially attenuate this stress response, reduce organ failure and improve health outcomes. There are both pharmacologic (e.g. perioperative glucocorticoid administration) and non-pharmacologic (e.g. off-pump) strategies that work on different aspects of this pathophysiological mechanism. Recent evidence on corticosteroids provides conflicting evidence that attenuating the inflammatory response may result in clinical benefit. The Dexamethasone for Cardiac Surgery trial recruited 4494 patients requiring CPB at 8 hospitals in the Netherlands and randomized them to a single intraoperative 1mg/kg dose of dexamethasone or placebo.[8] The primary outcome of the study was a composite of death, myocardial infarction, stroke, renal failure, or respiratory failure within 30 days. Overall, 7% of patients reached the primary endpoint in the dexamethasone arm versus 8.5% in the placebo group (RR 0.83, 95% CI 0.67 to 1.01, p=0.07). Furthermore, in a pre-specified subgroup analysis, the trial identified a statistically significant benefit in the composite primary end point for patients with a EuroSCORE of 5 or higher (RR 0.77, 0.61 to 0.98). While the primary outcome did not reach statistical significance, the results remain very important and support our hypothesis that attenuating inflammation may be beneficial, especially in high-risk patients. However, the Steroids In cardRiac Surgery (SIRS) Trial does not provide support for this hypothesis.[9] In the SIRS trial, 7504 high risk cardiac surgical patients (EuroSCORE ≥ 6) undergoing CPB at over 80 international sites were randomized to placebo or pulse dose methylprednisolone (two intravenous doses of 250 mg each, one during anesthetic induction and the other on CPB initiation).[10] The results suggest that perioperative administration of pulse dose methylprednisolone did not appear did not reduce the risk of death at 30 days (154 [4%] vs 177 [5%] patients; relative risk [RR] 0·87, 95% CI 0·70–1·07, p=0·19) or the risk of death or major morbidity (909 [24%] vs 885 [24%]; RR 1·03, 95% CI 0·95–1·11, p=0·52). Nevertheless, in this protocol, we are evaluating selenium, which does not act solely as an anti-inflammatory agent through immunosuppressive properties. Unlike steroids or other potential therapies, selenium may act at each step in the cascading series of events from stimulus to organ injury, as illustrated in Figure 1B in the Appendix. It is capable to modulate the inflammatory response through its antioxidant properties and provides organo-protective properties during myocardial ischemia/reperfusion injury (the underlying mechanisms are summarized in Figure 1B (Appendix). Selenium`s beneficial effect on the immune function[11] and its well established antioxidant properties[12] [4, 5] may render this trace element an attractive complementary option in support of high-risk cardiac surgery patients. Furthermore selenium will be supplemented through the entire perioperative period (up until 10 days), not just 2 doses of potential disease modifying therapies, to support the patient during the vulnerable postoperative period, where they are prone to secondary injuries from different directions.

The Role of Oxidative Stress

Ischemia-reperfusion related oxidative stress with the release of cytotoxic reactive oxygen (ROS) and nitrogen species (RNOS) is increasingly being recognized as a major factor contributing to the development of organ failure resulting in a prolonged stay in intensive care unit and increased mortality.[13] ROS and RNOS modulate cell signalling, proliferation, apoptosis, and cell protection. ROS and RNOS are also capable of degrading proteins, polysaccharides, nucleic acids and polyunsaturated fatty acids resulting in cellular damage and mitochondrial dysfunction.[14] Moreover, ROS / RNOS may trigger the release of cytokines from immune cells, activate inflammatory cascades, and increase the expression of adhesion molecules.[15] Thus, inflammation and tissue injury result in the accumulation of granulocytes in organs that lead to increased generation of ROS, which further perpetuates or amplifies the inflammatory response and subsequent tissue injury.[16]

In mammals, a sophisticated endogenous defense system protects tissues from oxidative stress. Several enzymes such as catalase, superoxide dismutase and glutathione peroxidase (GPx) are specifically designed to neutralize reactive oxygen species.[17] For these antioxidant (AOX) enzymes, the trace elements selenium, zinc and copper serve as essential co-factors. Particular interest in selenium has arisen as it is involved in multiple steps of intracellular AOX defense[18,19] and thus can neutralize both reactive oxygen and nitrogen species.[17] **Selenium is considered the cornerstone of antioxidant defense mechanism and may be one of the most important antioxidants**.[20] When incorporated into the various selenoenzymes, selenium influences the inflammatory signalling pathways that modulate ROS by inhibiting nuclear factor-kappa b (NF-kB) cascade, resulting in a suppressed production of interleukins and tumor necrosis factor alpha (TNFα).[19] Furthermore, circulating selenium levels have been previously shown to correlate with activity of glutathione-peroxidase and other selenoenzymes in various clinical settings.[21] In addition, selenium is known to affect both the cell-mediated and the humoral immune defense mechanisms and depressed selenium levels are associated with reduction of natural killer cells.[22,23] Considering the clinical setting of inflammation and myocardial ischemia/reperfusion, there is an impressive body of evidence demonstrating selenium`s organo- and cardioprotective properties, which are of particular relevance in cardiac surgery patients.[24]

Selenium and Critical Illness

A depression and redistribution of selenium and hence an insufficient endogenous AOX capacity has been repeatedly observed in critically ill patients with a systemic inflammatory response and/or multi-organ dysfunction and has shown to be associated with the severity of illness, a progression of organ failure and ultimately with mortality.[25,26,27,28] The majority of critically ill patients exhibited low plasma selenium levels that correlated inversely with the severity of the systemic inflammatory response syndrome (SIRS) and is associated with worse clinical outcome.[26]

Members of our study team have extensive experience with selenium studies in non-cardiac surgical but critically ill patients. Manzanares et al[29] evaluated the pharmacokinetic and pharmacodynamic profiles of selenium in a prospective, randomized, pilot study in 20 critically ill adults patients. They compared 2 doses of IV selenium: 1200 µg loading dose over 2 h and thereafter 800 µg/d as a continuous intravenous infusion for 10 d or 2000 µg loading bolus over 2 h and thereafter 1600 ug/d as a continuous infusion for 10 days. The maximum selenium concentration and the maximum GPx-3 activity were in the physiological range in both groups however GPx-3 activity was higher in the group that received the highest dose (see Figure 2 in Appendix 1). More recently, in a Phase II study, Manzanares and colleagues[28] safely administered an initial bolus of 2,000 µg selenium over 2 h, and thereafter 1,600 µg/day selenium as a 24 hr continuous infusion daily for 10 days and compared to placebo. Organ dysfunction, evaluated by Sequential Organ Failure Assessment scores decreased significantly

in the selenium group (1.3 ± 1.2 versus 4.6 ± 2.0, P=0.0001) compared to placebo. Furthermore, the incidence of early ventilator-associated pneumonia was significantly lower in the intervention group (6.7% versus 37.5%, P=0.04), and hospital-acquired pneumonia was lower after ICU discharge (P=0.03). Finally, the authors again demonstrated that selenium pharmacodynamics in critically ill patients showed an increase in GPx-3 activity from day 1 through day 3 to a maximum at day 7, compared to the previously published physiological range for non-SIRS and healthy subjects.

In addition, Heyland and colleagues recently completed a large-scale randomized trial evaluating selenium and other antioxidants in critically ill patients with multi-organ failure.[30] In a blinded, 2 x 2 factorial trial (glutamine was the other factor) involving 40 ICUs in Canada, the United States, and Europe, 1223 critically ill, mechanically ventilated adult patients with multi-organ failure were randomized to selenium, 500 µg intravenously (selenase®, biosyn), and the following vitamins and minerals administered enterally: selenium 300 µg, zinc 20 mg, beta carotene 10 mg, vitamin E 500 mg, and vitamin C 1500 mg, or placebo. No bolus administration of selenium was used in this study. Study supplements were administered separately from standard nutrition and provided continuously for a maximum of up to 28 days. The primary outcome was 28-day mortality. We could not demonstrate any favourable effect of selenium and the other antioxidants (or glutamine) in this study. However, in a secondary analysis, we conducted several post hoc subgroup analyses to identify any potentially important subgroup effects. It appears that patients with renal dysfunction upon study enrolment experienced increased harm from both glutamine and antioxidant supplementation. In addition, in a lab sub-study in a subset of patients, despite supplementation with selenium, the median selenium levels remained within normal ranges in both groups at all time points (see Figure 3 in Appendix). We may have utilized an insufficient selenium dose, or an ineffective dosing schedule, as achieving a higher than normal level of selenium in the blood may be associated with the best outcome[31] and an initial 'bolus' administration of selenium might have been more effective than the continuous administration we used.

More recently, another large-scale trial of selenium supplementation has recently been completed. The Sodium Selenite and Procalcitonin Guided Antimicrobial Therapy in Severe Sepsis (SISPCT study, Clinical Trials-ID NCT00832039), a multi-center RCT in Germany recruited 1180 patients with severe sepsis or septic shock from 33 German ICUs. Patients were randomized to receive an initial intravenous loading dose of 1000 µg sodium selenite followed by continuous intravenous infusion of 1000 µg sodium selenite daily until discharge from the ICU, but not longer than 21 days, or placebo. The analysis of the results showed that there was no statistically significant difference in 28-day mortality between the selenium (152/543 [28.3%, 95 % CI 24.5% to 32.3%]) and the placebo-group (137/538 [25.5%, 95 % CI 21.8% to 29.4%], p=0.302) (Personal Communication: Frank Bloos, Principal Investigator). Importantly for our trial, the SISPCT trial did not demonstrate any increased harm in patients with renal failure.

To summarize the overall treatment effect in ICU patients (non-cardiac surgery), we recently conducted a systematic review of the literature to evaluate the effect of high-dose intravenous selenium (including the above mentioned SISPCT trial). After aggregating 21 RCTs involving 4132 patients, we found that selenium supplementation had no effect on mortality (RR 0.99, 95 %, CI 0.90, 1.08, P= 0.79, heterogeneity I^2=0%). In addition, when the results of 10 trials that reported on infections were statistically aggregated, parenteral selenium was not associated with a reduction in infections (RR 0.95, 95 % CI 0.88, 1.02, P=0.15, I^2=0%). However, after aggregating 4 studies that reported on ventilator associated pneumonia (VAP) we found that selenium was associated with a significant reduction in the incidence of VAP (RR 0.72, 95 % CI 0.58, 0.89, P=0.002, I^2=0%). Finally, we did not find any effect on ICU length of stay (LOS), hospital LOS, ventilator days or incidence of renal insufficiency or failure.

We postulate that it may be difficult to see a signal across heterogeneous trials in heterogeneous patients in the ICU setting and it may be easier to detect a signal in cardiac surgery patients, a more homogenous patient population.

Selenium and Cardiac Surgery

Our European colleagues previously demonstrated that the majority of patients undergoing cardiac surgery exhibited a significant selenium deficiency prior to CPB, which was further aggravated with increasing CPB time and continued to decrease postoperatively, when compared to selenium levels from a healthy population.[32] A total of 60 patients were enrolled in this prospective observational study. Seventeen patients recovered uneventfully whereas 31 patients developed a single organ failure and 12 patients a multi-organ failure. The perioperative time course of the development of the selenium deficiency was measured and we found the decrease in selenium to have a significant predictive accuracy for subsequent development of multi-organ failure (see Figure 4A, Appendix 1), indicating a significant role of circulating selenium levels in the clinical setting of cardiac surgery. Of note, the observed intraoperative decrease was most pronounced in patients undergoing on pump cardiac surgery when compared to patients with off-pump procedures, indicating myocardial ischemia/reperfusion or the duration of CPB to represent the most important determinant for decrease of selenium levels.[33]

As a next step, members of our study team completed a single-center, open label trial to determine the safety and pharmacokinetics of high dose selenium supplementation in cardiac surgical patients.[34] In 100 patients undergoing cardiac surgery, they administered 2000µg IV after induction of anesthesia and 1000 µg on the following day and 1000 µg daily thereafter in ICU and demonstrated that these doses were effective in preventing the intraoperative decrease of circulating selenium levels that was previously reported by our group.[34] When comparing selenium treated patients with a historical control group of cardiac surgical patients, the treatment group had less organ dysfunction on postoperative day 1. However, the loading-dose was apparently not high enough to prevent the fall in circulating selenium levels to baseline levels at the first postoperative day (Figure 4B). For this reason, we propose to add a second loading dose on admission to ICU to compensate for this.

1.4 Systematic Reviews of the Literature

Few randomized trials of perioperative selenium supplementation in cardiac surgery have been performed to date. To provide an overview about the completed and on-going trials investigating the significance of selenium supplementation in cardiac surgery, we have summarized the major results in Table 1. Of most relevance, Haberthür *et al* recently completed a randomized controlled and demonstrated the safety of high-dose selenium supplementation (4000µg as bolus prior to surgery) in general cardiac surgery patients (Haberthür *et al* 2015 in revision at Crit Care Med). Notably the selenium supplementation prevented the postoperative drop of selenium blood levels and significantly shortened the ICU and hospital length of stay by 8.2 hours and 1.9 days, respectively (p=0.02). However, no effect was observed on the postoperative SOFA-score, its related organ specific scores or mortality compared to placebo in this cohort of general (low-risk) cardiac surgery patients. This trial provides strong evidence for the safety of high-dose selenium in cardiac surgery patients, including patients with renal failure. However, they failed to demonstrate a convincing benefit because of their inclusion of low-risk patients. The focus of the SUSTAIN protocol is on high-risk cardiac surgical patients.

To date, there have been several other studies of antioxidant strategies (not just Selenium) in cardiac surgery that provide some evidence for our treatment approach.7 In one study, a cocktail of antioxidants (coenzyme Q10, magnesium, lipoic acid, omega-3 fatty acids and selenium) was compared to placebo.[35] This trial of 'metabolic support' in 117 patients did

demonstrate that the intervention group has improved markers of oxidative stress, less myocardial injury, and reduced length of hospital stay by 1.2 days. By no means is this a definitive study but it does support our hypothesis that key nutrients can ameliorate oxidative stress and improve the outcomes of cardiac surgical patients. N-acetylcysteine (NAC), a precursor to glutathione, is another drug with antioxidant effects that has been extensively studied in cardiac surgery. Some studies have shown reduced markers of inflammation and oxidative stress, reduced myocardial injury and improved cardiac performance, and reduced ICU and hospital length of stay.7[,36,37] However, systematic reviews of NAC do not show conclusive benefit.[38,39,40]

In summary, the pathophysiological rationale, the clinical trial data we have systematically reviewed, and the results of our open label pilot study clearly justify moving forward with this randomized controlled trial in a selected group of high-risk cardiac surgical patients in which selenium will be delivered pre-emptively to maintain the AOX-capacity prior to the onset of oxidative stress. Our prior work demonstrates our capacity to do research in this area- we have experts in selenium, experts in clinical trials, and partnerships with cardiac surgical centres with a record of participating in multi-centre trials. Our pilot study demonstrates the feasibility of the proposed protocol.

Pilot RCT of This Protocol

In January of 2014, we initiated a multi-center, pilot trial of this exact protocol, funded by the Canadian Institute of Health research (CHIR), which is currently conducted at 3 German and 3 Canadian cardiac surgical centers. The purpose of the pilot was to uncover problems regarding recruitment of patients, adherence with the study protocol and any contaminations. We have obtained the necessary regulatory approvals in both countries and ethical approvals from all participating sites. To date, we have screened 5.6 patients/site/month and randomized 1.5 patients/site/month. More than 94% of prescribed study medication was received, we have received no major protocol violations, and no patient in the control group has received open-label treatment. At this point, we conclude our study protocol is feasible and now propose to conduct a randomized, placebo-controlled, double blind, multicenter study. We hypothesize that the therapeutic strategy tested in this randomized trial may contribute to a decrease of postoperative morbidity and mortality.

1.5 How will the results of this trial be used?

If the definitive trial is positive, we will use these results to inform the clinical practice of cardiac surgery around the world. These societies will play key roles in future knowledge translation activities. As it relates to critical care nutrition practice in general, we have a long history of practice-changing initiatives that can be tailored or adapted for use in local cardiovascular ICUs. We have a process of synthesizing (in the form of evidence-based clinical practice guidelines) and disseminating best practice ideas (in the form of web-based repository of tools and information [see www.criticalcarenutrition.com]) and have conducted several large cluster RCTs to introduce system-changing practices in ICUs in North America and Europe.[41,42,43]

1.6 Describe any risks to the safety of the participants involved in the trial.

Given the data of the recently published open-label trial, the chosen dose and method of administration of sodium-selenite appeared to be safe. By the 10th day of treatment, selenium levels in patients did not exceed the reference values documented for Germany or the U.S (Figure 4B, Appendix 1).[44] Notably, the observed levels stayed multiple times below the serum concentrations for which the onset of selenium poisoning has been reported in the literature, i.e., >534µg/l.[45,46] During the entire observation period, the rate of adverse events was not

increased by selenium administration. Our results are in line with recent reports on the safety of high dose selenium administration in critically ill patients. In this work, done by one of our co investigators, Dr. William Manzanares, the frequency of adverse events did not differ when compared with patients receiving lower doses of selenium or placebo.[28,29]

Based on the findings of the REDOXS study, there was some concern about the safety of antioxidants in critically ill patients with multiorgan failure including renal failure. However, subsequent large-scale RCTs of monotherapy of selenium have not demonstrated any increased risk of harm with selenium in critically ill septic patients with multi-organ failure (Bloos) nor in cardiac surgery patients (Harbethur). These clinical findings are in line with the fact that selenium is mainly excreted from the body via the faeces or exhaled air (in form of $(CH_3)_2Se$) or after further methyltransferation as $(CH_3)_3Se^+$ / selenosugar via the urine.[47,48] Reviewing the literature, no reports exists about an accumulation of selenium in the kidney.[12] Despite intensive investigations, no potential confounding factors have been detected, which may negatively influence the overall excretion of selenium, which may be due to the different excretion ways in the human body.

2. THE PROPOSED TRIAL

2.1 What is the proposed trial design?

We are proposing a randomized, double-blind, placebo controlled trial of 1400 patients in more than 20 centers in Canada, United States and Germany

2.2 What are the planned trial interventions?

Patients will receive daily perioperative treatment of either high-dose sodium-selenite administration or placebo. As with our formerly mentioned preliminary studies, all patients will receive an IV bolus of 2000µg selenium (equals to 40ml prepared solution) or the same volume of normal saline (placebo) within 30min after induction of anesthesia via the central venous catheter. By bolus, we mean the study drug should be administered over 30 to 120 minutes. This bolus dose should be completed *prior* to commencing cardiopulmonary bypass. After termination of surgery, immediately after admission to the ICU, all patients will receive a second bolus of 2000µg selenium or placebo accordingly. This additional post-operative bolus is in response to the post-operative drop in selenium levels observed on day 1 in our open label trial (Figure 2).[34] This post-operative drop may be due to bleeding or transfusions that frequently occur in the first 24 hours of surgery in patients with prolonged CPB. Then, as per our open label trial, on every further morning (8:00 am) during ICU-stay, patients will receive a bolus of 1000µg selenium (equals to 20ml prepared solution) or placebo via central or peripheral venous access over 30 to 120 minutes. The daily administration of study solution will continue until death, discharge from ICU to the ward (treatment may continue in a step down or intermediate care unit), or for a maximum of 10 days. This dosing regimen was chosen according to efficacy, tolerability and safety that was confirmed in previous supplementation trials in patients with systemic inflammation and cardiac surgery.[29,34,40] The study supplement and placebo solution will be supplied by biosyn, an industry partner that manufactures intravenous selenium and will be provided in a way to maintain blinding.

Intervention scheme/ Trial flow

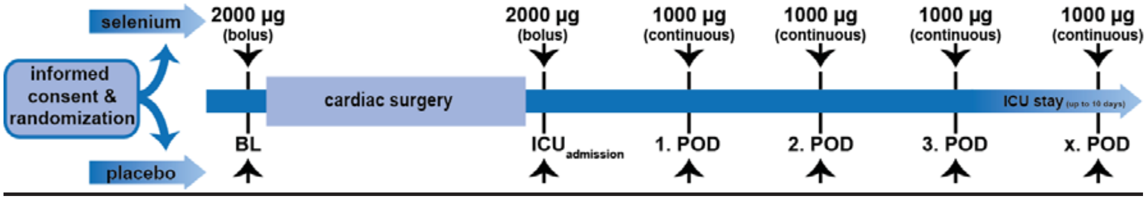

Legend: overall schema of SUSTAIN CSx trial

2.3 What are the proposed arrangements for allocating participants to trial groups?

At each participating center the local coordinating investigator will screen daily all cardiac surgical patients scheduled to undergo cardiac surgery in the near future or on the next day. A screening log will be kept at each site to determine the number of patients meeting the inclusion criteria, those truly eligible patients, those who consent and are randomized and reasons why potentially eligible patients did not get enrolled. Following a full explanation of the nature and purpose of the study, a written informed consent will be obtained from the patients participating in the study. At the time of enrolment into the study, patients will be randomized to receive either selenium or a matching placebo similar in appearance, consistency, volume, and smell so as to **blind patients, investigators and health care practitioners** as to the nature of the study medication. Patients will be consecutively randomized by a web-based randomization system (concealed and blinded) developed by the Clinical Evaluation Research Unit at the Kingston General Hospital and randomization will be stratified according to centre. Randomization will be based on the method of permutated blocks of undisclosed random size and stratified by centre.

2.4 What are the proposed methods for protecting against other sources of bias?

At the time of enrolment into the study, researchers will be blinded to the next treatment assignment (guarding against *selection bias*). Patients will be randomized to receive either selenium or a matching placebo similar in appearance (consistency, volume, and smell so as to **blind patients, investigators and health care practitioners** as to the nature of the study medication (guarding against *performance and detection bias*)). Consistent with the intention-to-treat principle, all randomized patients will be included in the analysis. Large numbers of patients lost to follow-up would threaten the validity of this trial. As explained in section 2.14, given that the study occurs in hospital and the majority of the outcomes, including the primary outcome, are assessed in hospital, we anticipate no lost to follow-up. To guard against attrition bias, we will take the following proactive strategies shown to enhance retention[49,50]: 1) We will obtain the contact information of an alternate contact to contact the patient and, if the patient is not able to provide information, to obtain the most important patient-centered physical function on SF-36 from the alternate contact. 2) The RC will make contact with the patient and alternate contact at the time of ICU discharge. 3) Respondents will be notified of upcoming interviews by means of mailed-out reminders. 4) Contact information from additional alternate contacts at time of enrollment will be recorded. 5) We will obtain survival status of all patients lost to follow up from public registries and on-line sources.

It is important to consider the influence of other treatments on study outcomes. Given the multicenter, multinational nature of this study, it will be impossible to standardize all co-interventions. However, we will capture key process of care issues in our data collection strategies (intravenous steroids, cell-salvage practices, etc. as further outlined in Appendix 2).

2.5 What are the planned Inclusion/Exclusion criteria?

We define enrolment criteria to identify the patients who are likely to suffer from inflammation and oxidative stress and thus benefit most from the therapeutic interventions tested in this study. We aim only to enroll:

Inclusion Criteria

Adult patients (≥18 years of age) scheduled to undergo elective or urgent cardiac surgery with the use of cardiopulmonary bypass (CPB) and cardioplegic arrest that exhibit a high perioperative risk profile as defined by the presence of one or more of the following:
a) Planned valve surgery combined with CABG or multiple valve replacement/repair surgeries or combined cardiac surgical procedures involving the thoracic aorta; OR
b) Any cardiac surgery with a high perioperative risk profile, defined as a predicted operative mortality of ≥ 5% (EuroSCORE II).8[,51]

We justify focusing on high-risk patients described above as patients that are characterized by this profile have been recently shown to experience an excessive systemic inflammatory response with most pronounced decrease of selenium during surgery.[32,34,52] The DECS trial also suggested benefit of inflammatory suppression in this subgroup.8 Furthermore we have demonstrated that postoperative selenium blood levels were inversely correlated with duration of CPB, i.e., the longer the surgical procedure, the more pronounced the postoperative decrease in circulating selenium levels ($r=-0.121$; $p<0.05$).[32,52] In addition, the results of our collaborators and those from a recently published study revealed that the preoperative assessed EuroSCORE inversely correlated to the postoperatively measured selenium levels ($r=-0.312$; $p<0.01$), indicating that this is the group of people who are most likely to benefit from perioperative selenium supplementation.[53] These same high-risk criteria identify a patient population that is likely to experience a prolonged ICU course,[54] which will offer some statistical efficiencies given the higher rate of organ dysfunction and need for prolonged administration of life-sustaining therapies.

Exclusion Criteria

We will exclude patients who meet any of the following criteria:
1) Isolated procedures (CABG only or valve only)
2) Known hypersensitivity to sodium-selenite or to any of the constituents of the solution.
3) Renal failure requiring dialysis at the point of screening.
4) Chronic liver disease as evidenced by a pre-operative total bilirubin >2 mg/dl or 34 μmol/L
5) Disabling neuropsychiatric disorders (severe dementia, severe Alzheimer's disease, advanced Parkinson's disease).
6) Pregnancy or lactation period.
7) Simultaneous participation in another clinical trial of an experimental therapy (co-enrolment acceptable in observational studies or randomized trials of existing therapies if permitted by both steering committees and local ethics boards).
8) Patients undergoing heart transplantation or preoperative planned LVAD insertion or complex congenital heart surgery.
9) Alternate contacts of investigators (required by German Regulatory Authorities).

Additional exclusion criteria for the functional outcome assessment sub-study:

1. Not ambulating independently prior to cardiac surgery because of neurological illness or lower extremity impairment (use of gait aid permitted).

These exclusion criteria will enable us to exclude patients who may be harmed by the study intervention (e.g. hypersensitivity to selenium) or will have atypical post-operative course

(patients undergoing heart transplantation). We will enroll patients with acute kidney injury or chronic renal insufficiency provided that they do not require dialysis and plan a sub-study to examine pharmaco-kinetic/pharmaco-dynamic response to high-dose selenium in this subset of patients (see Section 2.8 below). We will exclude patients on dialysis from this sub-study given the difficulties in interpreting the pharmco-kinetic data in this group compared to patients with renal insufficiency not requiring dialysis.

All patients who meet the eligibility criteria will then be approached for informed consent. Those patients (or their proxies) who do not provide informed consent will not be randomized into the study.

2.6 What is the proposed duration of treatment period?

The daily administration of study solution will continue until death, discharge from ICU to the ward (treatment may continue in a step down or intermediate care unit), or for a maximum of 10 days.

2.7 What is the proposed frequency and duration of follow up?

Enrolled patients will be followed daily while in the ICU. The hospital chart will be reviewed upon discharge to abstract all hospital related outcomes. To better understand the impact of study treatments on longer-term survival and health-related quality of life (HRQOL), we will follow surviving study patients for 6 months. At POD 30, 3 and 6 months post randomization, a trained research coordinator at each site will contact patients discharged from hospital to assess survival status, whether they have resumed normal activities, and administer the SF-36 and Barthel ADL over the phone (see Appendix 3 for details).

2.8 What are the proposed primary and secondary outcome measures? & 2.9 Measures at follow-up:

The selection of the primary outcome for large-scale trials in cardiac surgery, even among high-risk patients, is problematic.[55] Options include mortality, length of stay in the ICU, and composite endpoints. At the Ottawa Heart Institute, over the past 5 years, in-hospital mortality rates for both elective and emergent surgery were 3.1%. A total sample size of over 22,000 patients would be required to achieve 80% power to detect a 20% RRR reduction to 2.5% at a two-sided alpha=0.05. Furthermore, focusing on mortality alone misses the beneficial (or adverse) effect of treatments on morbidity and quality of life. Length of stay in ICU may be a candidate outcome but discharge practices are tremendously variable across units and are dependent on non-clinical factors such as availability of beds rendering this outcome less sensitive to detect a treatment effect. A standard composite endpoint of all-cause mortality, myocardial infarction, stroke, renal failure, and prolonged mechanical ventilation could be considered but the inclusion of stroke, particularly due to embolic origins, are not expected to be modified by selenium. Moreover, combining myocardial infarction defined by a biochemical change (troponin rise) with an event such as death challenges the validity and clinical significance of this composite endpoint.[56]

Although these "hard" measurements are undoubtedly important, they do not adequately capture patients' perspective after discharge from hospital.[57] Furthermore, interventions should not exclusively attempt to save lives or reduce the incidence of severe postoperative complications, but may also aim to improve postsurgical morbidity and quality of life. In contrast to immediate term outcomes, such as mortality alone or ICU length of stay, the knowledge of long-term survival of these patients could assist decision-making about further therapeutic strategies and uncover important treatment effects.[58] Therefore we propose to use "persistent organ dysfunction+death (POD+Death)" as a composite endpoint. We define POD

as the need for life-sustaining therapies (mechanical ventilation, vasopressor therapy, mechanical circulatory support, continuous renal replacement therapy, or intermittent hemodialysis). We have validated this endpoint in both critically ill and cardiac surgery patients and have shown that patients who develop PODS are at higher risk for subsequent death and long-term disability or lower quality of life compared to those who do not have POD.[59,60] In a similar study of cardiac surgery patients, Williams and colleagues have shown the that persistence of multiple organ failure identifies a subpopulation of surgical patients that have a high likelihood for death or poor physical function over the subsequent 2 years.[61] Moreover, given our biological model illustrated in Figure 1, PODS would be the most direct outcome and the most sensitive to detecting a treatment effect of selenium. Hence, we propose to use POD+death as the primary outcome calculated as PODS-free days within the first 30 days (as described below in the Statistical Section). The need for and use of life-sustaining treatments is causally related to post-operative inflammation and oxidative stress injury and has important clinical and economic consequences. The determination of POD uses readily available clinical parameters that will be easily discerned either prospectively or retrospectively. We focus on the use of interventions to support failing organs rather than the measurements of organ failure themselves because of the cost and complexity of following organ function over time, which requires specialized blood tests and results in high amounts of missing data.[59] In addition, traditional organ failure scoring systems (such as Sequential Organ Failure Assessment Score[62]) have not been validated in cardiac surgery patient populations and the presence of mechanical assist devices are not captured in their scores.[63] We acknowledge that this method of determining POD is subject to clinician influence, and that there will be variability across sites in how these life-sustaining treatments are used or withdrawn. However, it would be impractical to standardize the use of these treatments at all participating centers and we are stratifying by center to minimize the influence of this noise in our analysis.

Secondary outcomes are described in more detail in Appendix 2 and will include cardiovascular complications (e.g. arrhythmias, cardiac arrest, infarction), duration of mechanical ventilation, the incidence of postoperative delirium (assessed by CAM-ICU score[64]), length of stay in the ICU and hospital, re-admission rates, hospital-acquired infections (proven or suspected), and 30-day mortality. Additionally, we will contact patients by telephone at 3 and 6 months post randomization to assess long-term survival and health-related quality of life, like we did in our previous antioxidant trial in critically ill patients.[30] Finally, we will capture costs to the health care system and calculate incremental cost per quality adjusted life years (cost/QALYs).

Laboratory Evaluation

In the definitive study, the laboratory component will be optional. Those sites that elect to participate in the lab substudy will draw blood to assess the potential effects of supplementation on selenium levels, safety parameters and other mechanistic markers. Blood will be drawn at baseline (pre-treatment), after surgery (ICU admission), and then daily until day 10 (if still on treatment with selenium supplementation), unless the patient was discharged or died earlier. Whole blood levels of selenium (measured by atomic absorption spectroscopy to ensure determination of selenium independent from the compartment), selenoprotein P (Sel-P), antibodies against oxidized LDL, markers of inflammation (interleukin[IL]-6, IL-10, TNF alpha) and activity of glutathione-peroxidase (GPx) will be assessed to determine the efficacy of selenium supplementation in these patients. We will collect additional blood samples to conduct additional tests, as determined by the investigators that may help explain the role of selenium in this setting. Blood samples will be stored until final analysis of the study. Standard lab tests to detect myocardial ischemia (e.g Troponin), inflammation, and organ function

(creatinine, urea, bilirubin, and hemoglobin) will be done according to clinical routine and as clinically indicated.

Beside the evaluation of inflammation and oxidative stress, we aim to perform genotyping analysis to evaluate the effects of genotype on metabolism, selenium status and health outcomes. The relative ratio between two isoforms of Sel-P has been reported to be influenced by genotype with respect to two single nucleotid polymorphisms (SNPs) in the Sel-P gene, the effect of which was abrogated under conditions of selenium supplementation.[65] Since Sel-P is crucially involved in the systemic selenium transport (blood and tissue), we thus aim to evaluate the significance of underlying genotype on patients` response to supplementation and patients` outcome after surgery.[65,66] Furthermore we reveal new insights into the underlying genotype with respect to further selenoproteins.

2.10 Will health service research issues be addressed?

We will perform a cost utility analysis alongside this randomized controlled trial. The cost effectiveness of sodium-selenite administration compared to placebo will be assessed in terms of the incremental cost per quality adjusted life year (QALY) gained from the perspective of health care system. Analysis will incorporate data on resource use and patients utility values up to 6 months post sodium-selenite administration given the assumption that no long term differences in outcome are expected. Resource use will be assessed by data collected at the follow up interviews. Utility values would be derived from SF-36 using the algorithm proposed by Brazier et al.[67] QALYs will be estimated for each patient within the clinical trial using the total area under the curve method.[68] The incremental cost and QALY will be estimated using a regression analysis approach. Uncertainty in the analysis will be addressed by estimating 95% CIs using a non-parametric bootstrapping method.

2.11 What is the proposed sample size and what is the justification for the assumptions underlying the power calculation?

The distribution of the control arm was calculated based on a database of patients that underwent cardiac surgery during a 12 month follow up at Aachen University (n = 1127) and would met the inclusion criteria of the current study (n = 170). In our dataset, the mean (SD) POD free days was 23.2 (9.2); 4% of the patients died, 6% survived on life-sustaining therapy with 0 POD free days. We checked these numbers with data from the University of Ottawa Heart Institute (Canada), and numbers are consistent with the 4% 30-day mortality rate and the 6% not yet free from life-sustaining therapy by 30 days. We used simulation to estimate the power of applying the Wilcoxon rank-sum test to our primary outcome of PODs free days. The intervention arm was then generated by multiplying the control arm daily rate of liberation from life sustaining therapies by a fixed factor (hazard ratio) but assuming the same 4% mortality rate. The mean days on life-sustaining therapy was then subtracted from 30 to obtain the free days. 10.000 samples were simulated so the power estimate has more than a 95% chance of being accurate to within 1%. **Based on the simulation, we would require 700 patients per arm to achieve 90% power at a two-sided alpha=0.05 if the intervention caused as 20% relative increase in the daily rate of liberation from life-sustaining therapy but no change in mortality compared to the control arm.** Based on our Aachen data, such an effect size would result in an earlier liberation of life-sustaining therapies and mean increase of 1.5 POD free days (from 23.2 to 24.7 days). We believe such an effect size is plausible and is in line with minimally clinically important differences accepted in other recent major trials in the ICU setting.[9]

2.12 What is the planned recruitment rate? Over what time period will recruitment take place? What evidence is there that the planned recruitment rate is achievable?

Based on our recent pilot trial experience, we expect study start up activities to take between 6-8 months. In the pilot trial, we observed an enrolment rate of 2 patients/site/month. We plan to roll in the pilot trial and thus, to enrol 1320 patients at 20 sites, we need 33 months to enrol the total sample. We will add 6 months to enrolment time to allow for final follow up. The final 6 months will be used for analyzing data, and writing reports. In total, the trial will take approximately 4 years.

2.13 Are there likely to be any problems with compliance? On what evidence are the compliance figures based?

Study medications are administered to patients pre-, intra-, and post-operatively while in the operating room and ICU (for a maximum of 10 days). There are no side effects associated with administration of study medication. In the open-label trial conducted by our European colleagues, 96% of study doses of medication were received. The reasons for non compliance were unexpected intraoperative change of scheduled surgical procedure (off-pump technique and LVAD-implantation). In the pilot RCT, 96.5% of study doses were received. Reasons for non-compliance included unexpected intraoperative change of scheduled surgical procedure (off-pump technique), patient discharged that day from ICU and one patient refused further doses while waiting for transfer to ward bed. Accordingly, we expect good compliance with administration of the study medication.

2.14 What is the likely rate of loss to follow-up? On what evidence is the loss to follow-up rate based?

The primary outcome will be assessed in the hospital. We do not expect any lost to follow-up in assessing the ICU and hospital outcomes. We do plan to follow patients for long-term health related quality of life via telephone. So far in our pilot study, 3 patients have been lost to follow up at 6 months (4.3%). From our previous experience with other longitudinal studies, we expect anywhere from 10-15% lost to follow up rates for this outcome assessment.[30,84,85] As outlined in section 2.4, we have taken several proactive steps to minimize lost to follow-up for this secondary outcome.

2.15 How many centers will be involved?

The sites that participated in the pilot RCT will continue their involvement in the study. Additional sites participating in this trial are listed in the Table in Appendix 4. It is important to note that sites participating in the German component recently collaborated on another major multicenter trial of cardiac surgery patients published in the New England Journal of Medicine (RIPHeart-Study Group).[69]

2.16, 2.17, and 2.18: What is the proposed type and frequency of analyses and planned subgroup analyses?

As previously stated, the primary endpoint for this definitive study is the number of days alive and free of life-sustaining therapy within the first 30 after surgery. The 30 day time frame is commonly used in the intensive care literature, because there is virtually no loss to follow-up during this period and there are only about 5% of outlying patients who remain alive and dependent on life-sustaining therapy after (sometimes months after) 30 days. Patients who die within 30 days will be given a value of 0 free days. In total we expect no more than 10% of patients will have 0 free days due to death or remaining dependent on life-

sustaining therapy for 30 days. This outcome will have properties similar to "Ventilator Free Days" which has been widely accepted in critical care medicine and used in several recent major RCTs,[70,71,72] despite often having much higher rates of 0 free days. Free days will only be counted if they persist for at least 48 hours prior to re-application of life sustaining therapy and are not followed by death within 30 days. We propose to compare the primary outcome between arms by the Wilcoxon rank sum test. The daily proportion of patients alive and free of life sustaining therapy by arm over the first 30 days will be depicted graphically. The primary safety analysis will follow the intent-to-treat principle including all patients who received investigational product in the arm they were randomized to regardless of treatment compliance. However, we will also conduct a modified intention-to-treat analysis that excludes patients that received on dose of study medication but then did not undergo the planned surgery and consequently, did not continue on the randomized treatment. In addition, we plan a per-protocol analysis that further includes patients that stay more than 1 day (24 hrs) in ICU and experienced no IP-related protocol violations Based on our extensive experience with similar patient populations, we expect loss to follow-up in the first 30 days prior to death or ICU independence to be trivial.

Secondary binary outcomes such as cardiovascular complications, postoperative delirium, re-admissions, hospital-acquired infections and 30-day mortality will be compared between groups by a logistic mixed effects model with site as a random effect[73]. Length of ICU and hospital stay as well as long term survival will be described by a Kaplan-Meier curve and compared between arms using the Cox proportional hazards model with site as a random frailty to account for potential between site heterogeneity. Note that without the random site frailty, this method equates to the well-known log-rank test. Patients who die prior to discharge will be treated as if they were never discharged by being censored after the end of follow-up.

The SF-36, Barthel Index and frailty scale will be collected at baseline and at 30 days, 3 months and 6 months after randomization we will collect return to work data and repeat the Barthel Index and SF-36. The SF-36 physical and mental summary scales, the Barthel index and the frailty scale will be treated as continuous variables and analyzed using a linear mixed effect repeated measures analysis. This model will include site as a random effect and will allow for unstructured within patient correlation as estimated by restricted maximum likelihood. The focus of these secondary analyses will be primarily descriptive; the between group difference in the change from baseline to the various follow-up time points will be described as expected means with 95% confidence intervals. The pattern mixture approach for longitudinal data will be used to perform a sensitivity analysis under a range of assumptions for missing data and death.[74,75]

A priori, we expect that sicker patients with less reserve may benefit the most from selenium supplementation.[32,53] Thus, we plan to do a subgroup analysis comparing the treatment effect in patients who are frail (Clinical Frailty Scale $\geq$5)[76] vs. those who are not, in patients that undergo combined CABG and AVR vs. those that do not, and patients with a higher vs lower Euroscore (based on the median score) and longer vs. shorter CPB (based on median value). In support of these proposed analyses, there is an apparent decline in circulating selenium levels in the elderly in certain populations, which may occur independently of intake.[77,78] Given the potential differences in baseline selenium levels between North Americans and Europeans (due to selenium depletion in the soil in Europe),[79] we plan to compare the effect of selenium in the Canadian vs. German subpopulations. Treatment effects will be presented within subgroup and for the primary outcome we will use the aligned rank tests to formally test for a treatment by subgroup interaction.

As previously noted, we plan a sub-study in patients with renal dysfunction at baseline. After 40 such patients are enrolled, we will examine blood levels of selenium,

selenoprotein P and activity of glutathione peroxidase 3 (and other mechanistic markers). We will compare to other patients receiving active treatment to evaluate the response on selenium supplementation and to ensure that there is no accumulation of selenium in these patients. Study reporting will be in accordance with the CONSORT statement including a patient flow diagram and a descriptive by arm comparison of all important baseline characteristics.[80]

2.19 Has any Pilot study been carried out using this design?

As previously mentioned, we are conducting a multi-center pilot study approved and funded by CIHR. To date, we have recruited 69 patients from 4 sites and confirmed the feasibility of the study protocol. There was good compliance with study procedures and >94% of prescribed doses of study medication were delivered. Currently 3 patients have been lost to follow-up at 6 months. We have made several small protocol changes to clarify and improve the efficiency of the protocol and further reduce loss to follow-up, all of which are incorporated into this version of the protocol. We intend to roll the data from the pilot trial into this current proposal so we have not analyzed the trial by group.

2.20 Sub-Study Physical outcome assessment

In the context of this larger SUSTAIN trial, we propose an optional sub-study to enable the assessment of the functional recovery of included patients using the well-established and standardized 6-minute walk test (6MWT), a highly reproducible and easily assessable outcome.[81] Although this test is commonly used in cardiac patients, few data is available for performing the 6MWT in high-risk cardiac surgery patients. Fiorina et. al has already shown feasibility after low risk cardiac surgery in the rehabilitation program.[82] With the population enrolling in this study, we want to show the feasibility of the walking test in high-risk cardiac surgery patients and evaluate the differences between the 2 treatment groups. The assessment will be done once at the day of hospital discharge and if possible during the next routine follow-up visit at the hospital or clinic.

We are planning to enroll 224 patients (112 patients per group) in this sub-study. The inclusion criteria of the definitive SustainCSX study remain unchanged, focusing on high-risk cardiac surgery patients with prolonged ICU stay.

Outcomes for the SustainCSX sub-study:

1. Short-term performance-based physical function outcomes. To determine if a high-dose sodium-selenite administration, compared placebo, improves in-hospital muscle strength and performance-based physical functioning outcomes in critically ill patients, using a primary endpoint of six-minute walk distance (6MWD) at hospital discharge. If possible, and patients are seen at the hospitals or clinic within 3 months, we will do a reassessment of the 6MWT (optional).

Sample Size and Duration:

The sample size was estimated for the Wilcoxon Mann Whitney test with equal group size and alpha=0.05 for the two-sided test. Under the assumption of SD=130 and power of 0.8 the elongation of the 6MWT by 50m [28] would require a total of 224 evaluable patients with equal group size (112 per Group). Calculations were done using proc power in SAS 9.3.

Table 1: Results SAS proc power

Extension (m)	SD	Power	N Total (1:1)
50	120	0.8	192

50	120	0.9	256
50	130	0.8	224
50	130	0.9	300

(corrected N Total for 50 m MID)

3. TRIAL MANAGEMENT

3.1.1 What are the arrangements for the day-to-day management of the trial?

Dr. Daren Heyland is the Nominated Principal Investigator of this study. He is a Professor of Medicine and Epidemiology at Queen's University, Kingston, Ontario Canada. He is trained in Internal Medicine, Critical Care Medicine, and Clinical Epidemiology. He has a variety of research interests which include 3 Canadian Institutes of Health Research (CIHR) funded programs of research and has conducted more than 20 randomized trials in the areas of nutrition, infection, and end of life care.

The coordinating center for this study is located at the Clinical Evaluation Research Unit (CERU) at the Kingston General Hospital, Ontario, Canada (see www.ceru.ca). Dr. Heyland is the Director of CERU. The mission of CERU is to improve the care of acutely ill patients through knowledge generation, synthesis, and translation in a manner that will translate into improved clinical outcomes for sick patients and improved efficiencies to our health care systems. As such, CERU consists of a staff with experience and resources to support the successful completion of all phases of the design, conduct, monitoring, and interpretation of multicenter clinical studies. Dr Heyland's Senior Project Leader at CERU, will take overall responsibility for the day-to-day conduct of the trial, including supervision of all trial staff, training and liaising with the sites, conducting site visits, arranging all trial meetings, and reporting the progress of the trial to the steering committee. The data manager and statistician at CERU will be responsible for all aspects of data collection and processing, including processing of trial entry data, data entry, questionnaire monitoring, following up on missing information and non-responses. The applications developer will implement the web-based data entry/query/monitoring/reporting system for efficient conduct of the trial including the randomization, timely dispatch of questionnaires, automatic form monitoring, data validation and cleaning, and will work with the statistician to undertake the formal analysis and reporting of the data. For the European component of this trial, the Clinical Trial Center Aachen (CTC-A) in Germany will collaborate with CERU and will be responsible for the European regulatory (including BFARM) and ethics applications.

3.1.2 Reporting of Serious Adverse Events

Patients will be monitored daily for unexpected serious adverse events until death or discharge and will be reported by the participating site to CERU (the coordinating centre) within the established timelines i.e. an initial report within 24 hours and a follow up report within 10 days of becoming aware of the event. Serious adverse events thought to be related to the study drug (selenium) will be reported by CERU to Health Canada and other regulatory authorities and the manufacturer of the investigational product in an expedited manner, with reporting of these events if they are not related within the necessary timelines.

3.2 What will be the role of each principal applicant and co-applicant proposed?

Dr. Heyland will be responsible for all methodological and operational details of the trial. Dr. Bernard McDonald, co-principal investigator, is a cardiac anaesthesiologist and critical care medicine specialist at the University of Ottawa Heart Institute. He is the Medical

Director of the Cardiac Surgical Unit which provides care for approximately 1400 cardiac surgical patients per annum ranging from routine to the most advanced, complex life support methods available. He has a PhD in Pharmacology and has been a site investigator for several multi-centred clinical trials in cardiac surgical patients. Given his clinical and research expertise, he will be responsible for clinical and Canadian networking issues that emerge during the trial. Other members of the Steering Committee, including Drs. Lamarche, Fremes, and Fowler, will also function as site investigators at their local sites. Andrew Day is the Senior Biostatistician at the Clinical Evaluation Research Unit and will be responsible for aspects of data analysis and reporting. Dr. Thavorn is a health economist and a scientist at the Ottawa Methods Centre, the Ottawa Hospital Research Institute. She will be responsible for a cost-utility analysis and health system aspects of the trial. Finally, international members of our team include: Dr. Christian Stoppe (co-principal investigator) and Professor Patrick Meybohm (coordinating investigator of the German sites), both cardiac anesthesiologists and intensive care specialists, will manage the German part of the study and take over the responsibility for the operational details of the study in Germany and use the already established RIPHeart study group network, which exists of about 20 cardiac surgery centers in Germany (recently published a large-scale trial in cardiac surgery in the *New England Journal of Medicine*).[69] Dr. William Manzanares, an Associate Professor at the Department of Critical Care Medicine, Universidad de la República, Montevideo-Uruguay is an established selenium expert in the field as well as a member of the team.

3.3 Describe the trial steering committee and if relevant the data safety and monitoring committee.

Together, Drs. Heyland, Stoppe and McDonald, the 3 principal investigators, along with senior CERU staff will form the Executive Committee. The Executive Committee will be responsible for the day-to-day management of the trial. They will be supported by the Steering Committee consisting of all co-investigators that will provide specific scientific and operational input. The terms of references and details of these committees is contained in Appendix 5. Finally, we have constituted a Data Monitoring Committee that will periodically monitor the safety reports and other aspects of quality management related to this trial.

Appendix 1: Figures and Tables

Figure 1

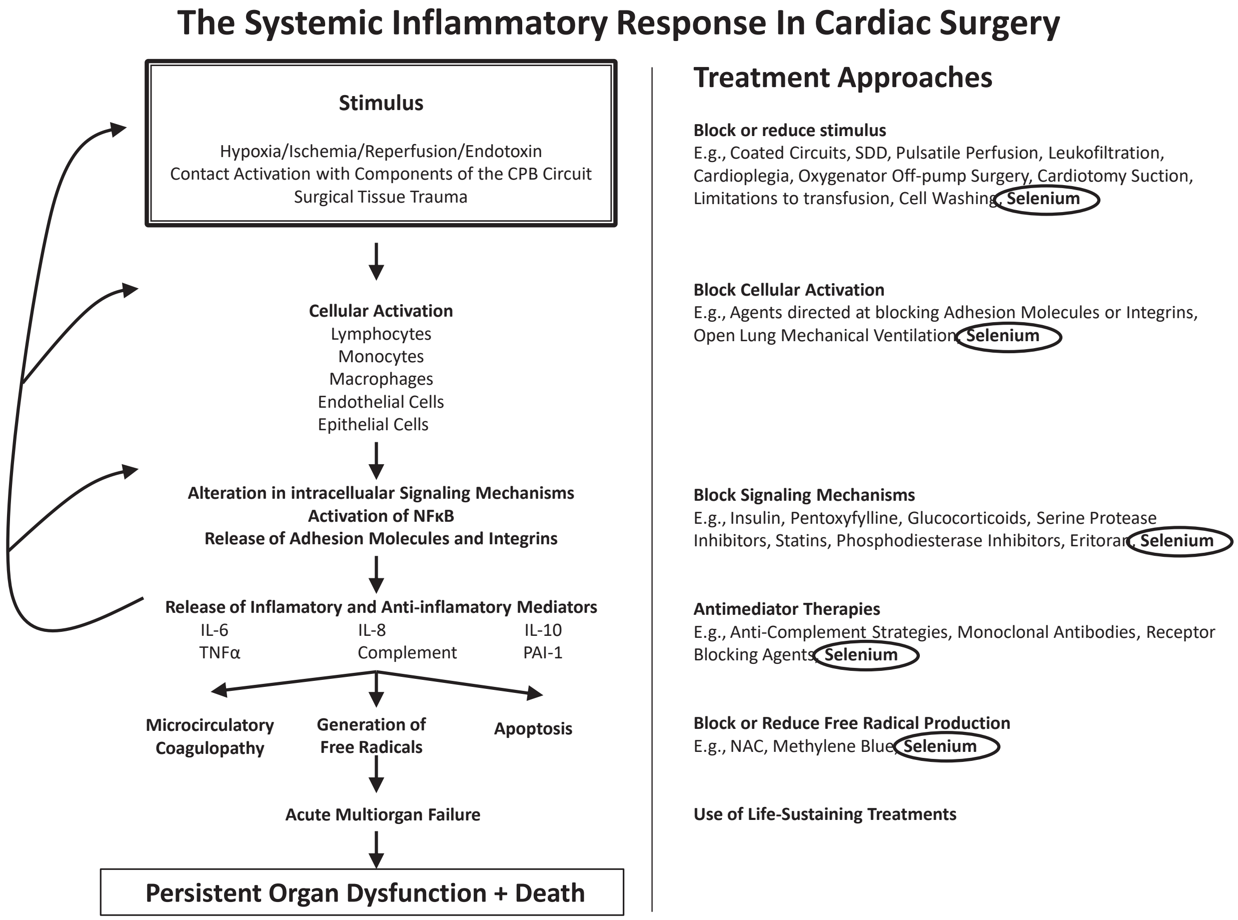

Adapted with permission from Rick Hall7

Figure 2. Pharmacodynamic profile of two high doses of selenite assessed by GPx-3 activity

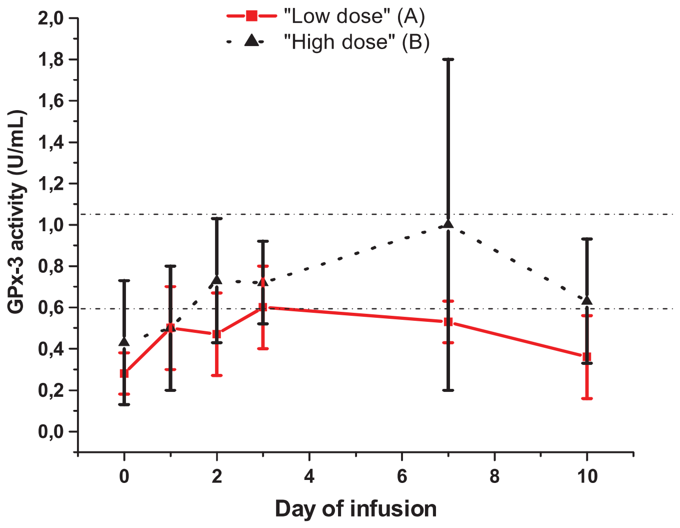

GPx-3 increases with both doses. However, only the very high dose reaches physiological levels (0.72±0.16 U/mL) in patients with SIRS. After day 7, GPx-3 decreases in both groups, but only the very high dose group maintained physiologic levels until day 10 (0.63±0.30 versus 0.36±0.20 U/mL, P= .136). GPx-3: extracellular glutathione peroxidase.

Table 1. Overview of clinical trials investigating the role of selenium in patients undergoing cardiac surgery

Reference	Study design	Population	Selenium salt from and dosing regime	Outcomes
Leong 2010 [35]	Randomized controlled trial (double-blind)	Patients undergoing elective CABG and/or valve surgery<br>n = 117	Coenzyme Q_{10}, magnesium orotate, lipoic acid, omega-3 fatty acids and selenium versus placebo (approx. 2 months before and 1 month after surgery)	Metabolic therapy reduced plasma troponin I, 24 hours postoperatively from 1.5 (1.2–1.8) (geometric mean 95% CI) µg/L, to 2.1 (1.8–2.6) µg/L (P = 0.003) and shortened the mean length of postoperative hospital stay by 1.2 days from 8.1 (7.5–8.7) to 6.9 (6.4–7.4) days (P = 0.004) and reduced hospital costs. Metabolic therapy was inexpensive and had no clinically significant side effects.
Stoppe 2011 [32]	Prospective observational study	Patients scheduled for cardiac surgery with CPB<br>n = 60	-	Fifty patients exhibited a significant selenium deficiency already before surgery. In all patients, blood levels of selenium, copper, and zinc were significantly reduced after end of surgery when compared to preoperative values (selenium: 89.05 ± 12.65 to 70.84 ± 10.46 µg; zinc: 5.15 ± 0.68 to 4.19 ± 0.73 mg/L; copper: 0.86 ± 0.15 to 0.65 ± 0.14 mg/L; p < .001). Selenium concentrations at end of surgery were independently associated with the postoperative occurrence of multiorgan failure (p =0.0026, odds ratio 0.8479, 95% confidence interval 0.7617 to 0.9440).
Koszta 2012 [53]	Prospective observational study	Patients scheduled for cardiac surgery with CPB<br>n = 197	-	Selenium levels were significantly lower in non-survivors 102.2 ± 19.5 µg/L compared with survivors 111.1 ± 16.9 µg/L (*p* = 0.047), and the mean age, EuroSCORE values, and troponin concentrations were significantly higher in the non-survivors. Lower selenium levels identified as a risk factor for postoperative mortality.
Stoppe 2013 [34]	Prospective observational study	Patients scheduled for cardiac surgery with CPB<br>n = 104	Intravenous bolus of 2.000 µg selenium after induction of anesthesia and 1.000 µg selenium every day further during ICU stay	Preoperative sodium-selenite administration increased selenium blood concentrations to normal values on ICU admission, but failed to prevent a significant decrease of circulating selenium on the first postoperative day.
Stevanovic 2014 [33]	Randomized controlled trial (comparison: off- versus on-pump CABG)	Patients undergoing elective CABG<br>n = 40	-	Both groups showed a comparable decrease of circulating selenium concentrations. Likewise, levels of oxidative stress and IL-6 were comparable in both groups. Selenium levels correlated with antioxidant capacity (GPx: r = 0.720; p<0.001) and showed a negative correlation to myocardial damage (CK-MB: r = −0.571, p<0.001). Low postoperative

				selenium levels had a high predictive value for the occurrence of any postoperative complication.
Sustain CSX Trial 2014	Randomized controlled trial (double-blind)	Patients undergoing CABG plus valve surgery, multiple valve replacement surgery, patients with a high perioperative risk profile (≥ 5% EuroSCORE II).<br>n = 1.400	Intravenous bolus of 2.000 μg selenium after induction of anesthesia and 1.000 μg selenium every day further during ICU stay	On-going, recruiting
Haberthuer<br>ClinicalTrials.gov Identifier: NCT01141556	Randomized controlled trial (double-blind)	Elective all-cause cardiac surgery<br>n = 410	Loading dose of 4.000 μg, daily dosage of 1.000 μg of selenium versus placebo	On-going, on analysis

Figure 3. Plasma Levels Selenium in REDOXS Trial[30]

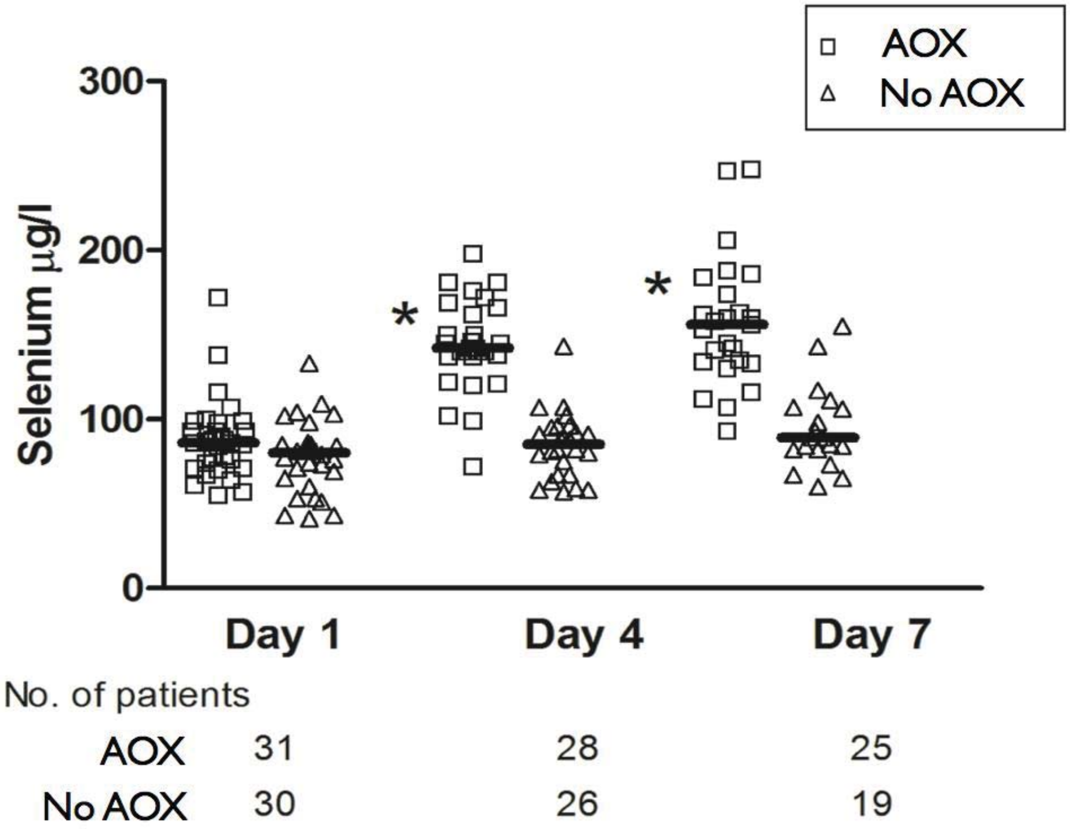

Figure shows plasma levels of selenium in the sub-study patients. Normal range of selenium 58-234 ug/l. The shaded gray areas on graphs represent the normal plasma levels of glutamine and selenium. Horizontal bars represent medians and asterisks indicate a statistically significant difference between arms at P<0.05 according to the Wilcoxon rank-sum test. A total of 66 patients participated in the laboratory sub-study but not all patients had a measurement every day. AOX denotes antioxidants.

Figure 4A. Evaluation of intraoperative decreases of antioxidant trace elements (in % from baseline) in the different subgroups of patients that underwent elective cardiac surgery.

The biggest selenium depletion was observed in patients developing multiorgan dysfunction (MOD) in the postoperative period.
The lower boundary of the box indicates the 25th percentile, the line within the box marks the median, and the upper boundary of the box indicates the 75th percentile. Whiskers indicate the 90th and 10th percentiles, whereas close circles symbolize the 95th and 5th percentile.
p-values for the Kruskal-Wallis-ANOVA are depicted in the legend.
* = p< 0.05 vs. no organ dysfunction (NOD); † = p < 0.05 vs. single organ dysfunction (SOD)

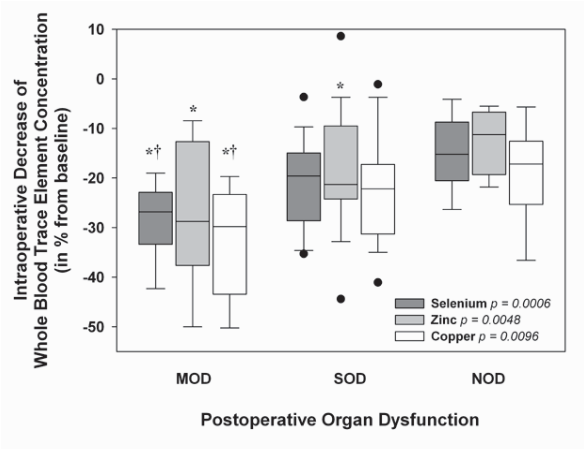

Figure 4B. Measurement of perioperative time course of whole blood concentrations of selenium in cardiac surgical patients who received a perioperative selenium supplementation.

The shaded area indicates the reference range for whole blood selenium concentration in Germany. BL: Baseline before induction of anesthesia; AD: admission to the ICU; 4hrs: 4 hours after admission to ICU.

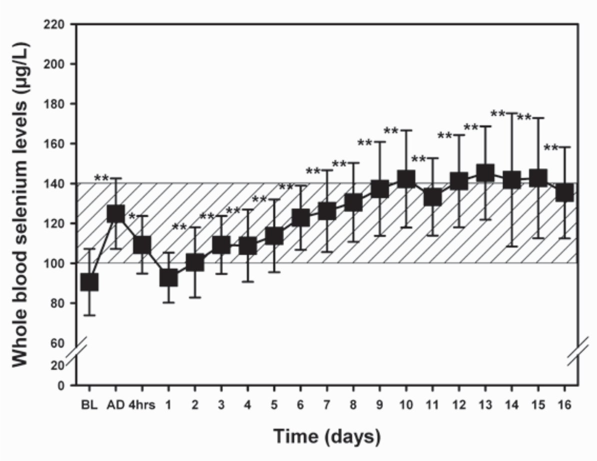

Appendix 2: Description of Study Measures and Outcomes

Baseline Demographics and Laboratory

Age, sex, height, weight, primary diagnosis, CCS angina classification, NYHA heart failure classification, left ventricular ejection fraction (LVEF) major co-morbid illnesses including those that comprise the Euroscore II, calculated Euroscore II, cardiovascular medications, insulin, and use of preoperative systemic corticosteroids will be recorded. To better understand the effect of critical illness and persistent organ dysfunction following cardiac surgery, baseline assessments of functional status with Barthel ADL, Clinical Frailty Scale, and health related quality of life (HRQOL) with SF-36 (see below: *Long Term Outcomes*) will be recorded. Baseline hemoglobin and platelet count, HgBA1C, INR, creatinine and estimated GFR will be drawn within 4 weeks of surgery and the values most immediate to the operation will be recorded.

The Surgical Procedure

The surgical procedure and duration will be recorded. The duration of the surgical procedure is defined as as the time interval between start of the surgery procedure (skin incision) and end time of surgery (last dressing). The duration of cardiopulmonary bypass will be measured as the time interval between the start time and the end time of bypass. If bypass is restarted, duration(s) will be added.The duration of aortic clamping will be measured as the time interval between the start time and the end time of aortic clamping. Use of intraopearative cell salvage, and ultrafiltration will be recorded. Lowest hematocrit on CPB and intraoperative transfusion of blood products will be recorded.

Perioperative hemodynamic profile and laboratory measures

The following hemodynamic parameters will be recorded post anesthetic induction, upon ICU admission and at change of nursing shift on the morning of the first postoperative day (if available): HR, systemic and pulmonary blood pressures, central venous pressure, cardiac output, and mixed venous blood oxygen saturation level (SvO2). Complete blood count, INR, blood lactate, and creatinine measures will be drawn at these same time points; in most institutions this will approximate standard care.

Laboratory

If available, complete blood count, INR, blood lactate, and creatinine measures will be will be recorded post anesthetic induction, upon ICU admission and at change of nursing shift on the morning of the first postoperative day. In most institutions this will approximate standard care. After the first postoperative day, standard lab tests to detect myocardial ischemia (e.g Troponin), inflammation, and organ function (creatinine, urea, bilirubin, and hemoglobin) will be done according to clinical routine and as clinically indicated.

Surgical and Cardiovascular complications

Time and date of surgical re-opening (return to the operating room for mediastinal bleeding with or without tamponade, graft occlusion, valve dysfunction, or other cardiac reason), re-operation, delayed sternal closure, and postoperative cardiac arrest will be recorded where applicable. Myocardial infarction is defined within 72 hours of surgery as CK-MB or troponin $\geq$ 14 times the upper limit of normal (xULN) with electrocardiographic changes consistent with myocardial injury or CK-MB or troponin $\geq$ 20 xULN in all patients; or angiographic or autopsy evidence of graft occlusion or new native coronary artery occlusion. Late perioperative myocardial injury (later than 72 hours after surgery) is defined as ECG changes

consistent with myocardial infarction (new significant Q waves in two contiguous leads) or evolving ST-segment or T-wave changes in two contiguous leads signifying ischemia or new left bundle branch block or ST segment elevation and elevated cardiac markers (troponin or CK-MB) in the necrosis range. ECGs will be done preoperatively, at 24 hours postoperatively (day 1 in the intensive care unit), and just prior to hospital discharge or on postoperative day 4 to 6 whichever comes first. CK-MB (whichever test is available at the hospital) will be measured preoperatively, at 8 hours, and at 16-24 hours postoperatively. The incidence of arrhythmias including atrial fibrillation, cardiac arrest, or myocardial infarction will be recorded by group. We will record the incidence of stroke/cerebral vascular accident defined in accordance with as, any confirmed neurological deficit of abrupt onset caused by a disturbance in blood supply to the brain that does not resolve within 24 hour. Finally, we will record the number of patients who, within 30 days postoperatively, develop deep sternal wound infection involving muscle, bone, and/or mediastinum requiring operative intervention. Patients must have all of the following conditions to receive this diagnosis:
-Wound opened with excision of tissue (I&D) or re-exploration of mediastinum
-Positive culture unless patient on antibiotics at time of culture or no culture obtained
-Treatment with antibiotics beyond perioperative prophylaxis

Calculation of Persistent Organ Dysfunction and Death (POD + Death)
We have proposed POD + death as a novel composite outcome measure for this cardiac surgical study population. We define POD as the need for one or more life-sustaining therapies (mechanical ventilation, vasopressor and inotrope therapy, mechanical circulatory support, continuous renal replacement therapy, or intermittent hemodialysis).

a. *Mechanical ventilation:* Duration of mechanical ventilation will calculated from time of admission to ICU until time of discontinuation. All times of endotracheal extubation and any subsequent re-intubation/re-extubations or tracheostomy will be recorded. A patient will be considered liberated from mechanical ventilation if they remain off mechanical ventilation for more than 48 hours i.e. for a patient who is removed from mechanical ventilation and reintubated 30 hours later, the intervening days from extubation to re-intubation will be considered to represent ongoing need for life support. Use of non-invasive mechanical ventilation (CPAP or BiPAP) will count as mechanical ventilation unless the patient routinely uses these modalities at home.
b. *Vasopressor therapy:* We will document an ongoing need for vasopressor agents such as norepinephrine, epinephrine, vasopressin, ≥5 μg/ /minute of dopamine, or ≥50 μg/minute phenylephrine when administered continuously for more than one hour on any day
c. *Mechanical Circulatory Support (MCS):* We will record times of initiation, discontinuation and duration of mechanical circulatory support (Intra-aortic balloon pump (IABP) or extra corporeal membrane oxygenation (ECMO). A patient will be considered liberated from MCS if they remain off MCS for more than 48 hours
d. *Renal Replacement therapy:* We will record mode of renal replacement therapy (Continuous renal replacement and/or intermittent hemodialysis), and times/dates of initiation, discontinuation and duration. A patient will be considered liberated from renal replacement therapy if they remain off therapy for more than 48 hours for continuous renal replacement or 72 hours in the case of intermittent hemodialysis.

Evaluation of postoperative infectious complications
History, clinical symptoms, physical exam, and laboratory findings suggesting the presence of infection that justified the initiation of anti-infective therapy (prophylactic therapy not included) will be used to adjudicate the presence or absence of infection, as per our previously published ICU studies.[30] We have created standard definitions for all possible ICU-acquired infections and they are expressed in degrees of certainty (definite, probable, and possible). Furthermore, we will classify all infections as microbiologically documented (infection confirmed by positive cultures of blood or body fluid from a suspected site) or just clinically suspected (an infection can be clinically documented if there is gross purulence or abscess but not microbiologically confirmed (cultures remain sterile due to antibiotic therapy). We will record the incidence of deep sternal wound infection (as defined above).

ICU and Hospital length of stays
ICU length of stay is calculated from time of ICU admission to time and date of actual discharge. Hospital length of stay will be calculated from the date of index surgical procedure to time and date of discharge from hospital

Long-term outcomes
To better understand the impact of study treatments on longer-term survival and health-related quality of life (HRQOL) and to study the effect of critical illness and persistent organ dysfunction on outcome following cardiac surgery, we will follow surviving study patients for 6 months. To date, there is only limited data on the long term HRQOL and functional status of patients who have experienced persistent organ dysfunction following cardiac surgery. There exists no published Canadian data and the few publications from single European [83,84,85,86,87, 88] and American centers[61] are retrospective and with no baseline information, have varying inclusion criteria and employ a cross sectional design with varying follow up timesAs a result, the literature is contradictory as to "whether the effort is worthwhile" for patients who experience major morbidity post cardiac surgery. With this trial, we have a unique opportunity to obtain baseline premorbid information on functional status and HRQOL and to prospectively follow and compare patients with major morbidity and organ dysfunction to those with uncomplicated recovery at fixed intervals and across centers.

At POD 30, 3 and 6 months post randomization, a trained research coordinator at each site will contact patients discharged from hospital to assess survival status, whether they have resumed normal activities, and administer the SF-36 and assess Barthel ADL status over the phone. The SF-36 is a multipurpose survey of general health status consisting of eight domains and 36 items and has been used in a variety of patient populations.[89] Compared to other generic health status instruments, the SF-36 has been shown to have better feasibility, internal consistency, content validity, discriminative ability and is more responsive to clinical improvement.[90] To measure change in HRQOL with cardiac surgery, SF-36 has been the instrument most commonly chosen by investigators.[91] Recently, we have demonstrated that the SF-36 has good reliability and validity when used to measure HRQL in survivors of critical illness.[92] The Barthel ADL scale was originally designed to assess overall performance in cancer patients but has become the most commonly chosen tool to assess long term functional status post cardiac surgery.[93] We justify only following patients for 6 months as we have previously demonstrated that most of the improvements in QOL will have occurred by then.[94] We have used this approach in ongoing studies at CERU examining the long-term outcomes of critically ill patients.[30,92,94]

Appendix 3: Sample Size Consideration

Our primary outcome for this trial is days alive and free of life sustaining therapy (PODS) within the first 30 days after surgery (hereafter referred to as "*free days*"). We truncate the primary outcome at 30 days because there is virtually no loss to follow-up during this period and there are only about 5% of outlying patients who remain alive and dependent on life sustaining therapy beyond (sometimes months beyond) 30 days. Patients who die during the first 30 days or are only liberated from life sustaining therapy after 30 days are assigned 0 *free days*. This outcome is similar to "ventilator free days" in the first 30 days which is widely accepted and commonly used in intensive care research.[70, 71, 72]

We estimated the control arm distribution of *free days* from our database of all patients that underwent cardiac surgery between 01/01/2011 and 31/12/2011 at the university hospital of Aachen, Germany (n=1127) and met present inclusion criteria (n=170). In this dataset the mean (STD) free days was 23.2 (9.2) factoring in the 4% mortality rate. The distribution and cumulative distribution of free days are provided in figures 1 and 2 respectively. It may be seen that 4% of the patients died, 6% survived but had 0 free days and 58% had 26 to 29 free days. Only 15% of patients had between 1 and 20 free days. These numbers are consistent with the 4% 30-day mortality rate and the 6% not yet free from life sustaining therapy by 30 days that we observed from the 83 patients seen at the University of Ottawa Heart Institute between July 1, 2012 and May 31 2013 who met the eligibility criteria of the current proposal. Similar to "ventilator free days", the distribution of *free days* is clearly not Gaussian with most of its distribution near the minimum and maximum possible values of 0 and 30 respectively.

We performed simulation in order to accurately estimate the actual power of applying the Wilcoxon Rank-Sum test to scenarios with various effect sizes where the control arm had the same distribution as observed from our German data. The simulation generated data in the control arm where the mortality rate was 4% and the daily rate (i.e. hazard) of being liberated from life sustaining therapy was the same as observed with our German data. The intervention arm was then generated by multiplying the control arm daily rate of liberation by some fixed factor (i.e. hazard ratio). The mean days on life sustaining therapy was then subtracted from 30 to obtain the *free days*. All estimates are based on simulating 10, 000 samples of the required sample size so power estimates will have more than a 95% chance of being accurate to within 1%. It may be seen from figures 1 and 2 that the distribution of the simulated control arm was nearly identical to the distribution of the actual observed German data. Figures 1 and 2 also provide the distribution of the intervention arm when the intervention causes a 20% relative increase in the daily rate of liberation from life sustaining therapy compared to the control arm.

Table 1 provides the simulation results. We expect that the sample size of the definitive trial will be around 700 per arm. This would provide about 90% power to detect a 20% relative increase in the daily rate of liberation from life sustaining therapy. Such an effect size would result in a mean decrease of 1.5 days in the days of life sustaining therapy from 6.8 days to 5.3 days, or equivalently, a 1.5 day increase from 23.2 to 24.7 *free days*. We believe such an effect size is plausible and is in line with minimally clinically important differences accepted in other recent major trials in the ICU setting. [64,] **Error! Bookmark not defined.**

Although the simulation results are reassuring, it may be of interest to note that with sample sizes as large as we are proposing, the standard sample size formula for comparing the mean of a Gaussian variable between two independent groups with unequal variance agreed almost exactly with our simulations (table 1).

Figure 1: Distribution of Free Days

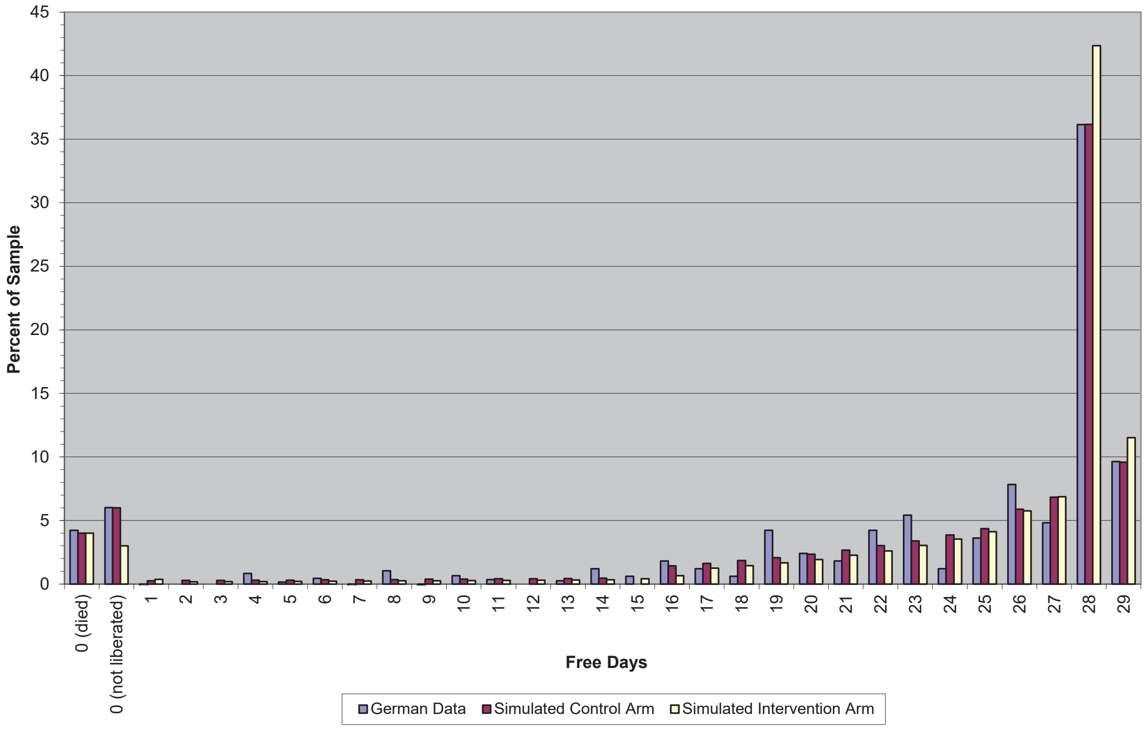

Figure 2: Cumulative Distribution of Free Days

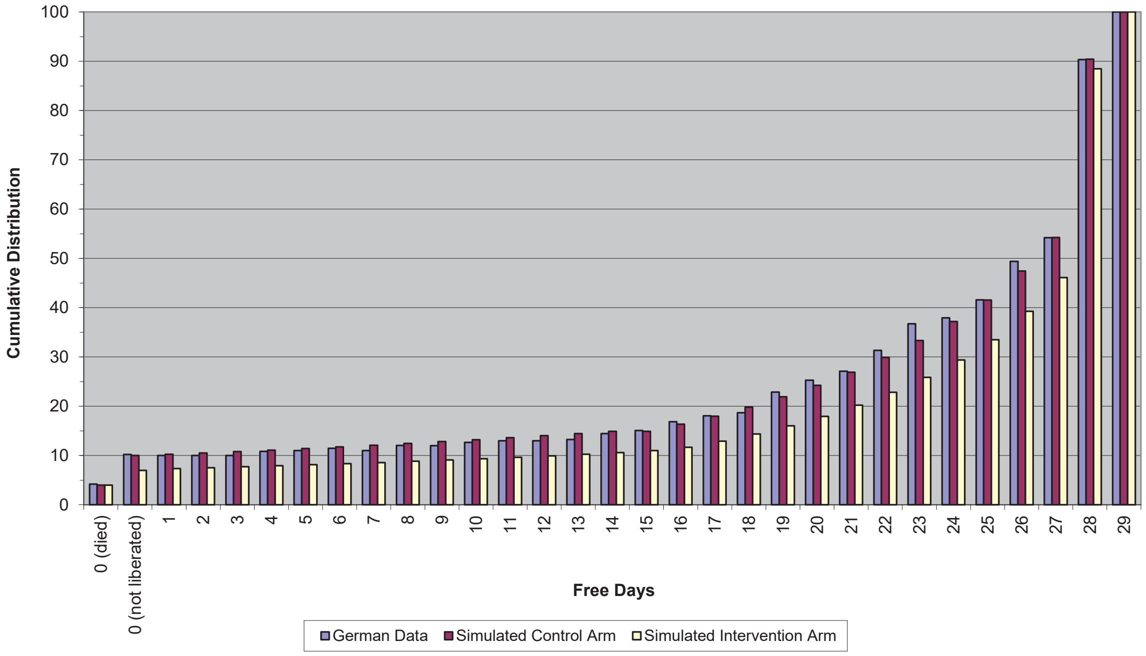

Table 1: Total Sample Size Required to Achieve 90% Power at Two-sided alpha=0.05

Amount treatment multiplies daily chance of liberation from life sustaining therapy among survivors	Mean days on life sustaining therapy*	Mean (std) days alive and free of life sustaining therapy	Mean increase in Free Days	Total sample size required according to simulation	Exact power for t-test if data was Gaussian
1	6.8 (9.2)	23.2 (9.2)	0.0	**	5%
1.1	6 (8.7)	24.0 (8.7)	0.8	5300	90%
1.15	5.6 (8.4)	24.4 (8.4)	1.2	2400	92%
1.2	5.3 (8.2)	24.7 (8.2)	1.5	1400	90%
1.25	5.2 (7.9)	25.0 (7.9)	1.8	940	89%
1.5	4 (7.0)	26.1 (7.0)	2.9	280	84%
2	2.6 (6.0)	27.4 (6.0)	4.2	92	73%

The control arm follows the distribution from our German data (n=170) which had a mean (std) of 23.2 (9.2) free days. The intervention arms have had the daily rate of liberation from life sustaining therapies among survivors multiplied by the factor in the first column.

* a value of 30 is assigned if the patient died within 30 days or was not liberated from life sustaining therapies within 30 days. All simulations assume a 4% mortality rate in both arms.

** with no effect of treatment, the type I error rate is maintained at 5% regardless of sample size.

Appendix 4: Proposed Sites

Country	Site	Site Investigator
Germany	1) RWTH Aachen University	Goetzenich/Stoppe
	2) Universitätsklinikum Frankfurt	Patrick Meybohm
	3) Universitätsklinikum Schleswig-Holstein, Kiel	Gunner Elke
	4) Uniklinikum Giessen und Marburg, Giessen	Böning/ Sander
	5) Universitätsklinikum Mainz	Laufenberg-Feldmann
	6) Universitätsklinikum Schleswig-Holstein, Lübeck	Heringlake/ Stehr
	7) Universitätsklinikum Bonn	Wittmann/Böhm
	8) Universitätsklinikum Düsseldorf	Meyer-Treschan
	9) Universität Oldenburg	Weyland
	10) Ludwig-Maximilians-Universität (LMU) München	Zwissler/Kilger/Briegel
	11) Universitätsklinikum Freiburg	Beyersdorf/Lechner
Canada	1) University of Ottawa Heart Institute	Bernard McDonald
	2) Sunnybrook Health Sciences Centre	Stephen Fremes
	3) Institut de cardiologie de Montreal	Yoan Lamarche
	4) Vancouver General Hospital	Rael Klein
	5) St. Paul's Hospital, Vancouver	Bobby Lee
	6) University of Alberta, Edmonton	Sean Bagshaw
	7) Calgary	Andre Ferland
	8) McMaster University, Hamilton	Richard Whitlock
	9) Toronto General Hospital	Angela Jareth
	10) St. Michael's, Toronto	David Mazer
	11) UWO, London	Phil Jones
	12) Dalhousie	Blaine Kent

Appendix 5: Committee Terms of Reference

Executive Committee Terms of Reference

1. Purpose

The purpose of the Executive Committee Terms of Reference is to:
a. Describe the principal responsibilities of the Executive Committee.
b. Specify the purpose and frequency of the meetings.
c. Define authorship privileges and limitations.

2. Membership

The core research team members intimately involved in the scientific leadership, operations (implementing), successful completion of study, analysis of data and manuscript writing. Membership will be comprised of Dr. Heyland, Dr. Stoppe and MacDonald and project leaders at CERU. Dr. Meybohm will take over the coordination of European/German participating centers.

3. Terms of Reference

The Executive Committee will:

- Be responsible for the good conduct of the clinical trial, including implementation and execution. Aid the Sponsor in handling his responsibilities according to Good Clinical Practice.
- Review/and modify the Standard Operating Procedures regarding the daily operations of the clinical trial
- Review the data analysis plan for the trial
- Modify/review the design, execution and analysis of the clinical trial, if needed
- Review all recommendations and data from the Coordinating Center.
- Report all safety concerns encountered to the Data Monitoring Committee
- Review all recommendations pertaining to the conduct of the trial from the Data Monitoring Committee
- Approve all secondary research involving participating patients proposed by investigators
- Assume ultimate responsibility for the final results of the study.

4. Meetings

The Executive Committee will:

- Have regular conference calls and occasional face to face meetings as deemed necessary by the Principal Investigators.
- Meet bi-weekly or monthly prior to study initiation; bi-monthly or as needed thereafter.
- Review meeting agendas and pre-circulated documents prior to the meetings.
- Provide input via email if unable to attend scheduled meetings.

A quorum will consist of 3 members.

Steering Committee Terms of Reference

1. Purpose

The purpose of the Sustain CSx Steering Committee Terms of Reference is to:

a. Describe the principal responsibilities of the Steering Committee

b. Specify the purpose and frequency of the meetings.

c. Define authorship privileges and limitations.

2. Membership

A broader committee made up of the Executive Committee members and co-investigators and/or experts who provide guidance on key issues.

3. Terms of Reference

The Steering Committee will:

- Be responsible for the good conduct of the clinical trial, including implementation and execution. Aid the Sponsor in handling his responsibilities according to Good Clinical Practice.
- Review all recommendations and data from the Coordinating Center.
- Review all recommendations pertaining to the conduct of the trial from the Data Monitoring Committee
- Communicate preliminary results or changes in protocol to Research Coordinators.
- Be acknowledged as the Sustain CSx Trial Steering Committee in primary publications arising from the study.

4. Meetings

The Steering Committee will:

- Meet every 2-4 months via conference calls or face to face meetings.
- May be consulted on an ad hoc basis on specific strategic issues.
- Review meeting agendas and pre-circulated documents prior to the meetings.
- Provide input via email if unable to attend scheduled meetings.

A quorum will consist of 6 members.

References

[1] Grover A, Gorman K, Dall TM, Jonas R, Lytle B, Shemin R, Wood D, Kron I. Shortage of cardiothoracic surgeons is likely by 2020. Circulation. 2009 Aug 11;120(6):488-94.

[2] Brown PP, Kugelmass AD, Cohen DJ, Reynolds MR, Culler SD, Dee AD, Simon AW. The frequency and cost of complications associated with coronary artery bypass grafting surgery: results from the United States Medicare program. Ann Thorac Surg. 2008 Jun;85(6):1980-6.

[3] Herman CR, Buth KJ, Légaré JF, Levy AR, Baskett R. Development of a predictive model for major adverse cardiac events in a coronary artery bypass and valve population. J Cardiothorac Surg. 2013 Jul 30;8:177.

[4] Suleiman MS, Zacharowski K, Angelini GD. Inflammatory response and cardioprotection during open-heart surgery: the importance of anaesthetics. Br J Pharmacol. 2008 Jan;153(1):21-33.

[5] Kloner RA, Jennings RB. Consequences of brief ischemia: stunning, preconditioning, and their clinical implications: part 1. Circulation. 2001 Dec 11;104(24):2981-9.

[6] Comunale ME, Body SC, Ley C, Koch C, Roach G, Mathew JP, Herskowitz A, Mangano DT. The concordance of intraoperative left ventricular wall-motion abnormalities and electrocardiographic S-T segment changes: association with outcome after coronary revascularization. Multicenter Study of Perioperative Ischemia (McSPI) Research Group. Anesthesiology. 1998 Apr;88(4):945-54.

[7] Hall R. Identification of inflammatory mediators and their modulation by strategies for the management of the systemic inflammatory response during cardiac surgery. J Cardiothorac Vasc Anesth. 2013 Oct;27(5):983-1033.

[8] Dieleman JM, Nierich AP, Rosseel PM, van der Maaten JM, Hofland J, Diephuis JC, Schepp RM, Boer C, Moons KG, van Herwerden LA, Tijssen JG, Numan SC, Kalkman CJ, van Dijk D; Dexamethasone for Cardiac Surgery (DECS) Study Group. Intraoperative high-dose dexamethasone for cardiac surgery: a randomized controlled trial. JAMA. 2012 Nov 7;308(17):1761-7.

[9] Whitlock R, Teoh K, Vincent J, Devereaux PJ, Lamy A, Paparella D, Zuo Y, Sessler DI, Shah P, Villar JC, Karthikeyan G, Urrútia G, Alvezum A, Zhang X, Abbasi SH, Zheng H, Quantz M, Yared JP, Yu H, Noiseux N, Yusuf S. Rationale and design of the steroids in cardiac surgery trial. Am Heart J. 2014 May;167(5):660-5.

[10] Whitlock RP, Devereaux PJ, Teoh KH, Lamy A, Vincent J, Pogue J, et. al. Methylprednisolone in patients undergoing cardiopulmonary bypass (SIRS): a randomised, double-blind, placebo-controlled trial. Lancet 2015; 386: 1243–53.

[11] Navarro-Alarcon, M.; Cabrera-Vique, C. Selenium in food and the human body: A review. Science of the total environment 2008, 400, 115–141.

[12] Fairweather-Tait, S. J.; Bao, Y.; Broadley, M. R.; Collings, R.; Ford, D.; Hesketh, J. E.; Hurst, R. Selenium in human health and disease. Antioxidants & Redox Signaling 2011, 14, 1337–1383.

[13] Heyland DK, Dhaliwal R, Suchner U, Berger MM. Antioxidant nutrients: a systematic review of trace elements and vitamins in the critically ill patient. Intensive Care Med. 2005 Mar;31(3):327-37.

[14] Lovat R, Preiser JC. Antioxidant therapy in intensive care. Curr Opin Crit Care. 2003 Aug;9(4):266-70.

[15] Steinbrenner H, Sies H. Protection against reactive oxygen species by selenoproteins. Biochim Biophys Acta. 2009 Nov;1790(11):1478-85.

[16] Moreno-Reyes R, Mathieu F, Boelaert M, Begaux F, Suetens C, Rivera MT, Nève J, Perlmutter N, Vanderpas J. Selenium and iodine supplementation of rural Tibetan children affected by Kashin-Beck osteoarthropathy. Am J Clin Nutr. 2003 Jul;78(1):137-44.

[17] Klotz LO, Kröncke KD, Buchczyk DP, Sies H. Role of copper, zinc, selenium and tellurium in the cellular defense against oxidative and nitrosative stress. J Nutr. 2003 May;133(5 Suppl 1):1448S-51S.

[18] Venardos KM, Perkins A, Headrick J, Kaye DM. Myocardial ischemia-reperfusion injury, antioxidant enzyme systems, and selenium: a review. Curr Med Chem. 2007;14(14):1539-49.

[19] Forman HJ, Torres M. Reactive oxygen species and cell signaling: respiratory burst in macrophage signaling. Am J Respir Crit Care Med. 2002 Dec 15;166(12 Pt 2):S4-8.

[20] Heyland DK, Dhaliwal R, Suchner U, Berger MM. Antioxidant nutrients: a systematic review of trace elements and vitamins in the critically ill patient. Intensive Care Med. 2005 Mar;31(3):327-37.

[21] Tolando R, Jovanovic A, Brigelius-Flohé R, Ursini F, Maiorino M. Reactive oxygen species and proinflammatory cytokine signaling in endothelial cells: effect of selenium supplementation. Free Radic Biol Med. 2000 Mar 15;28(6):979-86.

[22] Holzer R, Bockenkamp B, Booker P, Newland P, Ciotti G, Pozzi M. The impact of cardiopulmonary bypass on selenium status, thyroid function, and oxidative defense in children. Pediatr Cardiol. 2004 Sep-Oct;25(5):522-8.

[23] Manzanares W, Dhaliwal R, Jiang X, Murch L, Heyland DK. Antioxidant micronutrients in the critically ill: a systematic review and meta-analysis. Crit Care. 2012 Dec 12;16(2):R66.

[24] Benstoem C, Goetzenich A, Kraemer S, Borosch S, Manzanares W, Hardy G, Stoppe C. Selenium and its supplementation in cardiovascular disease--what do we know? Nutrients. 2015 Apr 27;7(5):3094-118.

[25] Motoyama T, Okamoto K, Kukita I, Hamaguchi M, Kinoshita Y, Ogawa H. Possible role of increased oxidant stress in multiple organ failure after systemic inflammatory response syndrome. Crit Care Med. 2003 Apr;31(4):1048-52.

[26] Forceville X, Vitoux D, Gauzit R, Combes A, Lahilaire P, Chappuis P. Selenium, systemic immune response syndrome, sepsis, and outcome in critically ill patients. Crit Care Med. 1998 Sep;26(9):1536-44.

[27] Sakr Y, Reinhart K, Bloos F, Marx G, Russwurm S, Bauer M, Brunkhorst F. Time course and relationship between plasma selenium concentrations, systemic inflammatory response, sepsis, and multiorgan failure. Br J Anaesth. 2007 Jun;98(6):775-84.

[28] Manzanares W, Biestro A, Torre MH, Galusso F, Facchin G, Hardy G. High-dose selenium reduces ventilator-associated pneumonia and illness severity in critically ill patients with systemic inflammation. Intensive Care Med. 2011 Jul;37(7):1120-7.

[29] Manzanares W, Biestro A, Galusso F, Torre MH, Mañáy N, Facchin G, Hardy G. High-dose selenium for critically ill patients with systemic inflammation: pharmacokinetics and pharmacodynamics of selenious acid: a pilot study. Nutrition. 2010 Jun;26(6):634-40.

[30] Heyland D, Muscedere J, Wischmeyer PE, Cook D, Jones G, Albert M, Elke G, Berger MM, Day AG; Canadian Critical Care Trials Group. A randomized trial of glutamine and antioxidants in critically ill patients. N Engl J Med. 2013 Apr 18;368(16):1489-97.

[31] Angstwurm MW, Engelmann L, Zimmermann T, Lehmann C, Spes CH, Abel P, Strauss R, Meier-Hellmann A, Insel R, Radke J, Schüttler J, Gärtner R. Selenium in Intensive Care (SIC): results of a prospective randomized, placebo-controlled, multiple-center study in patients with severe systemic inflammatory response syndrome, sepsis, and septic shock. Crit Care Med. 2007 Jan;35(1):118-26.

[32] Stoppe C, Schälte G, Rossaint R, Coburn M, Graf B, Spillner J, Marx G, Rex S. The intraoperative decrease of selenium is associated with the postoperative development of multiorgan dysfunction in cardiac surgical patients. Crit Care Med. 2011 Aug;39(8):1879-85.

[33] Stevanovic A, Coburn M, Menon A, Rossaint R, Heyland D, Schälte G, Werker T, Wonisch W, Kiehntopf M, Goetzenich A, Rex S, Stoppe C. The importance of intraoperative selenium blood levels on organ dysfunction in patients undergoing off-pump cardiac surgery: a randomised controlled trial. PLoS One. 2014 Aug 13;9(8):e104222.

[34] Stoppe C, Spillner J, Rossaint R, Coburn M, Schälte G, Wildenhues A, Marx G, Rex S. Selenium blood concentrations in patients undergoing elective cardiac surgery and receiving perioperative sodium selenite. Nutrition. 2013 Jan;29(1):158-65.

[35] Leong JY, van der Merwe J, Pepe S, Bailey M, Perkins A, Lymbury R, Esmore D, Marasco S, Rosenfeldt F. Perioperative metabolic therapy improves redox status and outcomes in cardiac surgery patients: a randomised trial. Heart Lung Circ. 2010 Oct;19(10):584-91.

[36] Koramaz I, Pulathan Z, Usta S, Karahan SC, Alver A, Yaris E, Kalyoncu NI, Ozcan F. Cardioprotective effect of cold-blood cardioplegia enriched with N-acetylcysteine during coronary artery bypass grafting. Ann Thorac Surg. 2006 Feb;81(2):613-8.

[37] Peker O, Peker T, Erdogan D, Ozaydin M, Kapan S, Sutcu R, Ibrisim E. Effects of intravenous N-acetylcysteine on periprocedural myocardial injury after on-pump coronary artery by-pass grafting. J Cardiovasc Surg (Torino). 2008 Aug;49(4):527-31.

[38] Adabag AS, Ishani A, Bloomfield HE, Ngo AK, Wilt TJ. Efficacy of N-acetylcysteine in preventing renal injury after heart surgery: a systematic review of randomized trials. Eur Heart J. 2009 Aug;30(15):1910-7.

[39] Gu WJ, Wu ZJ, Wang PF, Aung LH, Yin RX. N-Acetylcysteine supplementation for the prevention of atrial fibrillation after cardiac surgery: a meta-analysis of eight randomized controlled trials. BMC Cardiovasc Disord. 2012 Feb 24;12:10.

[40] Wang G, Bainbridge D, Martin J, Cheng D. N-acetylcysteine in cardiac surgery: do the benefits outweigh the risks? A meta-analytic reappraisal. J Cardiothorac Vasc Anesth. 2011 Apr;25(2):268-75.

[41] Martin CM, Doig GS, Heyland DK, Morrison T, Sibbald WJ; Southwestern Ontario Critical Care Research Network. Multicentre, cluster-randomized clinical trial of algorithms for critical-care enteral and parenteral therapy (ACCEPT). CMAJ. 2004 Jan 20;170(2):197-204.

[42] Jain MK, Heyland D, Dhaliwal R, Day AG, Drover J, Keefe L, Gelula M. Dissemination of the Canadian clinical practice guidelines for nutrition support: results of a cluster randomized controlled trial. Crit Care Med. 2006 Sep;34(9):2362-9.

[43] Heyland DK, Murch L, Cahill N, McCall M, Muscedere J, Stelfox HT, Bray T, Tanguay T, Jiang X, Day AG. Enhanced protein-energy provision via the enteral route feeding protocol in critically ill patients: results of a cluster randomized trial. Crit Care Med. 2013 Dec;41(12):2743-53.

[44] Kafai MR, Ganji V. Sex, age, geographical location, smoking, and alcohol consumption influence serum selenium concentrations in the USA: third National Health and Nutrition Examination Survey, 1988-1994. J Trace Elem Med Biol. 2003;17(1):13-8.

[45] Morris JS, Crane SB. Selenium toxicity from a misformulated dietary supplement, adverse health effects, and the temporal response in the nail biologic monitor. Nutrients. 2013 Mar 28;5(4):1024-57.

[46] Nuttall KL. Evaluating selenium poisoning. Ann Clin Lab Sci. 2006 Autumn;36(4):409-20.

[47] Krittaphol W, McDowell A, Thomson CD, Mikov M, and Fawcett JP. Biotransformation of L: Selenomethionine and selenite in rat gut contents. Biol Trace Elem Res 2010 [Epub ahead of print].

[48] Ohta Y and Suzuki KT. Methylation and demethylation of intermediates selenide and methylselenol in the metabo- lism of selenium. Toxicol Appl Pharmacol 226: 169–177, 2008.

[49] Edwards P, Roberts I, Clarke M, DiGuiseppi C, Pratap S, Wentz R, Kwan I. Increasing response rates to postal questionnaires: systematic review. BMJ. 2002 May 18;324(7347):1183.

[50] Hough RL, Tarke H, Renker V, Shields P, Glatstein J. Recruitment and retention of homeless mentally ill participants in research. J Consult Clin Psychol. 1996 Oct;64(5):881-91.

[51] Roques F, Michel P, Goldstone AR, Nashef SAM. The logistic EuroSCORE. Eur Heart J. 2003 May;24(9):882-39.

[52] McDonald CI, Fung YL, Fraser JF. Antioxidant trace element reduction in an in vitro cardiopulmonary bypass circuit. ASAIO J. 2012 May-Jun;58(3):217-22.

[53] Koszta G, Kacska Z, Szatmári K, Szerafin T, Fülesdi B. Lower whole blood selenium level is associated with higher operative risk and mortality following cardiac surgery. J Anesth. 2012 Dec;26(6):812-21.

[54] Silberman S, Bitran D, Fink D, Tauber R, Merin O. Very prolonged stay in the intensive care unit after cardiac operations: early results and late survival. Ann Thorac Surg. 2013 Jul;96(1):15-22.

[55] Benstoem C, Moza A, Autschbach R, Stoppe C, Goetzenich A. Evaluating outcomes used in cardiothoracic surgery interventional research: a systematic review of reviews to develop a core outcome set. PLoS One. 2015 Apr 1;10(4):e0122204.

[56] Ferreira-González I, Busse JW, Heels-Ansdell D, Montori VM, Akl EA, Bryant DM, Alonso-Coello P, Alonso J, Worster A, Upadhye S, Jaeschke R, Schünemann HJ, Permanyer-Miralda G, Pacheco-Huergo V, Domingo-Salvany A, Wu P, Mills EJ, Guyatt GH. Problems with use of composite end points in cardiovascular trials: systematic review of randomised controlled trials. BMJ. 2007 Apr 14;334(7597):786.

[57] Poolman RW, Swiontkowski MF, Fairbank JC, Schemitsch EH, Sprague S, de Vet HC. Outcome instruments: rationale for their use. J Bone Joint Surg Am. 2009 May;91 Suppl 3:41-9.

[58] Falcoz PE, Chocron S, Stoica L, Kaili D, Puyraveau M, Mercier M, Etievent JP. Open heart surgery: one-year self-assessment of quality of life and functional outcome. Ann Thorac Surg. 2003 Nov;76(5):1598-604.

[59] Heyland DK, Muscedere J, Drover J, Jiang X, Day AG; Canadian Critical Care Trials Group. Persistent organ dysfunction plus death: a novel, composite outcome measure for critical care trials. Crit Care. 2011;15(2):R98.

[60] Stoppe C, McDonald B, Benstoem C, Elke G, Meybohm P, Whitlock R, Fremes S, Fowler R, Lamarche Y, Jiang X., Day AG, Heyland DK. Evaluation of persistent organ dysfunction plus death as a novel composite outcome in cardiac surgical patients. J Vasc Anesth 2015, *in press*, doi:10.1053/j.jvca.2015.07.035

[61] Williams MR, Wellner RB, Hartnett EA, Thornton B, Kavarana MN, Mahapatra R, Oz MC, Sladen R. Long-term survival and quality of life in cardiac surgical patients with prolonged intensive care unit length of stay. Ann Thorac Surg. 2002 May;73(5):1472-8.

[62] Vincent JL, de Mendonça A, Cantraine F, Moreno R, Takala J, Suter PM, Sprung CL, Colardyn F, Blecher S. Use of the SOFA score to assess the incidence of organ dysfunction/failure in intensive care units: results of a multicenter, prospective study. Working group on "sepsis-related problems" of the European Society of Intensive Care Medicine. Crit Care Med. 1998 Nov;26(11):1793-800.

[63] Doerr F, Badreldin AM, Heldwein MB, Bossert T, Richter M, Lehmann T, Bayer O, Hekmat K. A comparative study of four intensive care outcome prediction models in cardiac surgery patients. J Cardiothorac Surg. 2011 Mar 1;6:21.

[64] Ely EW, Margolin R, Francis J, May L, Truman B, Dittus R, Speroff T, Gautam S, Bernard GR, Inouye SK. Evaluation of delirium in critically ill patients: validation of the Confusion Assessment Method for the Intensive Care Unit (CAM-ICU). Crit Care Med. 2001 Jul;29(7):1370-9.

[65] Meplan C, Nicol F, Burtle BT, Crosley LK, Arthur JR, Mathers JC, and Hesketh JE. Relative abundance of sele- noprotein P isoforms in human plasma depends on geno- type, se intake, and cancer status. Antioxid Redox Signal 11: 2631–2640, 2009

[66] Burk RF and Hill KE. Selenoprotein P-expression, func- tions, and roles in mammals. Biochim Biophys Acta 1790: 1441–1447, 2009.

[67] Brazier J, Roberts J, Deverill M. The estimation of a preference-based measure of health from the SF-36. J Health Econ. 2002 Mar;21(2):271-92.

[68] Manca A, Hawkins N, Sculpher MJ. Estimating mean QALYs in trial-based cost-effectiveness analysis: the importance of controlling for baseline utility. Health Econ. 2005 May;14(5):487-96.

[69] Meybohm P, Bein B, Brosteanu O, Cremer J, Gruenewald M, Stoppe C, Coburn M, Schaelte G, Böning A, Niemann B, Roesner J, Kletzin F, Strouhal U, Reyher C, Laufenberg-Feldmann R, Ferner M, Brandes IF, Bauer M, Stehr SN, Kortgen A, Wittmann M, Baumgarten G, Meyer-Treschan T, Kienbaum P, Heringlake M, Schön J, Sander M, Treskatsch S, Smul T, Wolwender E, Schilling T, Fuernau G, Hasenclever D, Zacharowski K; RIPHeart Study Collaborators. A Multicenter Trial of Remote Ischemic Preconditioning for Heart Surgery. N Engl J Med. 2015 Oct 8;373(15):1397-407.

[70] Schoenfeld DA, Bernard GR; ARDS Network. Statistical evaluation of ventilator-free days as an efficacy measure in clinical trials of treatments for acute respiratory distress syndrome. Crit Care Med. 2002 Aug;30(8):1772-7.

[71] National Heart, Lung, and Blood Institute Acute Respiratory Distress Syndrome (ARDS) Clinical Trials Network, Rice TW, Wheeler AP, Thompson BT, Steingrub J, Hite RD, Moss M, Morris A, Dong N, Rock P. Initial trophic vs full enteral feeding in patients with acute lung injury: the EDEN randomized trial. JAMA. 2012 Feb 22;307(8):795-803.

[72] Rice TW, Wheeler AP, Thompson BT, deBoisblanc BP, Steingrub J, Rock P; NIH NHLBI Acute Respiratory Distress Syndrome Network of Investigators. Enteral omega-3 fatty acid, gamma-linolenic acid, and antioxidant supplementation in acute lung injury. JAMA. 2011 Oct 12;306(14):1574-81.

[73] Kahan BC. Accounting for centre-effects in multicentre trials with a binary outcome - when, why, and how? BMC Medical Research Methodology 2014;14.

[74] Hedeker D, Gibbons RD. Application of Random-Effects Pattern-Mixture Models for Missing Data in Longitudinal Studies. Psychological Methods 1997;2:64-78.

[75] Fairclough DL. Design and Analysis of Quality of Life Studies in Clinical Trials. Boca Raton Chapman & Hall/CRC 2002.

[76] Bagshaw SM, Stelfox HT, McDermid RC, Rolfson DB, Tsuyuki RT, Baig N, Artiuch B, Ibrahim Q, Stollery DE, Rokosh E, Majumdar SR. Association between frailty and short- and long-term outcomes among critically ill patients: a multicentre prospective cohort study. CMAJ. 2014 Feb 4;186(2):E95-102.

[77] de Jong N, Gibson RS, Thomson CD, Ferguson EL, McKenzie JE, Green TJ, and Horwath CC. Selenium and zinc status are suboptimal in a sample of older New Zealand women in a community-based study. J Nutr 131: 2677–2684, 2001.

[78] Olivieri O, Stanzial AM, Girelli D, Trevisan MT, Guarini P, Terzi M, Caffi S, Fontana F, Casaril M, Ferrari S, et al. Se- lenium status, fatty acids, vitamins A and E, and aging: the Nove Study. Am J Clin Nutr 60: 510–517, 1994.

[79] Johnson CC, Fordyce FM, Rayman MP. Symposium on 'Geographical and geological influences on nutrition': Factors controlling the distribution of selenium in the environment and their impact on health and nutrition. Proc Nutr Soc. 2010 Feb;69(1):119-32.

[80] Moher D, Hopewell S, Schulz KF, Montori V, Gøtzsche PC, Devereaux PJ, Elbourne D, Egger M, Altman DG; Consolidated Standards of Reporting Trials Group. CONSORT 2010 Explanation and Elaboration: Updated guidelines for reporting parallel group randomised trials. J Clin Epidemiol. 2010 Aug;63(8):e1-37.

[81] Hausenloy DJ, Candilio L, Evans R, Ariti C, Jenkins DP, Kolvekar S, Knight R, Kunst G, Laing C, Nicholas J, Pepper J, Robertson S, Xenou M, Clayton T, Yellon DM, ERICCA Trial Investigators: Remote Ischemic Preconditioning and Outcomes of Cardiac Surgery. N Engl J Med 2015; 373:1408 17

[82] Claudia Fiorina et al., "The 6-Min Walking Test Early After Cardiac Surgery. Reference Values and the Effects of Rehabilitation Programme.," *European Journal of Cardio-Thoracic Surgery : Official Journal of the European Association for Cardio-Thoracic Surgery* 32, no. 5 (November 2007): 724–29, doi:10.1016/j.ejcts.2007.08.013.

[83] Deschka H, Schreier R, El-Ayoubi L, Erler S, Müller D, Alken A, Wimmer-Greinecker G. Prolonged intensive care treatment of octogenarians after cardiac surgery: a reasonable economic burden? Interact Cardiovasc Thorac Surg. 2013 Sep;17(3):501-6.

[84] Soppa G, Woodford C, Yates M, Shetty R, Moore M, Valencia O, Fletcher N, Jahangiri M. Functional status and survival after prolonged intensive care unit stay following cardiac surgery. Interact Cardiovasc Thorac Surg. 2013 Jun;16(6):750-4.

[85] Lagercrantz E, Lindblom D, Sartipy U. Survival and quality of life in cardiac surgery patients with prolonged intensive care. Ann Thorac Surg. 2010 Feb;89(2):490-5.

[86] Gaudino M, Girola F, Piscitelli M, Martinelli L, Anselmi A, Della Vella C, Schiavello R, Possati G. Long-term survival and quality of life of patients with prolonged postoperative intensive care unit stay: unmasking an apparent success. J Thorac Cardiovasc Surg. 2007 Aug;134(2):465-9.

[87] Gersbach P, Tevaearai H, Revelly JP, Bize P, Chioléro R, von Segesser LK. Are there accurate predictors of long-term vital and functional outcomes in cardiac surgical patients requiring prolonged intensive care? Eur J Cardiothorac Surg. 2006 Apr;29(4):466-72.

[88] Hellgren L, Ståhle E. Quality of life after heart valve surgery with prolonged intensive care. Ann Thorac Surg. 2005 Nov;80(5):1693-8.

[89] Ware JE. The SF-36 health survey. In Quality of Life and Pharmacoeconomics in Clinical Trials, edited by B. Spilker. Lippincott-Raven Publishers, Philadelphia, 1996.

[90] Essink-Bot ML, Krabbe PF, Bonsel GJ, Aaronson NK. An empirical comparison of four generic health status measures. The Nottingham Health Profile, the Medical Outcomes Study 36-item Short-Form Health Survey, the COOP/WONCA charts, and the EuroQol instrument. Med Care. 1997 May;35(5):522-37.

[91] Koch CG, Khandwala F, Blackstone EH. Health-related quality of life after cardiac surgery. Semin Cardiothorac Vasc Anesth. 2008 Sep;12(3):203-17.

[92] Heyland DK, Hopman W, Coo H, Tranmer J, McColl MA. Long-term health-related quality of life in survivors of sepsis. Short Form 36: a valid and reliable measure of health-related quality of life. Crit Care Med. 2000 Nov;28(11):3599-605.

[93] Järvinen O, Saarinen T, Julkunen J, Huhtala H, Tarkka MR. Changes in health-related quality of life and functional capacity following coronary artery bypass graft surgery. Eur J Cardiothorac Surg. 2003 Nov;24(5):750-6.

[94] Heyland DK, Groll D, Caeser M. Survivors of acute respiratory distress syndrome: relationship between pulmonary dysfunction and long-term health-related quality of life. Crit Care Med. 2005 Jul;33(7):1549-56.

SodiUm SeleniTe Administration IN Cardiac Surgery (SUSTAIN CSX – Trial). A Multicentre Randomized Controlled Trial of High Dose Sodium-selenite Administration in High Risk Cardiac Surgical Patients

NCT Number: 02002247

Version: 27-May 2021

Statistical Analysis Plan for: The SUSTAIN-CSX Trial

1 Administrative Information

1.1 SAP Summary Table

TRIAL FULL TITLE	SodiUm SeleniTe Adminstration IN Cardiac Surgery (SUSTAIN CSX®-trial). A multicenter, randomized controlled trial of high dose sodium-selenite administration in high risk cardiac surgical patients
TRIAL REGISTRATION	https://clinicaltrials.gov/ct2/show/NCT02002247
PROTOCOL PUBLICATION	NONE
CURRENT PROTOCOL DATE	2018-08-01
TRIAL PRINCIPAL INVESTIGATOR	Daren K. Heyland
TRIAL SENIOR STATISTICIAN	Andrew G. Day
TRIAL COORDINATOR	John Clarke
STATISTICIAN(S) PERFORMING ANALYSIS	Xuran Jiang and Andrew G. Day
SAP AUTHOR(s)	Andrew G. Day, Xuran Jiang and John Clarke
SAP DATE	2021-05-27
SAP STATUS	Version 1.- Finalized and approved.
SAP REVISION HISTORY	None yet
STATUS OF TRIAL AT TIME OF SAP FINALIZATION OF V1.0	Enrollment completed. Blinded data cleaning completed. No by arm outcome results generated yet.

1.2 Signatures

I have read and approve the enclosed SAP dated 2021-05-27 for the Sustain CSX trial.

Senior Statistician & SAP Author

Name: Andrew G. Day

Signature: ______________________

Date: ______________________

Statistician Performing Analysis (other than senior statistician):

Name: Xuran Jiang

Signature: ______________________

Date: ______________________

Trial Co-ordinator

Name: John Clarke

Signature: ______________________

Date: ______________________

Principal Investigator

Name: Daren K. Heyland

Signature: ______________________

Date: ______________________

Statistical Analysis Plan SUSTAIN CSX Trial Clinical Evaluation Research Unit

1.2 Signatures

I have read and approve the enclosed SAP dated 2021-05-27 for the Sustain CSX trial.

Senior Statistician & SAP Author

Name: Andrew G. Day

Signature:

Date: May 27, 2021

Statistician Performing Analysis (other than senior statistician):

Name: Xuran Jiang

Signature:

Date: 27/05/21

Trial Co-ordinator

Name: John Clarke

Signature:

Date: May 27th, 2021

Principal Investigator

Name: Daren K. Heyland

Signature:

Date: May 27/21

1.3 Purpose, usage, and target audience of this document

This document provides a detail description of the analysis plan for the SUSTAIN CSX trial. This document is meant to be used in conjunction with the study protocol. This document does not subsume the protocol, but several elements of the protocol, such as the sample size justification are reproduced herein for completeness. This document has the following purposes:

1. Provides a written agreement between the principle investigator, sponsor, lead study statistician and data analysts regarding exactly what analysis will be performed.
2. Provides a record of the analysis plan specified prior to examining any outcomes by arm.
3. Provide clear specifications for the analyst(s) performing the data filtering/transformation, variable derivations, statistical analyses and report generation.

This document follows the guidance published in JAMA by Gamble et al (2017) and referenced at https://www.equator-network.org/reporting-guidelines/guidelines-for-the-content-of-statistical-analysis-plans-in-clinical-trials/ [1] The SAP checklist is completed in Appendix A.

1.4 SAP Contributors and Signatories

4. Andrew Day drafted the SAP, Xuran Jiang contributed details regarding the definition of several outcomes, John Clarke added details regarding the trial operation and data management, and Daren Heyland helped interpret the protocol and prioritize outcomes, analyses, and validation. All authors provided critical review and editing to all parts of the SAP. The finalized version of the SAP was approved and signed off by all authors.

2 Contents

1 Administrative Information 2
1.1 SAP Summary Table 2
1.2 Signatures 3
1.3 Purpose, usage, and target audience of this document 5
1.4 SAP Contributors and Signatories 5
2 Contents 6
3 Introduction to Study 8
3.1 Background and Rationale 8
3.2 Overall Aim 8
3.3 Study Hypotheses 8
4 Study Methods 8
4.1 Trial Design 8
4.2 Randomization 8
4.3 Sample Size Considerations 9
4.4 Framework 9
4.5 Statically Interim Analysis 10
4.6 Timing of Final Analysis 10
4.7 Timing of outcome assessments 10
5 Statistical Principals 10
5.1 Confidence intervals and P-values 10
5.2 Analysis populations 10
5.3 Eligibility Criteria: 10
5.4 Screening, recruitment, patient flow/follow-up 10
5.5 Baseline Characteristics 10
6 Analysis 11
6.1 Outcome Definitions 11
6.1.1 Primary Outcome: 11
6.1.2 Secondary Outcomes: 12
6.1.3 Additional Outcomes: 14

6.1.4 Remaining study variables: 15
6.1.5 Cost Utility Analysis: 15
6.2 Analysis Methods 16
6.2.1 Primary outcome 16
6.2.2 Secondary outcomes 16
6.2.3 Additional outcomes: 17
6.2.4 Adjustment for covariates 17
6.2.5 Assumption checking 17
6.2.6 Subgroup analysis 17
6.3 Missing Data 18
6.4 Additional analysis 18
6.5 Statistical Software 18
7 Quality assurance 18
7.1 Data quality 18
7.2 Validation of SAS database and analysis 19
8 References 19
9 Appendix A: Statistical Analysis Plan (SAP) Checklist v 1.0 2019 21

3 Introduction to Study

3.1 Background and Rationale

Copied from https://clinicaltrials.gov/ct2/show/NCT02002247

> Over a million patients undergo open heart surgery annually and this number is likely to accelerate as the population ages and the prevalence of diabetes and cardiovascular disease continue to increase. Unfortunately, death, organ failure, and other serious complications are all too frequent following open heart surgery, especially in some high-risk patient populations.
>
> Selenium is a trace element that is important for many of the body's regulatory and metabolic functions especially during times of stress. International members of the study team have shown in a non-randomized study that high dose selenium supplementation was associated with improved clinical outcomes compared to a historical control group. The next step in this program of research is to conduct a randomized trial.

3.2 Overall Aim

The aim of this trial is to investigate the effects of perioperative high dose selenium supplementation in high-risk cardiac surgical patients undergoing complicated open heart surgery. If the hypothesis is proven true, and this simple, inexpensive nutrient reduces complications and improves recovery of patients undergoing cardiac surgery, there is the potential to dramatically change clinical practice and improve health outcomes.

3.3 Study Hypotheses

Perioperative high dose selenium supplementation in high-risk cardiac surgical patients undergoing complicated open heart surgery will led to better outcomes including lower mortality and fewer days requiring life sustaining therapies.

4 Study Methods

4.1 Trial Design

A randomized, placebo-controlled, double-blind, multicentre definitive trial of 1400 patients across 20 sites in Germany and Canada, which will include the pilot study patients. An industry partner (Biosyn) will provide the product and some additional support for the European sites. Patients will be randomized to receive either a daily perioperative high-dose selenium or placebo until postoperative day 10 (maximum) or upon earlier discharge from ICU.

4.2 Randomization

Randomization description copied from published protocol.

> At each participating center the local coordinating investigator will screen daily all cardiac surgical patients scheduled to undergo cardiac surgery in the near future or on the next day. A screening

log will be kept at each site to determine the number of patients meeting the inclusion criteria, those truly eligible patients, those who consent and are randomized and reasons why potentially eligible patients did not get enrolled. Following a full explanation of the nature and purpose of the study, a written informed consent will be obtained from the patients participating in the study. At the time of enrolment into the study, patients will be randomized to receive either selenium or a matching placebo similar in appearance, consistency, volume, and smell so as to blind patients, investigators and health care practitioners as to the nature of the study medication. Patients will be consecutively randomized by a web-based randomization system (concealed and blinded) developed by the Clinical Evaluation Research Unit at the Kingston Health Sciences Centre and randomization will be stratified according to centre. Randomization will be based on the method of permutated blocks of undisclosed random size stratified by centre.

4.3 Sample Size Considerations

Sample size copied from published protocol.

The distribution of the control arm was calculated based on a database of patients that underwent cardiac surgery during a 12 month follow up at Aachen University (n = 1127) and would met the inclusion criteria of the current study (n = 170). In our dataset, the mean (SD) POD free days was 23.2 (9.2); 4% of the patients died, 6% survived on life-sustaining therapy with 0 POD free days. We checked these numbers with data from the University of Ottawa Heart Institute (Canada), and numbers are consistent with the 4% 30-day mortality rate and the 6% not yet free from life-sustaining therapy by 30 days. We used simulation to estimate the power of applying the Wilcoxon rank-sum test to our primary outcome of PODs free days. The intervention arm was then generated by multiplying the control arm daily rate of liberation from life sustaining therapies by a fixed factor (hazard ratio) but assuming the same 4% mortality rate. The mean days on life-sustaining therapy was then subtracted from 30 to obtain the free days. 10.000 samples were simulated so the power estimate has more than a 95% chance of being accurate to within 1%. Based on the simulation, we would require 700 patients per arm to achieve 90% power at a two-sided alpha=0.05 if the intervention caused as 20% relative increase in the daily rate of liberation from life-sustaining therapy but no change in mortality compared to the control arm. Based on our Aachen data, such an effect size would result in an earlier liberation of life-sustaining therapies and mean increase of 1.5 POD free days (from 23.2 to 24.7 days). We believe such an effect size is plausible and is in line with minimally clinically important differences accepted in other recent major trials in the ICU setting.2

4.4 Framework

This is a confirmatory (i.e. hypothesis testing) superiority RCT comparing the efficacy and safety of perioperative high dose selenium supplementation to placebo in high-risk cardiac surgical patients undergoing complicated open heart surgery.

4.5 Interim Analysis

None

4.6 Timing of Final Analysis

All outcomes will be analyzed once all data is collected and cleaned and after finalization of the analysis plan.

4.7 Timing of outcome assessments

Most outcome assessments were measured in hospital up to 30 days or at 6 months. The timing of each outcome is described with the outcome in section 6.1.

5 Statistical Principals

5.1 Confidence intervals and P-values

95% confidence will be presented for selected key outcomes. P-values will be two-sided without adjustment for multiplicity. However, interpretation of secondary outcomes will consider the multiplicity of tests. There is one pre-specified primary test of efficacy. P<0.05 will be considered statistically significant.

5.2 Analysis populations

The primary analysis will be a modified intention-to-treat including all patients to the arm they were randomized regardless of study compliance except we will exclude randomized patients who became ineligible due to not undergoing the planned surgery AND did not receive any study medication. In addition, for key efficacy outcomes, we plan a per-protocol analysis that further excludes patients who stayed less than 24 hours in the ICU or experienced an IP-related protocol violation.

5.3 Eligibility Criteria:

Published at https://clinicaltrials.gov/ct2/show/NCT02002247#eligibility.

5.4 Screening, recruitment, patient flow/follow-up

A CONSORT style flow diagram will present the numbers of patients screened and all reasons excluded prior to randomization. The table will also include the number randomized to each arm and the number used in the primary analysis in each arm with reasons for the exclusion of randomized patients.

5.5 Baseline Characteristics

Baseline characteristics will be described by arm and overall using descriptive statistics only. Categorical variables will be described as counts (%). Continuous variables will be described as mean±SD (min to

max) and/or median [Q1 to Q3]. Separate tables will be generated for pre-operative, intra-operative, and post-operative characteristics.

The following baseline patient characteristics will be described: Age, sex, ethnicity, height, weight, BMI, Unplanned weight loss in the last 3 months, Food intake in the week prior to ICU admission, Baseline SOFA, Charlson Comorbidity Index, Functional Comorbidity Index, Patients without angina however had CCS data entered, CCS Grading(among patients with angina), NYHA Classification, EuroSCORE II classification, Manuscript classification, Duration of the surgical procedure (hours), Duration of cardiopulmonary bypass (hours), Duration of aortic clamping (hours), Baseline SF-36 (all 8 domains and 2 summary scales), Baseline Barthel ADL index, Baseline Frailty Scale, Euroscore II (%), Euroscore II (%), time from hospital admission to randomization and additional variables as explicated in the analytic dictionary.

6 Analysis

6.1 Outcome Definitions

6.1.1 Primary Outcome:

Number of days alive and free of life sustaining therapy (i.e. PODS free) in the first 30 days after the day of surgery. PFDs do not include day of surgery or days prior to surgery. Randomization is always prior to surgery and usually occurs on day of surgery, but for some patients randomization occurred prior to the day of surgery. Life sustaining therapy includes any use of the following for any duration of time on the given day: mechanical ventilation, vasopressor therapy, mechanical circulatory support, continuous renal replacement therapy, or intermittent hemodialysis. Patients who die in the first 30 days after day of surgery will be assigned 0 PODS free days.

(1) **Mechanical ventilation**: Mechanical ventilation via an endotracheal tube or tracheostomy tube OR use of non-invasive mechanical ventilation (CPAP or BiPAP) will count as mechanical ventilation unless the patient routinely uses these modalities at home. A patient will be considered liberated from mechanical ventilation at the time of extubation if they remain off mechanical ventilation at least 48 hours. However, if patients are re-intubated within 48 hours, then the intervening time will not be considered free days.

(2) **Vasopressor therapy**: days with more than 2 hours of any dose of norepinephrine, epinephrine, vasopressin, Dobutamine, Milrinone or Levosimendan and >5 ug/kg/min of dopamine, or > 50 ug/minute of phenylephrine, will not be considered free days. The 48-hour rule does not apply to vasopressor therapy or renal replacement therapy.

(3) **Mechanical circulatory support**: Use of Intra-Aortic Balloon Pump (IABP) or Extra Membrane Oxygenation (ECMO) or any Left Ventricular Assist Device (LVAD, like Impella or TandemHeart, etc.) for any duration will be considered to receive Mechanical Circulatory Support on that calendar day. As with mechanical ventilation, days will not be considered free if mechanically circulatory support is re-initiated within 48 hours, but days free in the first 48 hours will count if mechanical circulatory support is re-initiated on or after 48 hours.

(4) **Renal replacement therapy**: if the calendar day is on or between the start and stop date of any renal replacement therapy then the day is not a free day.

6.1.2 Secondary Outcomes:

1. 30-Day Mortality [Time Frame: 30 days]

   Count and percentage of patients who died within 30 days from surgery.

2. Hospital Acquired Infections [Time Frame: hospitalization]

   Count and percentage of patients with definite or probable infections will be reported.
   To be evaluated up to hospital discharge.

3. Perioperative hemodynamic profile [Time Frame: post anesthetic induction, upon ICU admission and at change of nursing shift on the morning of the first postoperative day and subsequent days in the ICU]

   This includes: Heart Rate, systemic and pulmonary blood pressures, central venous pressure, cardiac output, and mixed venous blood oxygen saturation level (SvO_2).

4. Cardiovascular Complications [Time Frame: hospitalization]

   This includes: clinically significant atrial fibrillation (>1 hour), myocardial injury≥72 hours after surgery, stroke after surgery, cardiac arrest. To be assessed up to hospital discharge.

5. Duration of Mechanical Ventilation [Time Frame: hospitalization]

   Duration of mechanical ventilation will be calculated from time of admission to ICU until time of discontinuation. All times of endotracheal extubation and any subsequent re-intubation/re-extubations or tracheostomy will be recorded. A patient will be considered liberated from mechanical ventilation at the time of liberation if they subsequently remain off mechanical ventilation for at least 48 hours i.e. for a patient who is removed from mechanical ventilation and reintubated 30 hours later, the intervening days from extubation to re-intubation will be considered to represent ongoing need for life support. Use of non-invasive mechanical ventilation (CPAP or BiPAP) will count as mechanical ventilation unless the patient routinely uses these modalities at home.

To be assessed up to hospital discharge.

6. Incidence of post-operative delirium [Time Frame: hospitalization]

CAM-ICU score was assessed upon admission to ICU post-operatively (not pre-operatively, may be same day as surgery or the next day) and will be reported daily while in ICU.

The CAM (Confusion Assessment Method for the Intensive Care Unit) has four features. Delirium is diagnosed when both Feature 1 and 2 are positive, along with either Feature 3 or Feature 4

Feature 1. Acute Onset of Mental Status Changes or Fluctuating Course.
- Is there evidence of an acute change in mental status from the baseline?
- Did the (abnormal) behavior fluctuate during the past 24 hours, that is, ten to come and go or increase and decrease in severity?

Feature 2. Inattention
- Did the patient have difficulty focusing attention?
- Is there a reduced ability to maintain and shift attention?

Feature 3. Disorganized Thinking
- Was the patient's thinking disorganized or incoherent, such as rambling or irrelevant conversation, unclear or illogical flow of ideas, or unpredictable switching from subject to subject?
- Was the patient able to follow questions and commands throughout the assessment?

Feature 4. Altered Level of Consciousness
- Any level of consciousness other than 'alert'.
- Alert-normal, spontaneous fully aware of environment and interacts appropriately.
- Vigilant-hyperalert
- Lethargic-drowsy but easily aroused, unaware of some elements in the environment, or not spontaneously interacting appropriately with the interviewer; becomes fully aware and appropriately interactive when prodded minimally
- Stupor-difficult to arouse, unaware of some or all elements in the environment, or not spontaneously interacting with the interviewer; becomes incompletely aware and inappropriately interactive when prodded strongly
- Coma – unarousable, unaware of all elements in the environment, with no spontaneous interaction or awareness of the interviewer, so that the interview is difficult or impossible event with maximal prodding

7. ICU Length of stay [Time Frame: ICU stay]

ICU length of stay is calculated from time of ICU admission to time and date of actual discharge.

To be assessed up to ICU discharge.

8. Hospital Re-admission Rates [Time Frame: 6-months]

To be assessed up to 6-months post-surgery. Count and proportion of patients who were admitted to hospital more than once.

9. Hospital Length of stay [Time Frame: hospitalization]

   Hospital length of stay will be calculated from the date of index surgical procedure to time and date of discharge from hospital.

   To be assessed up to hospital discharge.

10. 6-Month Survival [Time Frame: 6-months]

    Kaplan-Meir curves of and hazard ratio of survival over 6 months starting with day of surgery.

11. Quality of Life [Time Frame: day 30, 3-months and 6-months]

    The SF-36 physical and mental summary scales will be analyzed separately.

    Barthel Index of Activities of Daily Living Total Score

    Frailty Scale

12. Return to work [Time Frame: 6-months]

    Assessed using a questionnaire to determine the patient's ability to return to their pre-operative working capabilities. To be assessed up to 6 months post-surgery.

13. 6-minute walking test [Time Frame: hospital discharge]

    This was done in a sub-study of pre specified patients.

    In the 6-minute walking distance (6MWD) was performed at hospital discharge and at 3 months. The analysis of the 6MWD is based on rank order where patient who died prior to testing are assigned the lowest rank, people who were unable due to illness or physical limitation were assigned a value of zero and people who did the test are ranked according to their distance walked. Patients not doing the test for other reasons (missed due to RC unavailable or unaware, missed due to hospital discharge, did not return to clinic, patient refused but able, or COVID-19 reasons) were excluded from the analysis. “Other” will be blindly adjudicated to determine difference from unable or otherwise missing.

6.1.3 Additional Outcomes:

1. Laboratory outcomes [Time Frame: hospital discharge]

   Values above or below certain thresholds, depending on the variable will be reported as an ‘ever’ event, per patient, per group based on establish clinical standards.

2. Days alive post hospital discharge (DAPHD6M) [Time Frame: 182 days]

This is number of days in the first 182 days post-surgery date where patients were alive and discharged from the hospital. Patients who die within 182 days can still have >0 DAPHD6M if they were alive for some days after hospital discharge. A patient who is discharged on day X (where X<182) and dies on day X+2 would be considered to have 1 DAPHD6M, because the date of discharge and the date of death are not counted as free days, but there is otherwise no minimum amount of survival required.

6.1.4 Remaining study variables:

The study analytic dictionary contains a complete list of study variables with information including their label, their REDCap location (or identified as derived), their valid values or allowable range, and the variables scale (binary, nominal, ordinal, continuous or special) which is used to determine how they will be analyzed. The complete stats report will contain the following sections reported mostly in tabular form:

1 Baseline Demographics (Pre-Procedure)
2 Baseline Demographics (Intra-operative)
3 Baseline Demographics (Immediate Post-operative)
4: Compliance with Study Investigational Product
5: Protocol Violations
6: SAEs
7: Treatment period assessment:
8: Standard Nutrition Practices
9: Events of Interest
10: Primary outcome
11: Secondary Outcomes
12: Additional Outcomes

6.1.5 Cost Utility Analysis:

A cost utility analysis will be performed if the results of this study show clear benefit of sodium-selenite administration compared to placebo. The cost effectiveness of sodium-selenite administration compared to placebo will be assessed in terms of the incremental cost per quality adjusted life year (QALY) gained from the perspective of health care system. Analysis will incorporate data on resource use and patients utility values up to 6 months post sodium-selenite administration given the assumption that no long term differences in outcome are expected. Resource use will be assessed by data collected at the follow up interviews. Utility values would be derived from SF-36 using the algorithm proposed by Brazier et al. QALYs will be estimated for each patient within the clinical trial using the total area under the curve method. The incremental cost and QALY will be estimated using a regression analysis approach. Uncertainty in the analysis will be addressed by estimating 95% CIs using a non-parametric bootstrapping method. Further details of the cost utility analysis, should it be indicated, will be detailed in a separate document.

6.2 Analysis Methods

6.2.1 Primary outcome

To test the between arm difference of PODS free days (PFDs) we will use the van Elteren test which is a stratified version of the Wilcoxon rank-sum test where the ranking is done within site to control for heterogeneity in PFDs between sites. This is a slight deviation from the original protocol which planned to use the un-stratified Wilcoxon rank-sum test. The daily proportion of patients alive and free of life sustaining therapy by arm over the first 30 days will be reported in a table or graphically. In this table or figure we will also report the daily usage rates of each specific life sustaining therapy. The key summary measure for the effect size of PFDs will be a within site concordance index (c-index). The within site c-index estimates the probability that a patient in the intervention arm will have more PFDs than a patient in the control arm from the same site. The within site c-index can range from 0 to 1, where 1 indicates that within each site every patient in the intervention arm has more PFDs than any patient in the control arm, 0 is the converse, and 0.5 would indicate no difference between arms. The within site c-index will be defined as follows: 1) within each site, compare every patient in the intervention arm to every patient in the control arm, so for example if a site had 10 patient in the intervention arm and 12 patents in the control arm there would be 120 comparisons, 2) assign each comparison a value if 1 the intervention arm patient has more PFDs, 0 if the control arm patient has more PFDS and 0.5 if both arms have the same PFDs; 3) calculate the average of within site c-indexes weighting each site proportionally to the square root of the number of comparisons within the site. We will then obtain the 2.5th and 97.5th percentile of 10, 000 bootstrap samples to estimate the 95% confidence intervals of the within site c-index.

6.2.2 Secondary outcomes

Secondary binary outcomes such as cardiovascular complications, postoperative delirium, re-admissions, hospital-acquired infections and 30-day mortality will be compared between groups by a logistic mixed effects model with site included as a random effect.[1] Odds ratios with 95% confidence intervals will be reported.

Six-month mortality will be described by group using Kaplan-Meier curves. Survival will be compared by a hazard ratio with 95% confidence intervals and corresponding Wald test. Estimates will be derived from the Cox proportional hazards model with a random frailty for site. If the proportional hazards assumption is clearly and meaningfully violated, we will report a smoothed time dependent hazard ratio over time. However, the aforementioned overall Wald test will remain the primary test of statistical significance. Patients will be censored at the earliest of 183 days post randomization or last known follow-up.

Length of ICU and hospital stay will be summarized by arm using the quartiles of time to live discharge estimated from the subdistribution cumulative incidence function (CIF) where death is treated as a competing risk precluding the possibility of discharge. The between arm difference in time to live discharge will be tested using the Wald test from the Cox proportional hazards model with site as a random frailty to account for potential between site heterogeneity. Patients who die prior to discharge will be censored after the end of the follow-up period to account for the competing risk of death. This will yield virtually the same results as the Fine and Gray approach treating death as a competing risk precluding discharge, except we will have incorporated ICU as a random effect. This outcome is also known as time-to-discharge-alive (TTDA).

The SF-36, Barthel Index and frailty scale will be collected at baseline. At 30 days, 3 months and 6 months after surgery, we will repeat the Barthel Index and SF-36 and collect return to work data. The SF-36 physical and mental summary scales and the Barthel index will be treated as continuous variables and analyzed using a linear mixed effects model for longitudinal data. In order to include all patients

with a baseline assessment regardless of follow-up, we will use constrained longitudinal data analysis where the independent dummy variable indicating treatment arm (1-treatment or 0-control) will be set so to 0 at baseline for both arms but 1 post baseline for the intervention arm.[2, 3] This model will include site as a random effect and will allow for unstructured within patient correlation as estimated by restricted maximum likelihood. The focus of these secondary analyses will be primarily descriptive as these outcomes are limited to survivors and no imputation for decedents is planned. Thus, these outcomes will not be informative of treatment efficacy if they are in the opposite direction from survival. The between group difference in the change from baseline to the various follow-up time points will be described as expected means with 95% confidence intervals.

6.2.3 Additional outcomes:

Days alive and home in 6 months will be analyzed using the same approach as PFDs.

Variables collected but not specifically listed above will be described by arm at each time they were collected using counts and percentages for categorical variables and medians and quartiles or means and standard deviations for continuous variables. Post baseline differences in these variables will be tested using the Cochran-Mantel-Haenszel test stratified by site for the categorical variables and the van Elteren test stratified by site for continuous variables. Analysis of these variables will be considered exploratory so no adjustment for multiplicity will be applied to p-values, but multiplicity of tests will be considered in when interpreting these results.

Study reporting will be in accordance with the CONSORT statement.[4]

6.2.4 Adjustment for covariates

Analysis involving hypothesis testing or creation of confidence intervals will control for site which was the sole stratification factor at randomization. No other covariates will be controlled for.

6.2.5 Assumption checking

The analysis of the primary outcome is non-parametric so no assumptions will be checked. The proportional hazards assumption of 6-month survival will be assessed graphically.

6.2.6 Subgroup analysis

A priori, we expect that there may be a heterogeneity of treatment effect amongst different patient populations. For example, older, sicker patients with less reserve may benefit the most from selenium supplementation. Thus, we plan to do a subgroup analysis comparing the treatment effect in older patients vs. younger patients (based on median age of 70), patients who are frail (Clinical Frailty Scale $\geq$4)[5] vs. those who are not, patients who are at nutrition risk (positive features of reduced oral intake or recent weight loss) vs. those that are not, in patients that undergo combined procedures (CABG+ value(s) and CABG plus ‘other’) vs. those that do not have combined procedures, patients who underwent urgent survey vs. those who underwent elective surgery, patients with moderate-severe baseline chronic kidney disease vs. those that do not, patients with a low ejection fraction (EF <39%) vs. those with EF 40 or greater, and patients with a higher vs lower Euroscore (based on the median score)

and longer vs. shorter CPB (based on median value). In support of these proposed analyses, there is an apparent decline in circulating selenium levels in the elderly in certain populations, which may occur independently of intake.[6,7] Given the potential differences in baseline selenium levels between North Americans and Europeans (due to selenium depletion in the soil in Europe),[8] we plan to compare the effect of selenium in the Canadian vs. German subpopulations. Forest plots will be provided to display the effect measure with 95% CIs within each subgroup and sites. For these subgroup analyses, we plan to examine the primary outcome and select secondary outcomes (TTDA, 6 month survival and DAPHD6M). The effect measure for the primary outcome will be the stratified c-index as described in section 6.2. Statistical tests of interaction between treatment arm and subgroup may be performed if treatment effect appears meaningfully different between subgroups.

6.3 Missing Data

The number of missing items will be presented by arm for each outcome. For the primary outcome, if >5% of patients have missing values then we will perform multiple imputation based on prior daily data and baseline characteristics to use the entire mITT population for the primary analysis of the primary outcome.

6.4 Additional analysis

The database generated from the SUSTAIN CSX trial may be used for additional secondary analyses exploring questions other than assessing the efficacy Sodium Selenite Administration in high-risk cardiac surgical patients undergoing complicated open-heart surgery. Plans for these additional secondary analyses are to be determined and are not part of the primary SUSTAIN CSX analysis.

6.5 Statistical Software

The main analysis was performed using SAS 9.4 TS level 1M2 and SAS/STAT version 14.2 under Windows 7 Professional version 6.1.7601. The independent validation of selected items (see section 8.2) was performed using the same software and operating system except SAS 9.4 was level 1M4.

7 Quality assurance

7.1 Data quality

Data was entered into REDCap by trained local site personal. Each user with access to REDCap had a unique username and password. Access to REDCap was secure and an audit trial was maintained to keep track of the username, time, and values of all data entry and modification. A custom secure randomization module was used to implement the randomization list and maintain concealment of future allocations. A custom query module was used to implement extensive value, range, logical

(including date sequence) data checks. Any violation of the pre-defined data checks triggered data queries that were tracked and required resolution (either correction or acceptance by central staff) prior to data being marked as finalized.

A touch base meeting was conducted with each site after their first patient to address any questions that may have arose in conducting the study and collecting data. Key data items from 2 patients at each site were monitored via source verification once they had randomized 2 patients. After the initial 2 patients were monitored, sites were assessed for risk and follow-up monitoring only conducted when needed. The REDCap database was downloaded and converted into a multi-table analytic SAS database. Some filtering, data transformation, and variable derivation was performed in SAS. Boxplots were generated for all continuous variables and outliers were queried; all outliers were either corrected or verified as correct.

Quality assurance reports were run periodically throughout the trial to assess the completes, timeliness, validity and quality of trial implementation and data capture by site. Issues were flagged and resolved with participating sites in real time.

7.2 Validation of SAS database and analysis

The study PI and study co-ordinary will sense check all results to make sure they are not highly suspicions and that all counts are consistent with the patient flow diagram.

A second statistician who did not perform the primary analysis will independently verify the patient flow counts and re-analyze the following key outcomes: 1) PODS free days, 3) 30-day morality, 6) six-month survival.

8 References

1. Kahan BC. Accounting for centre-effects in multicentre trials with a binary outcome - when, why, and how? BMC Medical Research Methodology 2014;14.
2. Coffman CJ, Edelman D, Woolson RF. To condition or not condition? Analysing ‘change’ in longitudinal randomised controlled trials. BMJ open 2016;6:e013096.
3. Liu GF, Lu K, Mogg R, et al. Should baseline be a covariate or dependent variable in analyses of change from baseline in clinical trials? Stat Med 2009;28:2509–30.
4. Moher D, Hopewell S, Schulz KF, Montori V, Gøtzsche PC, Devereaux PJ, Elbourne D, Egger M, Altman DG; Consolidated Standards of Reporting Trials Group. CONSORT 2010 Explanation and Elaboration: Updated guidelines for reporting parallel group randomised trials. J Clin Epidemiol. 2010 Aug;63(8):e1-37.

5. Bagshaw SM, Stelfox HT, McDermid RC, Rolfson DB, Tsuyuki RT, Baig N, Artiuch B, Ibrahim Q, Stollery DE, Rokosh E, Majumdar SR. Association between frailty and short- and long-term outcomes among critically ill patients: a multicentre prospective cohort study. CMAJ. 2014 Feb 4;186(2):E95-102.
6. de Jong N, Gibson RS, Thomson CD, Ferguson EL, McKenzie JE, Green TJ, and Horwath CC. Selenium and zinc status are suboptimal in a sample of older New Zealand women in a community-based study. J Nutr 131: 2677–2684, 2001.
7. Olivieri O, Stanzial AM, Girelli D, Trevisan MT, Guarini P, Terzi M, Caffi S, Fontana F, Casaril M, Ferrari S, et al. Se- lenium status, fatty acids, vitamins A and E, and aging: the Nove Study. Am J Clin Nutr 60: 510–517, 1994.
8. Johnson CC, Fordyce FM, Rayman MP. Symposium on 'Geographical and geological influences on nutrition': Factors controlling the distribution of selenium in the environment and their impact on health and nutrition. Proc Nutr Soc. 2010 Feb;69(1):119-32.

9 Appendix A: Statistical Analysis Plan (SAP) Checklist v 1.0 2019

Section/Item	Index	Description	Reported on page #
**Section 1: Administrative information**			
Trial and Trial registration	1a	Descriptive title that matches the protocol, with SAP either as a forerunner or subtitle, and trial acronym (if applicable)	1
	1b	Trial registration number	1
SAP Version	2	SAP version number with dates	1
Protocol Version	3	Reference to version of protocol being used	1
SAP revisions	4a	SAP revision history	1
	4b	Justification for each SAP revision	1
	4c	Timing of SAP revisions in relation to interim analyses, etc.	1
Roles and responsibility	5	Names, affiliations, and roles of SAP contributors	2
Signatures of:	6a	Person writing the SAP	1, 3
	6b	Senior statistician responsible	1
	6c	Chief investigator/clinical lead	1
**Section 2: Introduction**			
Background and rationale	7	Synopsis of trial background and rationale including a brief description of research question and brief justification for undertaking the trial	6
Objectives	8	Description of specific objectives or hypotheses	6
**Section 3: Study Methods**			
Trial design	9	Brief description of trial design including type of trial (e.g., parallel group, multi-arm, crossover, factorial) and allocation ratio and may include brief description of interventions	6
Randomization	10	Randomization details, e.g., whether any minimization or stratification	6

		occurred (including stratifying factors used or the location of that information if it is not held within the SAP)	
Sample size	11	Full sample size calculation or reference to sample size calculation in protocol (instead of replication in SAP)	7
Framework	12	Superiority, equivalence, or noninferiority hypothesis testing framework, including which comparisons will be presented on this basis	7
Statistical interim analysis and stopping guidance	13a	Information on interim analyses specifying what interim analyses will be carried out and listing of time points	8
	13b	Any planned adjustment of the significance level due to interim analysis	NA
	13c	Details of guidelines for stopping the trial early	NA
Timing of final analysis	14	Timing of final analysis, e.g., all outcomes analysed collectively or timing stratified by planned length of follow-up	8
Timing of outcome assessments	15	Time points at which the outcomes are measured including visit “windows”	8
**Section 4: Statistical Principals**			
Confidence intervals and *P* values	16	Level of statistical significance	8
	17	Description and rationale for any adjustment for multiplicity and, if so, detailing how the type 1 error is to be controlled	8
	18	Confidence intervals to be reported	8
Adherence and Protocol deviations	19a	Definition of adherence to the intervention and how this is assessed including extent of exposure	8
	19b	Description of how adherence to the intervention will be presented	13
	19c	Definition of protocol deviations for the trial	NA
	19d	Description of which protocol deviations will be summarized	NA

Analysis populations	20	Definition of analysis populations, e.g., intention to treat, per protocol, complete case, safety	8
**Section 5: Trial Population**			
Screening data	21	Reporting of screening data (if collected) to describe representativeness of trial sample	8
Eligibility	22	Summary of eligibility criteria	8
Recruitment	23	Information to be included in the CONSORT flow diagram	8
Withdrawal/ Follow-up	24a	Level of withdrawal, e.g., from intervention and/or from follow-up	8
	24b	Timing of withdrawal/lost to follow-up data	8
	24c	Reasons and details of how withdrawal/lost to follow-up data will be presented	8
Baseline patient characteristics	25a	List of baseline characteristics to be summarized	8-9
	25b	Details of how baseline characteristics will be descriptively summarized	8-9
**Section 6: Analysis**			
Outcome definitions		List and describe each primary and secondary outcome including details of:	9-13
	26a	Specification of outcomes and timings. If applicable include the order of importance of primary or key secondary end points (e.g., order in which they will be tested)	9-12
	26b	Specific measurement and units (e.g., glucose control, hbA1c [mmol/mol or %])	NA
	26c	Any calculation or transformation used to derive the outcome (e.g., change from baseline, QoL score, Time to event, logarithm, etc.)	9-13
Analysis methods	27a	What analysis method will be used and how the treatment effects will be presented	14-16
	27b	Any adjustment for covariates	15
	27c	Methods used for assumptions to be checked for statistical methods	15
	27d	Details of alternative methods to be used if distributional assumptions do not hold, e.g., normality, proportional hazards, etc.	14 (for 6-month survival)

	27e	Any planned sensitivity analyses for each outcome where applicable	14
	27f	Any planned subgroup analyses for each outcome including how subgroups are defined	15
Missing data	28	Reporting and assumptions/statistical methods to handle missing data (e.g., multiple imputation)	16
Additional analyses	29	Details of any additional statistical analyses required, e.g., complier-average causal effect10 analysis	16
Harms	30	Sufficient detail on summarizing safety data, e.g., information on severity, expectedness, and causality;<br>details of how adverse events are coded or categorized; how adverse event data will be analysed,<br>i.e., grade 3/4 only, incidence case analysis, intervention emergent analysis	NA
Statistical software	31	Details of statistical packages to be used to carry out analyses	16
References	32a	References to be provided for nonstandard statistical methods	17
	32b	Reference to Data Management Plan	16-17
	32c	Reference to the Trial Master File and Statistical Master File	13 (analytic dictionary)
	32d	Reference to other standard operating procedures or documents to be adhered to	16-17

Taken from the paper: Gamble C, Krishan A, Stocken D, Lewis S, Juszczak E, Doré C, et al. Guidelines for the Content of Statistical Analysis Plans in Clinical Trials. JAMA. 2017;318(23):2337-43.

Abbreviations: CONSORT, Consolidated Standards of Reporting Trials; hbA1c, haemoglobin A1c; QoL, quality of life; SAP, statistical analysis plan.
