# Supplementary material for: Effect of High-Dose Selenium on Postoperative Organ Dysfunction and Mortality in Cardiac Surgery Patients: The SUSTAIN CSX Randomized Clinical Trial
Source: JAMA Surg. 2023 Jan 11;158(3):235–44. doi: 10.1001/jamasurg.2022.6855 (PMC9857635; doi:10.1001/jamasurg.2022.6855)
Supplement: Supplement 2. — eMethods. eFigure 1. Intervention Scheme eFigure 2. Six-month Kaplan-Meier Survival Curve eFigure 3. Product-Limit Survival Estimates eAppendix. Supplemental Data to Figure 3A and 3B eTable 1. Compliance with Study Investigational Product eTable 2. Protocol Violations and Concomitant Administration of Antioxidants or Corticosteroids eTable 3. Components of POD by Day eTable 4. Duration of PODs Components eTable 5. Hospital Acquired Infections eTable 6. Serious Adverse Events eReferences [file jamasurg-e226855-s002.pdf]

Supplemental Online Content

Stoppe C, McDonald B, Meybohm P, et al; the SUSTAIN CSX Study Collaborators. Effect of high-dose selenium on postoperative organ dysfunction and mortality in cardiac surgery patients: the SUSTAIN CSX randomized clinical trial. *JAMA Surg*. Published online January 11, 2023. doi:10.1001/jamasurg.2022.6855

eMethods.
eFigure 1. Intervention Scheme
eFigure 2. Six-month Kaplan-Meier Survival Curve
eFigure 3. Product-Limit Survival Estimates
eAppendix. Supplemental Data to Figure 3A and 3B
eTable 1. Compliance with Study Investigational Product
eTable 2. Protocol Violations and Concomitant Administration of Antioxidants or Corticosteroids
eTable 3. Components of POD by Day
eTable 4. Duration of PODs Components
eTable 5. Hospital Acquired Infections
eTable 6. Serious Adverse Events
eReferences

This supplemental material has been provided by the authors to give readers additional information about their work.

© 2023 Stoppe C et al. *JAMA Surgery*.

eMethods.

Clinical Evaluation Research Unit (CERU)

The coordinating centre for this study is located at the Clinical Evaluation Research Unit (CERU) at the Kingston General Hospital, Ontario, Canada (see www.ceru.ca). Dr. Heyland is the Director of CERU. The mission of CERU is to improve the care of acutely ill patients through knowledge generation, synthesis, and translation in a manner that will translate into improved clinical outcomes for sick patients and improved efficiencies to our health care systems. As such, CERU consists of a staff with experience and resources to support the successful completion of all phases of the design, conduct, monitoring, and interpretation of multicentre clinical studies. CERU is staffed with several members that have considerable experience in all phases of clinical studies. The CERU took responsibility for the day-to-day conduct of the trial, including supervision of all trial staff, training and liaising with the sites, conducting site visits, arranging all trial meetings, and reporting the progress of the trial to the steering committee. The applications developer and data manager implemented the web-based data entry/query/monitoring/reporting system for efficient conduct of the trial including randomization, data validation and cleaning. The data manager worked with the statistician to undertake additional data cleaning as well as the formal analysis and reporting of the data.

For the European component of this trial, the Clinical Trial Center Aachen (CTC-A) in Germany collaborated with CERU and was responsible for the European regulatory (including BFARM) and ethics applications as well as monitoring German sites.

Participating investigators

Writing Committee

Christian Stoppe, Kenneth Christopher, Alexander Zarbock, Stephen Fremes, Gunnar Elke, Daren Heyland, and Bernard McDonald

Study Statistician

Andrew Day (Queens University, Canada).

Steering Committee

Daren Heyland, Christian Stoppe, and Bernard McDonald

Project and Data Management

Elena Laaf (Department of Anesthesiology, RWTH Aachen Germany)
Janet Overvelde (Queens University, Canada)
Margot Lemieux (Queens University, Canada)
John Clarke (Queens University, Canada)
Shawna Froese (Data Manager, Queens University, Canada)

Independent Data Monitoring and Safety Committee

Dean Fergusson
Matthew James
Richard Hall

SUSTAIN CSX – Study Collaborators*

Gregory Hare, M.D., Ph.D.[1,2], Michael WA Chu, M.D.[3], Pierre Voisine, M.D.[4], Francois Dagenais, M.D.[4], Eric Dumont, M.D.[4], Frédérique Jacques , M.D.[4], Eric Charbonneau, M.D.[4], Jean Perron, M.D.[4], Simone Lindau, M.D.[5], Roupen Hatzakorizan, M.D.[6], Assad Haneya, M.D.[7], Georg Trummer, M.D.[8], Angela Jareth, M.D.[9], Xuran Jiang, M.Sc.[10], Ellen Dresen, Ph.D.[11]

[1]Li Ka Shing Knowledge Institute, St. Michael’s Hospital, Toronto, Ontario, Canada

© 2023 Stoppe C et al. *JAMA Surgery.*

[2]Departments of Anesthesiology and Physiology, University of Toronto, Toronto, Ontario, Canada
[3]London Health Sciences Centre, London, Ontario, Canada
[4]Quebec Heart and Lung Institute, Laval University, Quebec City, QC, Canada
[5]University Hospital Frankfurt, Frankfurt am Main, Germany
[6]McGill University Health Centre, Montreal, Quebec, Canada
[7]University Hospital Schleswig-Holstein, Kiel, Germany
[8]University Heart Center Freiburg Bad Krozingen, Bad Krozingen, Germany
[9]Department of Anesthesiology and Pain Medicine, University of Toronto, Toronto, ON, Canada
[10]Clinical Evaluation Research Unit, Kingston Health Sciences Centre, Kingston, Canada
[11]Department of Anaesthesiology, Intensive Care, Emergency, and Pain Medicine, University Hospital Wuerzburg, Wuerzburg, Germany

SUSTAIN CSX – Study Investigator Group

University Hospital Aachen, Aachen, Germany; Rolf Rossaint; 133 patients

University Hospital Bonn, Bonn, Germany; Maria Wittmann; 10 patients

University Hospital Frankfurt, Frankfurt am Main, Germany; Patrick Meybohm, Simone Lindau; 106 patients

University Hospital of Giessen, Giessen, Germany; Bernd Niemann; Andreas Böning; 59 patients

University Hospital Schleswig-Holstein, Kiel, Germany; Gunnar Elke; 24 patients

University Medical Center of the Johannes Gutenberg-University Mainz, Mainz, Germany; Marion Ferner; 32 patients

University Heart Center Freiburg Bad Krozingen, Bad Krozingen, Germany; Matthias Müller; 16 patients

University Hospital Cologne, Cologne, Germany; Oliver Liakopoulos; 18 patients

Oldenburg Clinic, University of Oldenburg, Oldenburg, Germany; Ulf Günther; 102 patients

University Hospital Münster, Münster, Germany; Alexander Zarbock; 13 patients

Ludwig Maximilian University of Munich, Munich, Germany; Erich Kilger; 10 patients

University of Ottawa Heart Institute, Ottawa, Ontario, Canada; Bernard McDonald; 57 patients

Jewish General Hospital, Montreal, Quebec, Canada; Matthew Cameron; 16 patients

Sunnybrook Research Institute, Toronto, Ontario, Canada; Stephen Fremes; 39 patients

London Health Sciences Centre, London, Ontario, Canada; Philip Jones; 47 patients

St. Michael’s Hospital, Toronto, Ontario, Canada; David Mazer; 47 patients

Toronto General Hospital, Toronto, Ontario, Canada; Matteo Parotto; 76 patients

Hôpital du Sacré-Coeur de Montréal, Montreal, Quebec, Canada; Yoan Lamarche; 12 patients

Montreal Heart Institute, Montreal, Quebec, Canada; Yoan Lamarche; 27 patients

Hôpital Fleurimont (CHUS), Sherbrooke, Quebec, Canada; François Lamontagne; 20 patients

Institut Universitaire de Cardiologie et de Pneumologie de Québec – Université Laval, Laval, Quebec, Canada; Siamak Mohammadi; 182 patients

McGill University Health Centre, Montreal, Quebec, Canada; Roupen Hatzakorzian; 8 patients

Hamilton Health Sciences, Hamilton, Ontario, Canada; Richard Whitlock; 340 patients

© 2023 Stoppe C et al. *JAMA Surgery.*

Eligibility criteria

Inclusion Criteria

Adult patients (>18 years of age) scheduled to undergo elective cardiac surgery with the use of cardiopulmonary bypass (CPB) and cardioplegic arrest that exhibit a high perioperative risk profile as defined by the presence of one or more of the following:

a) Planned valve surgery combined with CABG or multiple valve replacement/repair surgeries or combined cardiac surgical procedures involving the thoracic aorta OR
b) Any cardiac surgery with a high perioperative risk profile, defined as a predicted operative mortality of ≥ 5% (EuroSCORE II)[1]

Exclusion Criteria

1) Isolated procedures (CABG only or valve only)
2) Known hypersensitivity to sodium-selenite or to any of the constituents of the solution.
3) Severe renal dysfunction as evidenced by pre-operative creatinine clearance <50 ml/min and/or severe pre-operative value of serum creatinine level above 200 micromoles/litre (local laboratory). Renal failure requiring dialysis at the point of screening.
4) Chronic liver disease as evidenced by a pre-operative total bilirubin >2 mg/dl or 34 µmol/L
5) Disabling neuropsychiatric disorders (severe dementia, severe Alzheimer's disease, advanced Parkinson's disease).
6) Inability or unwillingness of individual to give written informed consent (e.g. patients who undergo an urgent cardiac surgery)
7) Pregnancy or lactation period.
8) Simultaneous participation in another clinical trial of an experimental therapy (co enrollment acceptable in observational studies or randomized trials of existing therapies if permitted by both steering committees and local ethics boards).
9) Family members of investigators (required by German Regulatory Authorities).
10) Selenase supplementation (open-label selenium), not related to the study
11) Patients undergoing heart transplantation or preoperative planned LVAD insertion or complex congenital heart surgery.

© 2023 Stoppe C et al. *JAMA Surgery*.

Secondary endpoints

- Cardiovascular complications (e.g. arrhythmias, cardiac arrest)
- Duration of mechanical ventilation
- Incidence of postoperative delirium (assessed by CAM-ICU score[2])
- Length of stay on the intensive care unit and in hospital.
- Hospital readmission rates
- This includes: mean arterial blood pressure, cardiac power index, systemic vascular resistance
- Hospital-acquired infections (proven or suspected)
- 30-day mortality
- **Clinically significant atrial fibrillation:** Any *new* onset of atrial fibrillation OR atrial flutter that requires treatment OR persists >1h in duration. Treatment is defined electrical cardioversion or administration of IV or PO amiodarone, beta blockers or other medications. e.g. if patient has new high rate a fib and hemodynamic instability requiring cardioversion but this occurred within 20 minutes of onset, then this would still be considered significant.
- **Late Perioperative Myocardial Injury (MI) > 72h from surgery:** A late perioperative MI (i.e. > 72h from surgery) is defined as ECG changes consistent with MI (new significant Q waves in 2 contiguous leads) or evolving ST-segment or T-wave changes in 2 contiguous leads signifying ischemia or new LBBB or ST segment elevation and elevated cardiac markers (troponins or CK-MB) in the necrosis range.
- **Stroke ≥ 72h after surgery:** Defined as the presence of an acute focal neurological deficit thought to be vascular in origin with signs and symptoms lasting greater than 24h. Should be confirmed by either diagnostic imaging or a neurological assessment.
- **Cardiac arrest:** includes ventricular tachycardia, or ventricular fibrillation, or asystole/severe bradycardia, or pulseless electrical activity which results in cardiopulmonary collapse AND requires resuscitation. Resuscitation includes external OR internal cardiac massage, defibrillation, or cardioversion, or emergency resternotomy. Simply turning on epicardial pacemaker for asystole/severe bradycardia is not to be counted as cardiac arrest unless accompanied by resuscitation as defined above. Ventricular tachycardia which spontaneously resolves is not to be counted as cardiac arrest.
- Patients were contacted at 3 and 6 months post randomization to assess long-term survival and health-related quality of life.
- 6 Minute walk Test will was a further powered secondary outcome (hospital discharge and if available 3 months routine follow-up visit at clinic), which will not be reported in this manuscript.
- The patients perioperative hemodynamic profile (mean arterial blood pressure, cardiac power index, systemic vascular resistance) were as well assessed but will not be reported in this manuscript.

© 2023 Stoppe C et al. *JAMA Surgery*.

Description of the laboratory measurements

In addition, in a subset of patients, blood samples were taken to measure selenium and glutathione peroxidase levels. Serum or plasma samples were collected from the included patients at each time point before administration of the IP and subsequently prepared according to standard procedures. Samples were transferred after pseudonymization on dry ice to the analytical site in Berlin, Germany (laboratory of Lutz Schomburg, Institute for Experimental Endocrinology, Charité-Universitätsmedizin Berlin). Total selenium concentrations were determined by total reflection X-ray fluorescence (TXRF) analysis using a benchtop device (S4 TStar, Bruker Nano GmbH, Berlin, Germany), as described elsewhere[3]. Glutathione peroxidase 3 (GPX3) activity was measured by a coupled test procedure monitoring nicotinamide adenine dinucleotide phosphate (NADPH) consumption at 340 nm, essentially as described[4].

Additional details on statistical analysis

Secondary binary outcomes including 30-day mortality, hospital-acquired infections, cardiovascular complications, and postoperative delirium were compared between groups by a logistic generalized linear mixed effects model with site included as a random effect. As a sensitivity analysis, we also reported the raw unadjusted odds ratios with P-values calculated by Fisher's exact test. ICU and hospital re-admission rates are reported by arm as counts and percentages with P-values by Fisher's exact test. Continuous variables were compared between groups by the van Elteren test stratifying by site.[17] The statistical analysis was performed using SAS 9.4 TS level 1M2 and SAS/STAT version 14.2 under Windows 7 professional version 6.1.7601. A data monitoring committee periodically monitored the safety reports and other aspects of quality management related to this trial.

Time-to-event estimates of overall postoperative survival were determined using the Kaplan–Meier method. The hazard rate of mortality was compared between groups by a hazard ratio (HR) with a 95% confidence interval and a corresponding Wald test. HR estimates were derived from the Cox proportional hazards model with a random frailty for site. The viability of the proportional hazards assumption was assed visually; since the Kaplan-Meier curves were virtually identical between arms, there was no need to perform further evaluation. Patients were censored at the earliest of 183 days after surgery or last known follow-up. Length of ICU and hospital stay was summarized by arm using the quartiles of time to live discharge estimated from the subdistribution cumulative incidence function where death was treated as a competing risk precluding the possibility of discharge. The between arm difference in time to live discharge was tested using the Wald test from the Cox proportional hazards model with site as a random frailty to account for potential between site heterogeneity. As per the Fine and Gray approach, patients who died prior to discharge were censored after the end of the follow-up period to account for the competing risk of death.[5]

Additional details on subgroup analysis

A priori, we expect that there may be a heterogeneity of treatment effect amongst different patient populations. For example, older, sicker patients with less reserve may benefit the most from selenium supplementation. Thus, we plan to do a subgroup analysis comparing the treatment effect in older patients vs. younger patients (based on median age of 70), patients who are frail (Clinical Frailty Scale ≥4) vs. those who are not, patients who are at nutrition risk (positive features of reduced oral intake or recent weight loss) vs. those that are not, in patients that undergo combined procedures (CABG+ value(s) and CABG plus 'other') vs. those that do not have combined procedures, patients with moderate-severe baseline chronic kidney disease vs. those that do not, patients with a low ejection fraction (EF <39%) vs. those with EF 40 or greater, and patients with a higher vs lower Euroscore (based on the median score) and longer vs. shorter CPB (based on median value). In support of these proposed analyses, there is an apparent decline in circulating selenium levels in the elderly in certain populations, which may occur independently of intake. Given the potential differences in baseline selenium levels between North Americans and Europeans (due to selenium depletion in the soil in Europe), we plan to compare the effect of selenium in the Canadian vs. German subpopulations. Forest plots were provided to display the effect measure with 95% CIs within each subgroup and sites.

© 2023 Stoppe C et al. *JAMA Surgery.*

eTable 1: Compliance with Study Investigational Product

Characteristic	Overall (n=1394)	Selenium (n=697)	Placebo (n=697)
**Was the total volume of investigational product received?**			
Baseline	1390 (99.7%)	695 (99.7%)	695 (99.7%)
Day#0	1366 (98.1%)	682 (98.0%)	684 (98.1%)
Day#1	1340 (96.8%)	664 (96.2%)	676 (97.3%)
Day#2	748 (89.2%)	377 (89.5%)	371 (88.8%)
Day#3	548 (88.0%)	275 (87.3%)	273 (88.6%)
Day#4	413 (87.5%)	212 (87.6%)	201 (87.4%)
Day#5	321 (86.5%)	171 (89.1%)	150 (83.8%)
Day#6	248 (87.3%)	134 (91.2%)	114 (83.2%)
Day#7	190 (89.6%)	102 (87.9%)	88 (91.7%)
Day#8	150 (88.2%)	77 (88.5%)	73 (88.0%)
Day#9	125 (90.6%)	66 (91.7%)	59 (89.4%)
Day#10	106 (85.5%)	56 (88.9%)	50 (82.0%)
**Was a partial volume received?**			
Day#0	3 (0.2%)	1 (0.1%)	2 (0.3%)
Day#1	1 (0.1%)	0 (0.0%)	1 (0.1%)
Day#2	2 (0.2%)	1 (0.2%)	1 (0.2%)
**Proportion of days with IP received (%) (baseline + day 0-10)**	97.7±9.7 (8.3-100.0)	97.7±9.9 (8.3-100.0)	97.6±9.5 (8.3-100.0)
**Proportion of days with missed doses (%) (baseline + day 0-10)**	5.6±12.9 (0.0-91.7)	5.7±13.2 (0.0-91.7)	5.6±12.6 (0.0- 91.7)
**Proportion of days with partial doses (%) (baseline + day 0-10)**	0.1±1.8 (0.0-33.3)	0.1±1.6 (0.0-33.3)	0.2±2.1 (0.0-33.3)

© 2023 Stoppe C et al. *JAMA Surgery*.

eTable 2: Protocol Violations and Concomitant Administration of Antioxidants or Corticosteroids

Characteristic	Overall (n=1394)	Selenium (n=697)	Placebo (n=697)
**Ineligible patient enrolled**	3 (0.2%)	2 (0.3%)	1 (0.1%)
**Antioxidants**	**24**	**13**	**11**
Vitamin C	8 (33.3%)	4 (30.8%)	4 (36.4%)
Vitamin E	1 (4.2%)	1 (7.7%)	0 (0.0%)
N-acetyl cysteine	17 (70.8%)	9 (69.2%)	8 (72.7%)
**Corticosteroids**			
Any	56 (4.0%)	29 (4.2%)	27 (3.9%)
IV Dexamethasone sodium phosphate	4 (7.1%)	2 (6.9%)	2 (7.4%)
IV Hydrocortisone	44 (78.6%)	24 (82.8%)	20 (74.1%)
PO Hydrocortisone	3 (5.4%)	0 (0.0%)	3 (11.1%)
IV Methylprednisolone sodium succinate	3 (5.4%)	2 (6.9%)	1 (3.7%)
PO Prednisolone	1 (1.8%)	1 (3.4%)	0 (0.0%)
PO Prednisone	1 (1.8%)	0 (0.0%)	1 (3.7%)

© 2023 Stoppe C et al. *JAMA Surgery*.

eTable 3: Components of POD by Day

ICU day	Overall N = 1394					Selenium (n=697)	Placebo (n=697)
	Requiring vasopressors or inotropic support	Requiring mechanical assist device	On dialysis	Mechanically ventilated	Dead	POD + Death	POD + Death
	728	34	32	614	5	428 (61.4%)	444 (63.7%)
1	374	24	37	183	13	220 (31.6%)	214 (30.7%)
2	247	19	39	145	15	156 (22.4%)	144 (20.7%)
3	172	14	41	125	15	126 (18.1%)	105 (15.1%)
4	130	12	36	109	18	101 (14.5%)	86 (12.3%)
5	93	12	33	94	23	85 (12.2%)	72 (10.3%)
6	77	12	30	77	27	72 (10.3%)	63 (9.0%)
7	62	9	28	70	30	59 (8.5%)	63 (9.0%)
8	50	7	29	63	32	54 (7.7%)	61 (8.8%)
9	48	4	28	57	35	53 (7.6%)	56 (8.0%)
10							
	25	3	20	42	43	47 (6.7%)	47 (6.7%)
14							
	7	1	9	17	64	43 (6.2%)	43 (6.2%)
30							

ICU Intensive care Unit; POD Persistent Organ dysfunctions + Death

© 2023 Stoppe C et al. *JAMA Surgery*.

eTable 4: Duration of PODs Components

Secondary Outcome	Overall (n=1394)	Selenium (n=697)	Placebo (n=697)	Mean Difference (95% Confidence Intervals)	P-value*
**Days on mechanical ventilation**	1.6±4.9 0.0 [0.0, 1.0]	1.6±4.7 0.0 [0.0, 1.0]	1.6±5.1 0.0 [0.0, 1.0]	0.05 (-0.36, 0.46)	0.80
**Days on vasopressors or inotropic support**	1.7±3.7 1.0 [0.0, 2.0]	1.8±3.8 1.0 [0.0, 2.0]	1.6±3.6 1.0 [0.0, 2.0]	0.13 (-0.16, 0.43)	0.95
**Days on Mechanical assist device**	0.1±1.3 0.0 [0.0, 0.0]	0.1±1.0 0.0 [0.0, 0.0]	0.2±1.5 0.0 [0.0, 0.0]	-0.04 (-0.14, 0.05)	0.48
**Days on Dialysis**	0.6±3.9 0.0 [0.0, 0.0]	0.7±4.8 0.0 [0.0, 0.0]	0.4±2.7 0.0 [0.0, 0.0]	0.32 (-0.12, 0.76)	0.35

For this analysis anyone who died on mechanical ventilation/vasopressor/mechanical assist device/dialysis was considered to have stopped mechanical ventilation//vasopressor/mechanical assist device/dialysis at time of death.

mean±SD median[q,q3] was reported.

***** van Elteren test stratified by site. Mean differences were estimated by GEE allowing for clustering by site.

© 2023 Stoppe C et al. *JAMA Surgery*.

eTable 5: Hospital Acquired Infections

Secondary Outcome	Overall (n=1394)	Selenium (n=697)	Placebo (n=697)	OR from GLIMMIX model (95% Confidence Intervals)	P- values (GLIMMIX)	Raw OR (95% Confidence Intervals)	P- values (Fisher's Exact test)
Total Number of patients with Hospital-acquired infections (a patient may have multiple infections)	120 (8.6%)	62 (8.9%)	58 (8.3%)	1.07 (0.73, 1.56)	0.72	1.08 (0.74, 1.56)	0.77
Bloodstream infection	27 (1.9%)	14 (2.0%)	13 (1.9%)	1.08 (0.50, 2.31)	0.85	1.08 (0.50, 2.31)	1.00
ICU acquired pneumonia	9 (0.6%)	8 (1.1%)	1 (0.1%)	8.18 (1.01, 66.01)	0.05	8.08 (1.01, 64.75)	0.04
Lower respiratory tract infection)	2 (0.1%)	0 (0.0%)	2 (0.3%)	n/a	n/a	n/a	0.25
Intra-abdominal infection	5 (0.4%)	2 (0.3%)	3 (0.4%)	0.67 (0.11, 4.00)	0.66	0.67 (0.11, 4.00)	1.00
Urinary tract infection (UTI)	16 (1.1%)	7 (1.0%)	9 (1.3%)	n/a	n/a	0.78 (0.29, 2.09)	0.80
Surgical site infection	11 (0.8%)	5 (0.7%)	6 (0.9%)	0.83 (0.25, 2.75)	0.76	0.83 (0.25, 2.74)	1.00
Endocarditis	3 (0.2%)	1 (0.1%)	2 (0.3%)	0.51 (0.05, 5.63)	0.58	0.50 (0.05, 5.56)	1.00
Infectious colitis	2 (0.1%)	2 (0.3%)	0 (0.0%)	n/a	n/a	n/a	0.25
** Other infection*	10 (0.7%)	6 (0.9%)	4 (0.6%)	1.51 (0.42, 5.39)	0.53	1.50 (0.42, 5.35)	0.75
% of patients with definite/probable infection	58 (4.2%)	32 (4.6%)	26 (3.7%)	1.25 (0.73, 2.13)	0.42	1.24 (0.73, 2.11)	0.50
Total number of definite/probable infections	0.0±0.2 (0.0-2.0)	0.1±0.3 (0.0-2.0)	0.0±0.2 (0.0-2.0)	0.01 (-0.01, 0.04)	0.36		

***Other infection reported:** (1) Subclavian Line infection; (2) On GI scope found to have esophageal candidiasis Rx antibiotics; (3) Pneumonia by COPD elevated Temperature; (4) decubitus ulcer; (5) MSRA in nose/Pharynx.; (6) suspected viral bronchitis, nothing special was done. Patient had some fever and dyspnea; (7) increased infection Parameters unknown etiology. Blood cultures, Urine sample and ;chest radiograph did not show any results; (8) infection of central venous catheter with Staphylococcus epidermidis;(9) empirically antibiotic with tazobac, CRP and WBC increase; (10) endocarditis positive blood culture from 2017-05-31

© 2023 Stoppe C et al. *JAMA Surgery*.

eTable 6: Serious Adverse Events

Coded Category	Coded Subcategory	Overall (n=1394)	Selenium (n=697)	Placebo (n=697)	P-values
**Any Category**	*Patients with any SAE	117	57	60	**0.85
**Blood and lymphatic system disorders**	Any event within class	4	2	2	
	Anemia	1	0	1	
	Disseminated intravascular coagulation	3	2	1	
**Cardiac disorders**	Any event within class	59	29	30	
	Aortic valve disease	3	2	1	
	Asystole	4	1	3	
	Atrial fibrillation	1	0	1	
	Atrioventricular block complete	3	2	1	
	Cardiac arrest	0	0	0	
	Heart failure	8	2	6	
	Left ventricular systolic dysfunction	1	1	0	
	Mitral valve disease	1	1	0	
	Myocardial infarction	2	1	1	
	Pericardial tamponade	8	5	3	
	Pericarditis	1	0	1	
	Right ventricular dysfunction	1	1	0	
	Tricuspid valve disease	1	1	0	
	Ventricular fibrillation	5	5	0	
	Ventricular tachycardia	1	0	1	
	Cardiac disorders - Other (specify)	19	7	12	
**Eye disorders**	Any event within class	1	0	1	
	Eye disorders – other (specify)	1	0	1	
**Gastrointestinal disorders**	Any event within class	12	1	11	
	Dysphagia	1	0	1	
	Gastric hemorrhage	2	1	1	
	Ileus	1	0	1	
	Intra-abdominal hemorrhage	3	0	3	
	Rectal hemorrhage	2	0	2	

© 2023 Stoppe C et al. *JAMA Surgery*.

Coded Category	Coded Subcategory	Overall (n=1394)	Selenium (n=697)	Placebo (n=697)	P-values
	Gastrointestinal disorders - Other (specify)	3	0	3	
**General disorders and administration site conditions**	Any event within class	12	2	10	
	Localized edema	1	0	1	
	Multi-organ failure	11	2	9	
**Hepatobiliary disorders**	Any event within class	1	0	1	
	Hepatic failure	1	0	1	
**Infections and infestations**	Any event within class	12	3	9	
	Catheter related infection	1	0	1	
	Endocarditis infective	2	1	1	
	Sepsis	6	1	5	
	Urinary tract infection	1	0	1	
	Infections and infestations - Other (specify)	2	1	1	
**Injury, poisoning and procedural complications**	Any event within class	6	6	0	
	Intraoperative hemorrhage	3	3	0	
	Intraoperative respiratory injury	1	1	0	
	Postoperative hemorrhage	1	1	0	
	Injury, poisoning and procedural complications - Other (specify)	1	1	0	
**Metabolism and nutrition disorders**	Any event within class	2	1	1	
	Acidosis	2	1	1	
**Musculoskeletal and connective tissue disorders**	Any event within class	8	4	4	
	Musculoskeletal and connective tissue disorder - Other (specify)	8	4	4	
**Nervous system disorders**	Any event within class	16	5	11	
	Aphonia	1	0	1	
	Cerebrospinal fluid leakage	1	0	1	
	Encephalopathy	1	0	1	
	Ischemia cerebrovascular	5	1	4	
	Seizure	2	1	1	
	Stroke	5	3	2	

© 2023 Stoppe C et al. *JAMA Surgery*.

Coded Category	Coded Subcategory	Overall (n=1394)	Selenium (n=697)	Placebo (n=697)	P-values
	Nervous system disorders - Other (specify)	1	0	1	
**Psychiatric disorders**	Any event within class	9	4	5	
	Delirium	9	4	5	
**Renal and urinary disorders**	Any event within class	15	6	9	
	Acute kidney injury	14	6	8	
	Renal and urinary disorders - Other (specify)	1	0	1	
**Respiratory, thoracic and mediastinal disorders**	Any event within class	24	9	15	
	Atelectasis	1	1	0	
	Bronchospasm	1	1	0	
	Pleural effusion	1	0	1	
	Pneumonitis	2	2	0	
	Pneumothorax	2	1	1	
	Respiratory failure	10	2	8	
	Respiratory, thoracic and mediastinal disorders - Other (specify)	7	2	5	
**Skin and subcutaneous tissue disorders**	Any event within class	1	1	0	
	Skin ulceration	1	1	0	
**Vascular disorders**	Any event within class	12	8	4	
	Hematoma	2	1	1	
	Hypotension	1	1	0	
	Peripheral ischemia	1	0	1	
	Thromboembolic event	2	2	0	
	Vascular disorders - Other (specify)	6	4	2	

[*]Totals don't add up, because some patients had multiple SAEs.

[**] P-value based on Fisher's Exact test.

© 2023 Stoppe C et al. *JAMA Surgery*.

eFigure 1: Intervention Scheme

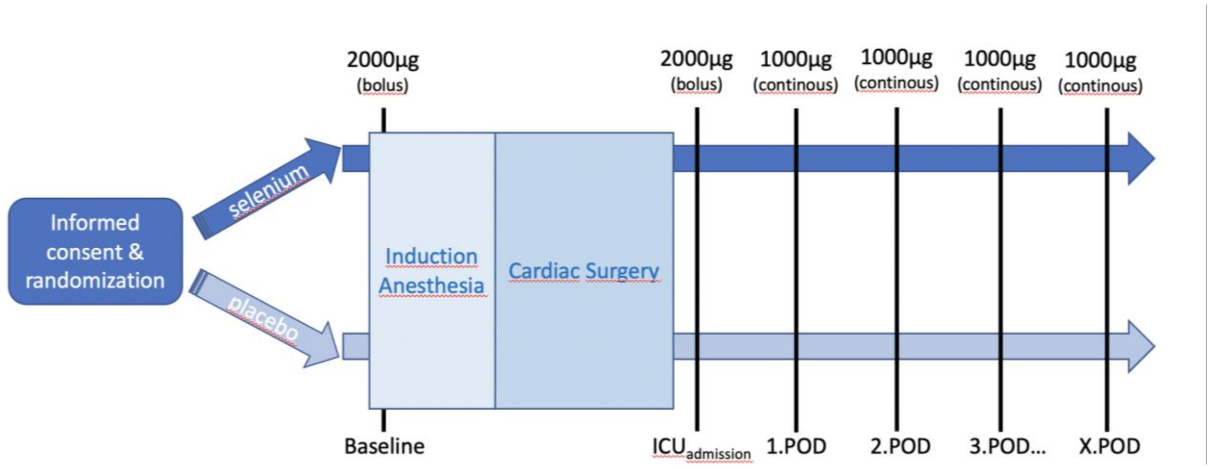

Eligible patients were randomly assigned to receive either a perioperative treatment with sodium selenite placebo. The treatment was continued on the ICU until discharge or death. POD: postoperative day

© 2023 Stoppe C et al. *JAMA Surgery*.

eFigure 2: Six-month Kaplan-Meier Survival Curve

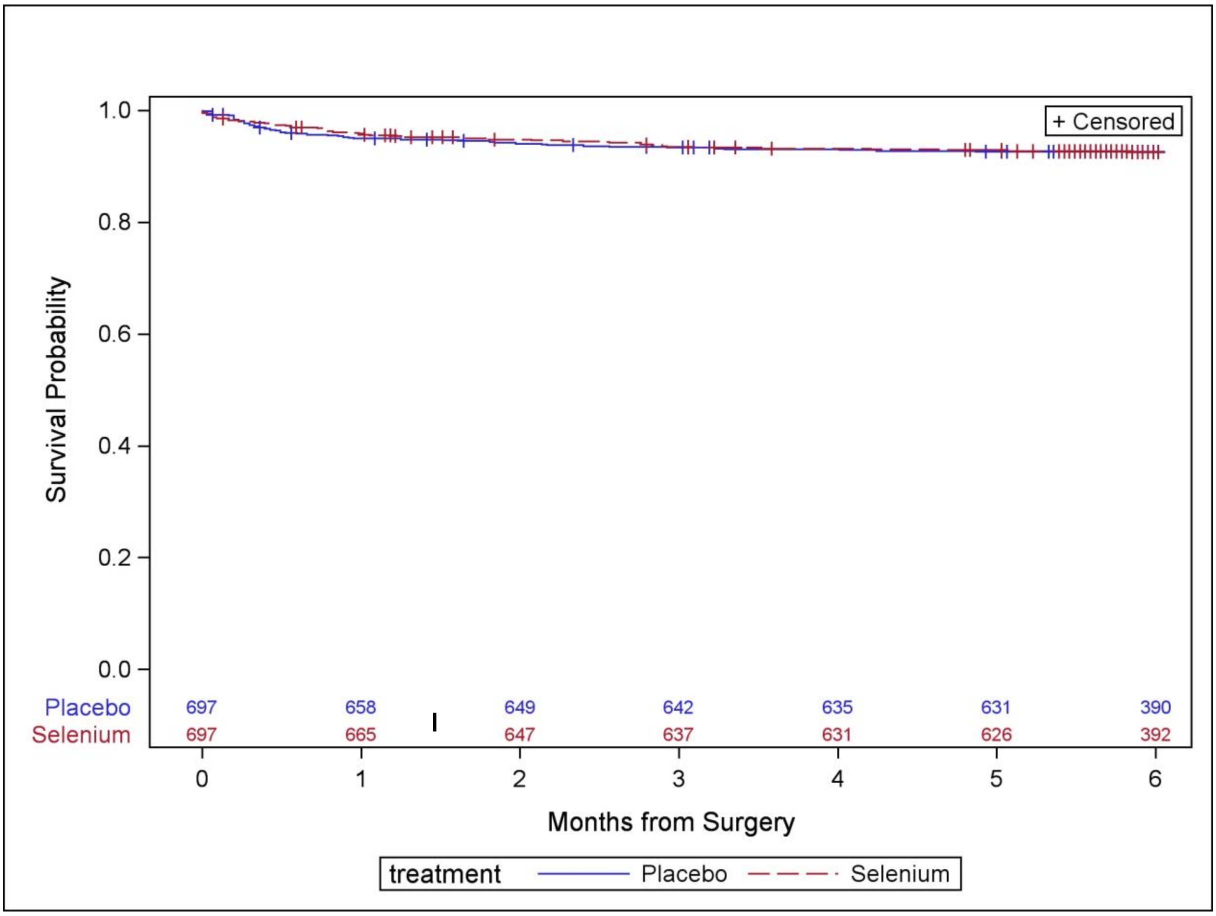

© 2023 Stoppe C et al. *JAMA Surgery*.

eFigure 3: Product-Limit Survival Estimates

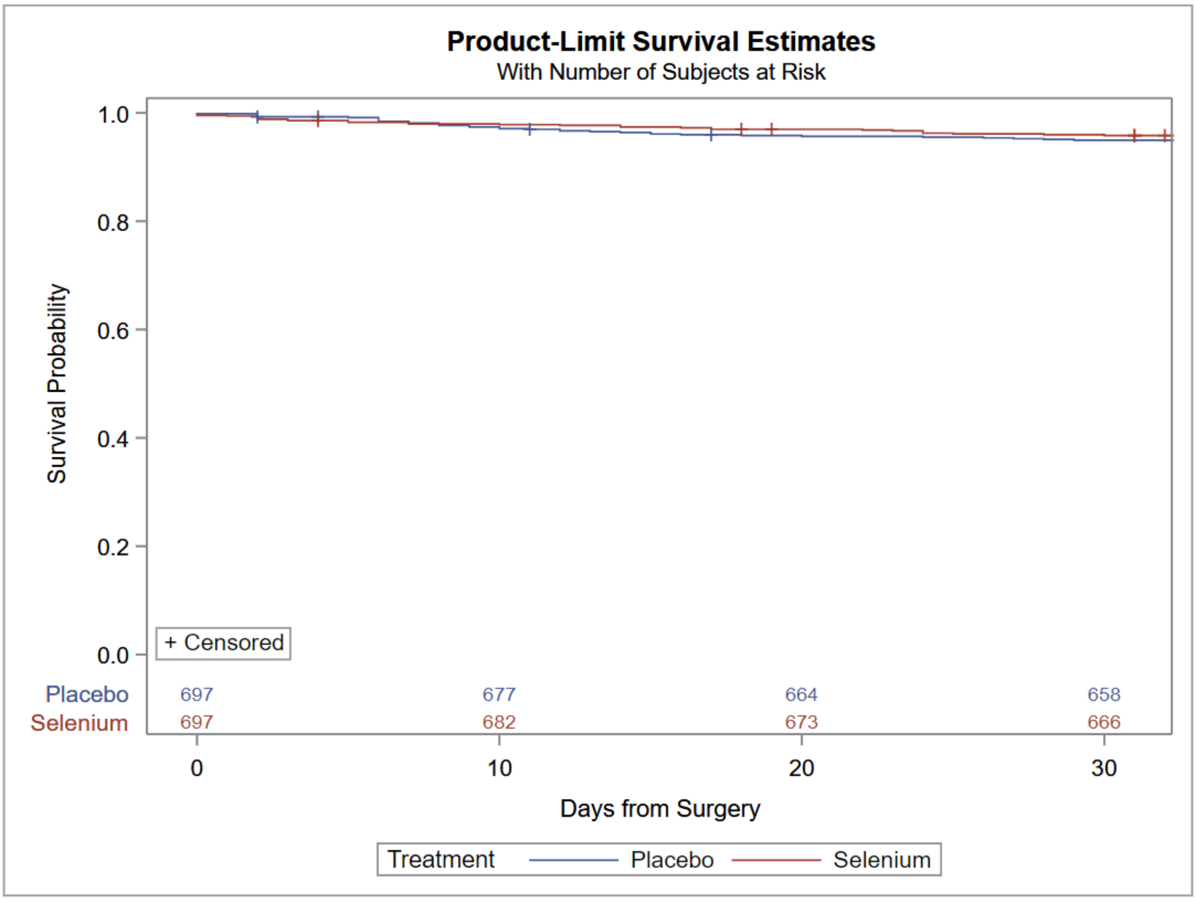

© 2023 Stoppe C et al. *JAMA Surgery*.

eAppendix. Supplemental Data to Figure 3A and 3B

Exploratory Statistical Analysis to Figure 1A Differences in selenium levels

Time points	Selenium	Placebo	p values
Baseline	65.1 [54.7 to 79.2]	74.8 [57.7 to 104.7]	0.11
POD0	114.8 [102.6 to 153.3]	55.5 [45.6 to 71.8]	<.0001
POD1	110.6 [74.0 to 131.4]	60.6 [45.8 to 74.0]	<.0001
POD2	102.5 [90.6 to 116.6]	55.0 [43.9 to 77.7]	<.0001
POD3	110.0 [100.7 to 122.3]	63.6 [47.6 to 90.7]	0.002
POD4	124.8 [114.1 to 141.1]	53.8 [47.5 to 69.0]	<.0001
POD5	128.7 [115.3 to 148.7]	44.5 [36.4 to 54.6]	0.0005
POD6	151.9 [136.0 to 172.4]	63.2 [44.0 to 80.1]	0.003
POD7	158.8 [130.8 to 198.0]	62.3 [47.3 to 98.5]	0.003
POD8	128.2 [118.6 to 161.5]	64.4 [47.1 to 100.8]	0.14
POD09	181.5 [129.1 to 185.2]	43.3 [42.4 to 95.4]	0.03
POD010	159.7 [130.9 to 181.2]	41.6 [38.4 to 44.8]	0.08

Median [Q1, Q3] reported

POD: Postoperative Day

Exploratory Statistical Analysis to Figure 1B Difference in Glutathionperoxidase activity

Time points	Selenium	Placebo	p values
Baseline	235.4 [191.3 to 295.3]	226.5 [201.8 to 287.0]	0.75
POD0	208.3 [152.8 to 280.1]	170.1 [135.7 to 274.4]	0.27
POD1	231.1 [214.8 to 283.6]	231.6 [208.7 to 274.7]	0.59
POD2	270.9 [252.8 to 285.7]	266.4 [244.8 to 291.6]	0.63
POD3	287.3 [265.2 to 301.6]	274.9 [241.7 to 295.2]	0.17
POD4	280.8 [272.1 to 296.6]	261.2 [222.1 to 286.4]	0.09
POD5	275.3 [266.7 to 282.9]	247.0 [189.9 to 284.2]	0.25
POD6	271.0 [264.1 to 273.8]	240.9 [200.9 to 288.4]	0.56
POD7	279.0 [271.8 to 290.9]	182.9 [172.3 to 305.2]	0.32
POD8	280.1 [265.5 to 302.0]	227.1 [196.7 to 275.1]	0.14
POD09	276.7 [264.1 to 276.9]	217.3 [188.2 to 317.6]	0.46
POD010	261.9 [236.7 to 277.8]	240.5 [175.2 to 305.9]	1.00

Median [Q1, Q3] reported

POD: Postoperative Day

© 2023 Stoppe C et al. *JAMA Surgery.*

eReferences

[1] Roques F, Michel P, Goldstone AR, Nashef SA. The logistic EuroSCORE. *Eur Heart J.* 2003;24(9):881-2.

[2] Ely EW, Margolin R, Francis J, et al. Evaluation of delirium in critically ill patients: validation of the Confusion Assessment Method for the Intensive Care Unit (CAM-ICU). *Crit Care Med.* 2001;29(7):1370-9.

[3] Moghaddam A, Heller RA, Sun Q, et al. Selenium Deficiency Is Associated with Mortality Risk from COVID-19. *Nutrients.* 2020;12(7):2098.

[4] Flohé L, Günzler WA. Assays of glutathione peroxidase. *Methods Enzymol*. 1984;105:114-21.

[5] Fine JP, Gray RP. A Proportional Hazards Model for the Subdistribution of a Competing Risk. *J Am Stat Assoc.* 1999;94: 496-509.

© 2023 Stoppe C et al. *JAMA Surgery.*
