# Supplementary material for: Effect of High-Dose Selenium on Postoperative Organ Dysfunction and Mortality in Cardiac Surgery Patients: The SUSTAIN CSX Randomized Clinical Trial
Source: JAMA Surg. 2023 Jan 11;158(3):235–44. doi: 10.1001/jamasurg.2022.6855 (PMC9857635; doi:10.1001/jamasurg.2022.6855)
Supplement: Supplement 3. — Nonauthor Collaborators. The SUSTAIN CSX Study Collaborators [file jamasurg-e226855-s003.pdf]

Supplemental Online Content: Nonauthor Collaborators
*First name, last name, and suffix (if applicable) are required and will appear in PubMed.

*Group Name(s): The SUSTAIN CSX Study Collaborators							
***First Name and Middle Initial(s)**	***Last Name**	***Suffix (eg, Jr, III)**	Academic Degrees	Institution	Location (city, state/province, country)	Role or Contribution, eg, chair, principal investigator	Group (if more than 1 Group listed in the byline) and/or Subgroup (eg, Steering Committee)
Gregory	Hare		M.D., Ph.D.	Li Ka Shing Knowledge Institute, St. Michael's Hospital; Departments of Anesthesiology and Physiology, University of Toronto	Toronto, Ontario, Canada	Study collaborator	
Michael WA	Chu		M.D.	London Health Sciences Centre	London, Ontario, Canada	Study collaborator	
Pierre	Voisine		M.D.	Quebec Heart and Lung Institute, Laval University	Quebec City, QC, Canada	Study collaborator	
Francois	Dagenais		M.D.	Quebec Heart and Lung Institute, Laval University	Quebec City, QC, Canada	Study collaborator	
Eric	Dumont		M.D.	Quebec Heart and Lung Institute, Laval University	Quebec City, QC, Canada	Study collaborator	
Frédérique	Jacques		M.D.	Quebec Heart and Lung Institute, Laval University	Quebec City, QC, Canada	Study collaborator	
Eric	Charbonneau		M.D.	Quebec Heart and Lung Institute, Laval University	Quebec City, QC, Canada	Study collaborator	
Jean	Perron		M.D.	Quebec Heart and Lung Institute, Laval University	Quebec City, QC, Canada	Study collaborator	
Simone	Lindau		M.D.	University Hospital Frankfurt	Frankfurt am Main, Germany	Study collaborator	
Roupen	Hatzakorizan		M.D.	McGill University Health Centre	Montreal, Quebec, Canada	Study collaborator	
Assad	Haneya		M.D.	University Hospital Schleswig-Holstein	Kiel, Germany	Study collaborator	
Georg	Trummer		M.D.	University Heart Center Freiburg Bad Krozingen	Bad Krozingen, Germany	Study collaborator	

Supplemental Online Content: Nonauthor Collaborators
*First name, last name, and suffix (if applicable) are required and will appear in PubMed.

***First Name and Middle Initial(s)**	***Last Name**	***Suffix (eg, Jr, III)**	Academic Degrees	Institution	Location (city, state/province, country)	Role or Contribution, eg, chair, principal investigator	Group (if more than 1 Group listed in the byline) and/or Subgroup (eg, Steering Committee)
Angela	Jareth		M.D.	Department of Anesthesiology and Pain Medicine, University of Toronto	Toronto, ON, Canada	Study collaborator	
Xuran	Jiang		M.Sc.	Clinical Evaluation Research Unit, Kingston Health Sciences Centre	Kingston, Canada	Study collaborator	
Ellen	Dresen		Ph.D.	Department of Anaesthesiology, Intensive Care, Emergency, and Pain Medicine, University Hospital Wuerzburg	Wuerzburg, Germany	Study collaborator	
Aileen	Hill		M.D.	Department of Anesthesiology, RWTH Aachen University, Aachen	Aachen, Germany	Study collaborator	
