# Supplementary material for: Effect of High-Dose Selenium on Postoperative Organ Dysfunction and Mortality in Cardiac Surgery Patients: The SUSTAIN CSX Randomized Clinical Trial
Source: JAMA Surg. 2023 Jan 11;158(3):235–44. doi: 10.1001/jamasurg.2022.6855 (PMC9857635; doi:10.1001/jamasurg.2022.6855)
Supplement: Supplement 4. — Data Sharing Statement [file jamasurg-e226855-s004.pdf]

Data Sharing Statement

Stoppe. Effect of High-Dose Selenium on Postoperative Organ Dysfunction and Mortality in Cardiac Surgery Patients. *JAMA Surg*. Published January 11, 2023.
doi:10.1001/jamasurg.2022.6855

Data
Data available: Yes
Data types: Deidentified participant data
How to access data: dkh2@queensu.ca
When available: With publication

Supporting Documents
Document types: None

Additional Information
Who can access the data: Researchers whose proposed use of the data has been approved.
Types of analyses: For the purpose of outcome research and quality improvement in clinical trials, all other upon request.
Mechanisms of data availability: After approval of a proposal and with a signed data access agreement.
Any additional restrictions: -
